# Sustained activation of 12/15 lipoxygenase (12/15 LOX) contributes to impaired renal recovery post ischemic injury in male SHR compared to females

**DOI:** 10.1186/s10020-023-00762-y

**Published:** 2023-12-04

**Authors:** Riyaz Mohamed, Jennifer C. Sullivan

**Affiliations:** Department of Physiology, Medical College of Georgia at Augusta University, Augusta, Georgia 30912 United States

**Keywords:** Acute kidney injury, Lipoxygenase, Gender, Renal recovery, Sex difference, Ischemia

## Abstract

**Background:**

Acute kidney injury (AKI) due to ischemia-reperfusion (IR) is a serious and frequent complication in clinical settings, and mortality rates remain high. There are well established sex differences in renal IR, with males exhibiting greater injury following an ischemic insult compared to females. We recently reported that males have impaired renal recovery from ischemic injury vs. females. However, the mechanisms mediating sex differences in renal recovery from IR injury remain poorly understood. Elevated 12/15 lipoxygenase (LOX) activity has been reported to contribute to the progression of numerous kidney diseases. The goal of the current study was to test the hypothesis that enhanced activation of 12/15 LOX contributes to impaired recovery post-IR in males vs. females.

**Methods:**

13-week-old male and female spontaneously hypertensive rats (SHR) were randomized to sham or 30-minute warm bilateral IR surgery. Additional male and female SHR were randomized to treatment with vehicle or the specific 12/15 LOX inhibitor ML355 1 h prior to sham/IR surgery, and every other day following up to 7-days post-IR. Blood was collected from all rats 1-and 7-days post-IR. Kidneys were harvested 7-days post-IR and processed for biochemical, histological, and Western blot analysis. 12/15 LOX metabolites 12 and 15 HETE were measured in kidney samples by liquid chromatography–mass spectrometry (LC/MS).

**Results:**

Male SHR exhibited delayed recovery of renal function post-IR vs. male sham and female IR rats. Delayed recovery in males was associated with activation of renal 12/15 LOX, increased renal 12-HETE, enhanced endoplasmic reticulum (ER) stress, lipid peroxidation, renal cell death and inflammation compared to females 7-days post-IR. Treatment of male SHR with ML355 lowered levels of 12-HETE and resulted in reduced renal lipid peroxidation, ER stress, tubular cell death and inflammation 7-days post-IR with enhanced recovery of renal function compared to vehicle-treated IR male rats. ML355 treatment did not alter IR-induced increases in plasma creatinine in females, however, tubular injury and cell death were attenuated in ML355 treated females compared to vehicle-treated rats 7 days post-IR.

**Conclusion:**

Our data demonstrate that sustained activation 12/15 LOX contributes to impaired renal recovery post ischemic injury in male and female SHR, although males are more susceptible on this mechanism than females.

**Supplementary Information:**

The online version contains supplementary material available at 10.1186/s10020-023-00762-y.

## Introduction

Renal ischemia-reperfusion (IR) injury is a major cause of acute kidney injury (AKI), which is a frequent complication among hospitalized patients and is associated with poor clinical outcomes (Kher and Kher 2020; Al-Jaghbeer et al. 2018). Although the incidence of AKI varies depending on the definitions used and populations, AKI is estimated to occur in up to 15% of all hospitalized patients, and in over 50% of critically ill patients in intensive care units (Hoste et al. 2015). AKI increases in-hospital morbidity and mortality (Chertow et al. 2005; Schneider et al. 2010), and predisposes patients to the later development of chronic kidney disease (CKD) (Mammen et al. 2012), cardiovascular diseases (Hsu et al. 2016), and end stage renal disease which ultimately leads to renal failure. Clinical and pre-clinical studies have reported that there are sex differences in the incidence and mortality rates of AKI where males have a greater tendency to develop AKI with higher mortality rates than females (Mohamed et al. 2022; Neugarten et al. 2018; Lima-Posada et al. 2017; Toth-Manikowski et al. 2021). Despite advances in medical technology in recent decades, effective therapies and preventative strategies for AKI are still lacking in both sexes. A better understanding of the cellular mechanisms that mediate renal injury and recovery is required to design therapies to treat ischemic AKI in both males and females.

Lipoxygenases (LOXs) are a family of iron-containing enzymes that act on arachidonic acid to produce hydroxyeicosatetraenoic acids (HETEs) (Kuhn et al. 2015). 12/15 LOX is constitutively expressed in reticulocytes, peritoneal macrophages, and epithelial cells and has an important role in human inflammatory diseases (Dobrian et al. 2011). Human 15-LOXs as well as the leukocyte-type 12-LOX have high homology and are classified as 12/15 LOX since they can form both 12-HETE and 15-HETE from arachidonic acid (Brash 1999). Moreover, 12/15 LOX derived lipid metabolites, 12/15 HETE, regulate cell metabolism including activation of ER stress (Cole et al. 2012; Elmasry et al. 2018), oxidative stress, cell death (Li et al. 2018) and promote inflammation (Kulkarni et al. 2021). Indeed, activation of 12/15 LOX and its metabolites increase renal inflammation and fibrosis in a male murine model of unilateral ureteral obstruction (Montford et al. 2022). Moreover, pharmacological inhibition of 12/15 LOX reduces renal inflammation in a number of ischemic conditions in males, including renal IR injury (Kar et al. 2020), gentamicin-induced AKI (Sharma et al. 2022), and acute lung injury (Rossaint et al. 2012; Faulkner et al. 2015). However, the contribution of 12/15 LOX activation on renal recovery from ischemic injury in either males or females has not been fully elucidated.

We previously reported that male and female SHR have comparable injury in response to renal ischemia 24 h post-IR (Mohamed et al. 2020). However, males exhibit sustained tubular injury with impaired renal recovery 7-days post-IR compared to females (Mohamed et al. 2022). The cellular mechanisms mediating impaired recovery of renal function in male SHR following ischemic injury is not well understood. Since 12/15 LOX plays a role in the pathogenesis of cardiovascular and renal diseases in males (Li et al. 2018; Montford et al. 2022), the goal of the current study was to test the hypothesis that sustained activation of 12/15 LOX contributes to the delayed recovery from renal IR injury in males compared to females. Our results demonstrate that 12/15 LOX activation is sustained in parallel with delayed recovery in males. Furthermore, inhibition of 12/15 LOX activation attenuated renal ER stress, cell death, inflammation, and improved post-IR renal recovery in male SHR. Interestingly, ML355 treatment did not alter IR-induced increases in plasma creatinine and renal 12-HETE levels in females, however, tubular injury and renal cell death were attenuated compared to vehicle treated rats 7 days post-IR.

## Materials and methods

### Animals

Male and female SHR (12–13 weeks of age) were purchased from Envigo Laboratories (Indianapolis, IN). SHR were selected for the current study based on our previous work showing that SHR exhibit a sex difference in renal recovery 7-days post-IR (Mohamed et al. 2022), which allows us to study sex specific mechanism mediating renal post-IR. Rats were housed in temperature (20–26 °C) and humidity (30–-70%) controlled, 12:12 h light-cycled conventional animal quarters. Rats were provided *ad libitum* access to water and standard 18% protein rodent chow (Envigo Teklad, 2918). The Institutional Animal Care and Use Committee of Augusta University approved all of the protocols and procedures using animals (approval number 2014-048).

### Warm bilateral renal ischemia reperfusion

Rats (n = 5–6) were anesthetized with ~ 2% isoflurane and placed on a heating pad to maintain body temperature at 37 °C. The left and right kidneys were accessed by flank incisions. The kidneys were exposed, and both renal arteries were carefully separated from the renal vein via manual blunt dissection and clamped with microserrefines (Fine Science Tools, Foster City, CA) for 30 min as previously reported (Mohamed et al. 2020). The duration of the ischemic insult was chosen based on previous studies where 15 min of renal ischemia resulted in minimal injury and 45 min of ischemia resulted in ~ 80% mortality within 3 days in SHR (Crislip et al. 2020). Reperfusion of the kidney was confirmed visually upon release of the clamps. As a control, sham-operated animals were subjected to the same surgical procedure except the renal arteries were not clamped. Surgical wounds were closed using 4 − 0 polypropylene suture and wound clips. Rats were given 1 ml of warm saline, intraperitoneal to replace any fluid loss. Rats were kept warm until they regained consciousness and buprenorphine (0.9 mg/kg sc) was administered as an analgesic. Rats were allowed to recover for 1 or 7 days in conventional animal housing before being deeply anesthetized with ~ 2% isoflurane. The depth of anesthesia was checked by tail pinch and pedal reflex before a midline incision was made. A terminal blood sample was collected from the abdominal aorta as rats were rapidly exsanguinated, followed by a thoracotomy. Kidneys were harvested for histological and biochemical analyses.

To assess the role of 12/15 LOX in recovery from renal IR, additional male and female SHR (n = 5–6) were randomized to receive intraperitoneal injection of the specific 12/15 LOX inhibitor ML355 (30 mg/kg body weight) or vehicle (saline + 0.02% DMSO) 1 h prior to sham/IR surgery and every other day up to 7 days post-IR (Adili et al. 2017). The dose of ML355 was chosen based on pre-experimental screening and route of administration was chosen based on published literature (Adili et al. 2017). Blood samples were collected at baseline and 1-day post-IR via tail vein puncture. Animals were allowed to recover for 7 days in conventional animal housing before being anesthetized with ~ 2% isoflurane as described above. A terminal blood sample and kidneys were harvested.

### Blood pressure (BP) measurement

To determine whether IR surgery or 12/15 LOX inhibition affected BP, systolic BP was measured at baseline, 1-, 3- and 7-days post-IR in vehicle- and ML355-treated male and female SHR using tail cuff as described previously (Tipton et al. 2014; Pollock and Rekito 1998). Rats were acclimated to the tail cuff technique for three consecutive days before baseline data collection. The systolic BP of each rat was determined by the average of five independent readings with at least a 1-minute break between each inflation of the pressure cuff.

### Biochemical measurements

#### Assessment of renal function

Plasma and urine creatinine concentrations and blood urea nitrogen (BUN) were measured using commercially available kits (BioAssay Systems, Hayward, CA; Quantichrome Creatinine Assay Kit, Cat# DICT-500, Lot number CA04A06; Quantichrome Urea Assay Kit, Cat # DIUR-100, Lot number CA02A12)(Crislip et al. 2017).

#### Measurement of 12/15 LOX activity

Activity of 12/15 LOX was evaluated by measuring the amount of the major metabolic product of 12/15 LOX, 12-HETE in the plasma by ELISA (Abcam, USA) and 12-HETE and 15-HETE in the kidney by LC/MS. Renal HETE levels were measured in the Lipidomics Core Facility at Wayne State University (Detroit, MI) as previously described (Othman et al. 2013; Maddipati and Zhou 2011; Maddipati et al. 2014). Briefly, 2 mg of kidney homogenate was adjusted to a maximum volume of 1 ml and spiked with a mixture of internal standards consisting of 15(S)-HETE-d8, Leukotriene B4-d4, Resolvin D2-d5, 14(Cole et al. 2012)-EpETrE-d11, and prostaglandin E1-d4 (5 ng each) and mixed thoroughly. Samples were then extracted for PUFA metabolites using C18 extraction columns as previously described (Maddipati and Zhou 2011; Maddipati et al. 2014). Briefly, the internal standard spiked samples were applied to conditioned C18 cartridges, washed with water followed by hexane, and dried under vacuum. The cartridges were eluted with 0.5 ml methanol. The eluate was dried under a gentle stream of nitrogen. The residue was re-dissolved in 50 μl methanol-25 mM aqueous ammonium acetate (1:1) and subjected to LC-MS analysis performed on a Prominence XR system (Shimadzu) using Luna C18 (3 μ, 2.1 × 150 mm) column. The data were collected using Analyst 1.5.2 software and the Multiple Reaction Monitoring (MRM) transition chromatograms were quantitated by MultiQuant software (both from ABSCIEX). The internal standard signals in each chromatogram were used for normalization, recovery, and relative quantitation of each analyte. The average recovery using this procedure was > 90%. 12/15 HETE metabolite concentrations were normalized to the initial kidney weight and levels were directly compared between male and females.

#### Lipid peroxidation

The extent of lipid oxidation in the kidney and plasma were evaluated by measuring malondialdehyde (MDA) concentrations using thiobarbituric acid reactive substances (TBARS) assay kit (Cayman Chemical) according to manufacturer instruction.

#### TNF-α measurement

Plasma levels of TNF-α were measured using a commercial ELISA assay according to the manufacturer instructions (Abcam, Cambridge, MA).

### Western blot analysis

Thirty μg of renal cortex was isolated, homogenized, and used for Western blot analysis as previously described (Mohamed et al. 2022; Sasser et al. 2007). Briefly, the homogenate was resolved on 4–20% Tris-glycine-SDS gels (Bio-Rad, Hercules, CA) and proteins were transferred to PVDF membranes (MilliporeSigma, Burlington, MA). Protein expression was determined with two-color immunoblots using primary antibodies to GPR78 (cat# ab21685; 1:1000 dilution, Abcam, USA), phosphorylated PERK (cat# 3179 S; 1:1000 dilution, Cell Signaling Technology, USA), total PERK (cat# 3192 S; 1:1000 dilution, Cell Signaling Technology, USA) and CHOP (cat# 28,955; 1:1000 dilution, Cell Signaling Technology, USA). Specific protein bands were detected using an Odyssey infrared imager (LI-COR Biosciences, Lincoln, NE) with AlexaFluor 680 and IRDye800 (Molecular Probes, Eugene, OR) conjugated secondary antibodies. Protein concentrations were determined by standard BCA reagent (Thermo Scientific) using BSA as the standard. Protein loading was normalized to *β*-actin (Sigma, St. Louis, MO) consistent with standard laboratory practice.

### Histological assessment of tubular injury

Kidneys were bisected transversely with a razor blade, fixed in buffered 10% formalin overnight and then embedded in paraffin wax. For assessment of renal tubular injury, 5 μM sections were stained with hematoxylin and eosin (H&E) following the protocol provided by the manufacturer (Leica Biosystems; Buffalo Grove, IL). Renal tubular injury was quantified by an investigator blinded to the hypothesis and the rat sex and treatment by assigning a score based on the percentage of renal tubules showing epithelial cell necrosis, brush-border loss, cast formation, and apoptotic bodies (Mohamed et al. 2022, Ranganathan et al. 2013). Renal tubular injury scores were assessed based on the following scale indicating the percentage of tubules showing signs of injury: 0 = normal; 1 = < 25%; 2 = 25–50%; 3 = 51–75%; 4 = > 75%. 10 fields at 20X magnification were examined and data were averaged for each animal. Stained sections were photographed using an Olympus BX40 microscope (Olympus America, Melville, NY) on a bright-field setting fitted with a digital camera (Olympus DP12; Olympus America).

### TUNEL staining

TUNEL staining was performed to assess cell death in renal tissue slices using the ApopTag plus Peroxidation in situ Apoptosis Detection kit according to the manufacturer instructions (EMD Millipore; cat # S7101). Briefly, 5 μM kidney sections were deparaffinized, hydrated, and washed with PBS. Sections were digested with proteinase K for 15 min at 24 °C. Slides were then washed with PBS, and endogenous peroxidase activity was quenched with 3% H_2_O_2_ in methanol. Slides were washed again and incubated with TdT labeling reaction mix at 37 °C for 1 h and then with anti-digoxigenin peroxidase. The color was developed using peroxidase substrate solution. Slides were washed, counterstained, and mounted with Permount. TUNEL positive cells were counted and quantified by an investigator blinded to the hypothesis, rat sex and treatment. Brown nuclei indicate TUNEL-positive cells.

### Macrophage immunostaining

To quantify renal macrophage infiltration, formalin-fixed kidneys were sectioned and 5 μM sections were incubated in the absence or presence of primary antibodies to the macrophage marker CD68 (R&D, cat #; 1:400 dilution) in humidified chambers overnight at 4 °C. The next day, kidney sections were washed with phosphate buffer saline (PBS) and incubation with HRP-conjugated donkey anti-rabbit IgG (Jackson ImmunoResearch Laboratories, West Grove, PA) for 1 h at room temperature. Color was developed after incubation with DAB reagent (Vector Lab, Burlingame, CA). Macrophages were quantified by an investigator blinded to the hypothesis, rat sex and treatment utilizing 10 fields at 20X magnification. Brown nuclei indicate CD68-positive cells macrophages.

### Real-time quantitative reverse transcriptase PCR

Total RNA was extracted from snap-frozen kidney cortex using RNeasy Mini Kits (Qiagen, Germantown, MD) according to the manufacturer protocol as described previously (Mohamed et al. 2016). RNA (2 μg) was reverse transcribed to complement DNA using iScript. Reverse Transcription Supermix for RT-qPCR (BioRad, Hercules, CA). The product was diluted to a volume of 150 μl, and 5 μl aliquots were used for amplification. Real-time PCR was performed on a StepOne real-time instrument (Applied Biosystems, Foster City, CA) using iTaq Universal SYBR Green Supermix (BioRad, Hercules, CA) and gene specific primers from IDT: rat 12 LOX 5’TGGCTAAGATCTGGGTCCGA3’; 5’AACGGATGTGCGGAACTAGG3’), rat TNF-α (5’AAATGGGCTCCCTCTCATCAGTTC3’; 5’TCTGCTTGGTGGTTTGCTACGAC3) or Qiagen: rat IL-1β (Cat # QT00181657) and rat IL-6 (Cat # 330,001). The amount of DNA was normalized to the rat GAPDH signal amplified in a separate reaction (Cat # QT00199633).

### Statistical analysis

All data are presented as mean ± SEM. Statistical analyses were performed using GraphPad Prism version 9.0 software (GraphPad Software, La Jolla, CA). Same group comparisons were carried out using repeated measure ANOVA. Multiple group comparisons and sex comparisons were carried out using two-way ANOVA. *P* < 0.05 was considered as significantly different.

## Results

### 12/15 LOX is upregulated in male, but not female SHR 7 days post-IR

**Fig. 1:**
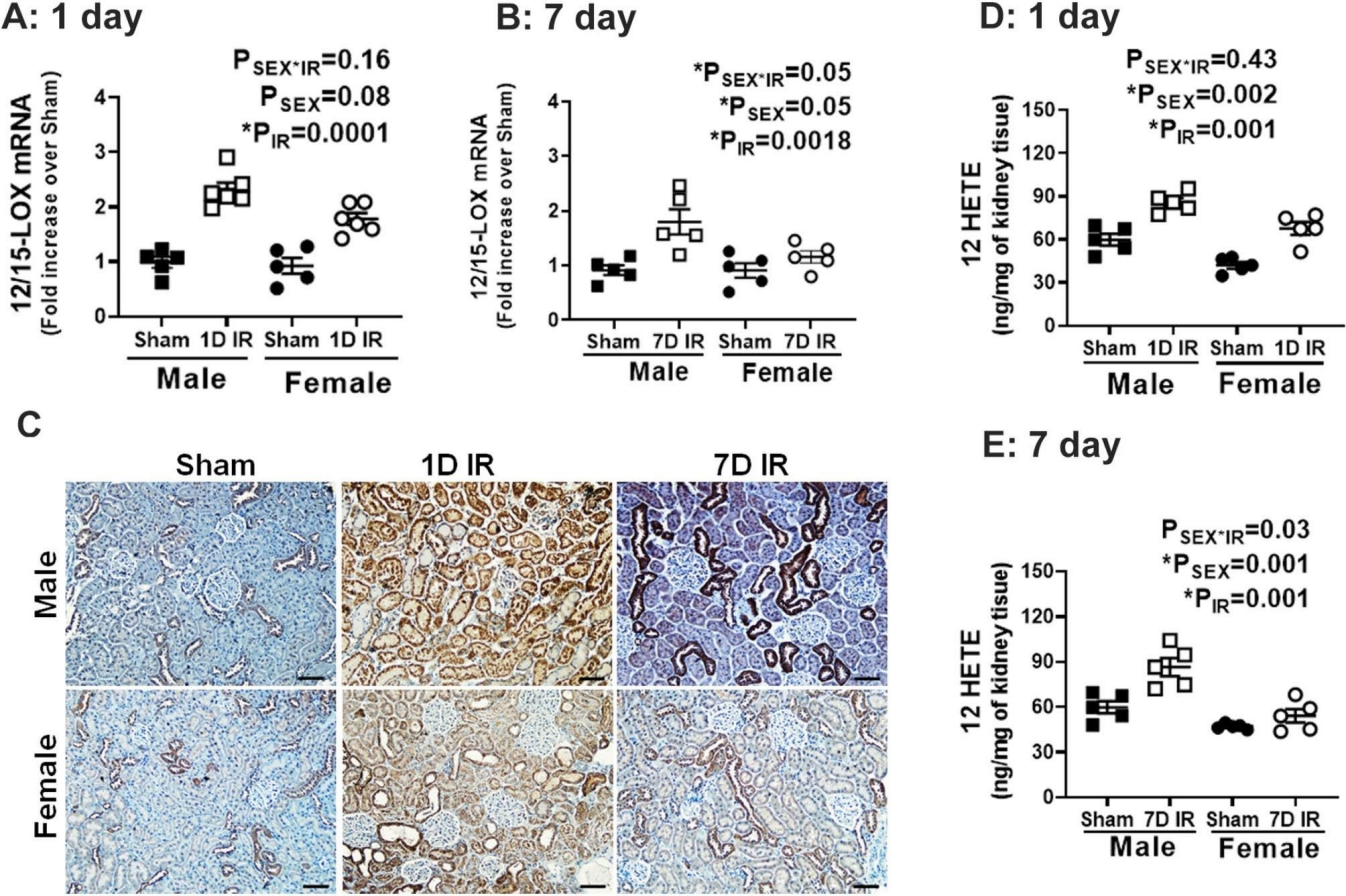
Renal 12/15 LOX activation is sustained in males 7 days post-IR. 13-week-old male and female SHR were randomized to sham or 30 min bilateral ischemia reperfusion (IR). Following 1 or 7 days, renal 12/15 LOX mRNA expression was measured by RT-PCR (Panel **A**, 1 day; Panel **B**, 7 days). 12 LOX protein expression was measured by immunohistochemical analysis: 20x magnification, scale bar 100 μM (Panel **C**). 12/15 LOX bioactive metabolite 12 HETE was measured in kidney tissue by LC/MS (Panel **D**, 1 day; Panel **E**, 7 days). Data are expressed as mean ± SEM with individual animal data indicated by the symbols, n = 5–6. Filled symbols indicate sham animals, open symbols indicate ischemia, males are represented by squares and females by circles. Data were compared via 2-way ANOVA with *P* < 0.05 considered significant

We have previously shown that male SHR exhibit impaired recovery of renal function 7 days post-IR compared to females (Mohamed et al. 2022). To begin to assess a potential role for 12/15 LOX in the delayed recovery of renal function in males, we first assessed activation of 12/15 LOX in male and female SHR 1- and 7-days post-IR by measuring renal 12/15 LOX expression by RT-PCR and immunostaining and its metabolite 12/15 HETE by LC-MS. IR significantly increased renal 12/15 LOX mRNA expression to a comparable degree in both male and female SHR 1-day post-IR compared to respective sham controls (P_IR_=0.0001; P_sex_=0.08; P_sex*IR_=0.16; Fig. 1A). 12/15 LOX mRNA expression remained upregulated 7 days post-IR only in males (P_IR_=0.0018; P_sex_=0.05, P_sex*IR_=0.05; Fig. 1B). Immunohistochemical analysis confirmed sustained upregulation of 12/15 LOX only in males, and further showed that increased expression of 12/15 LOX was localized in proximal tubular cells (Fig. 1C). Activated 12/15 LOX catalyzes the production of the stable bioactive metabolite 12 and15 hydroxyeicosatetraenoic acid (12/15-HETE) from arachidonic acid (Othman et al. 2013). Consistent with increased 12/15 LOX post-IR, its downstream bioactive metabolite 12 HETE is increased in the kidney (P_IR_=0.001; P_sex_=0.002, P_sex*IR_=0.43: Fig. 1D) and plasma (P_IR_=0.0001; Psex = 0.47, Psex*IR = 0.58: Supplementary Fig. 1A) of male and female SHR 1-day post-IR. Moreover, 12 HETE remained elevated in the kidney (P_IR_=0.001; P_sex_=0.001, P_sex*IR_=0.03) and plasma (P_IR_=0.002; P_sex_=0.0001, P_sex*IR_=0.002) 7 days post-IR only in males (Fig. 1E and Supplementary Fig. 1SB). However, renal 15-HETE levels were not altered either 1-day (P_IR_=0.15; P_sex_=0.06, P_sex*IR_=0.78) or 7-days post-IR (P_IR_=0.86; P_sex_=0.08, P_sex*IR_=0.92) and levels were comparable between the sexes (Supplementary Fig. 2SB).

### 12/15 LOX inhibition improves recovery of kidney function in male SHR post-IR

**Fig. 2:**
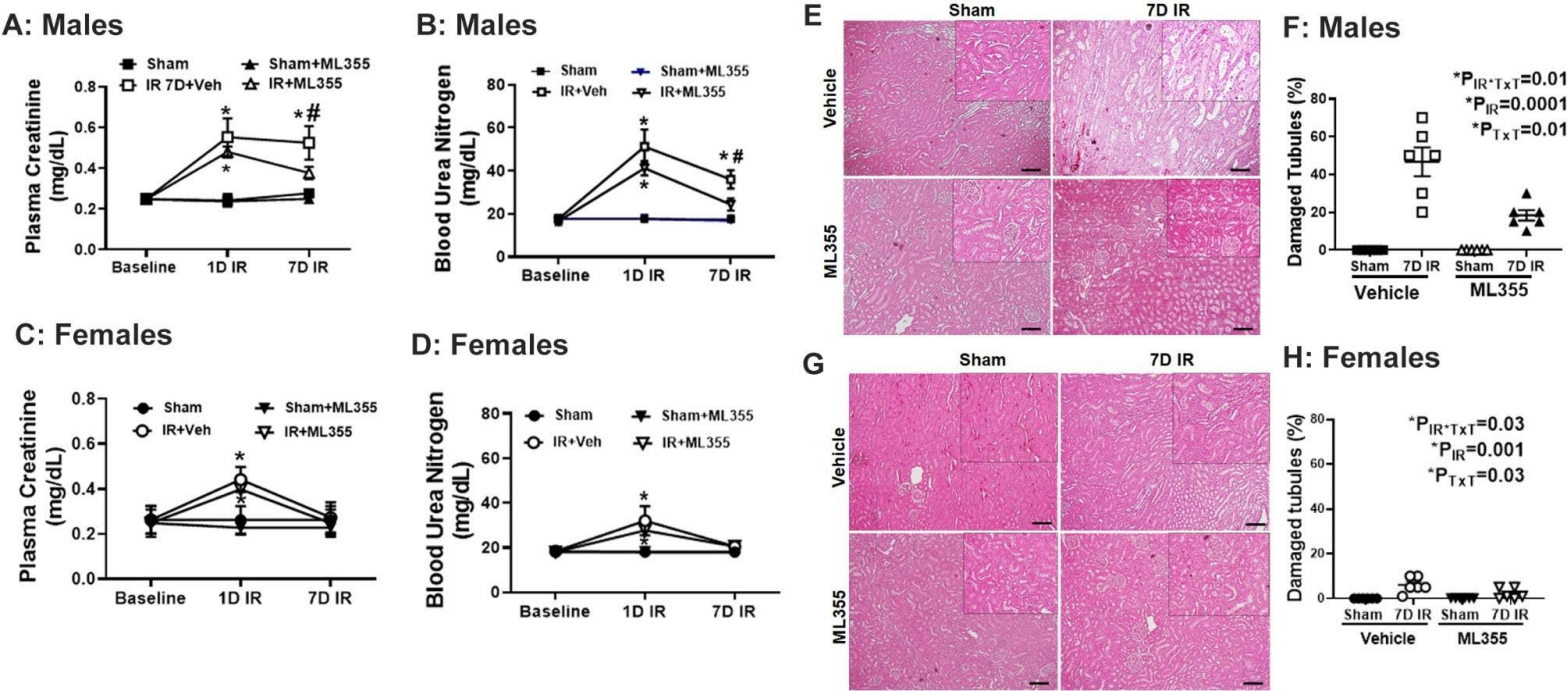
Specific 12/15 LOX inhibition with ML355 improved renal histology and function in males 7 days post-IR. Plasma creatinine and BUN were measured in samples collected 1- and 7-days post-IR in 13-week-old male (Panels **A** & **B**) and female SHR (Panels **C**&**D**) treated with vehicle or 12/15 LOX inhibitor ML355 1 h prior to sham/IR and every other day up to 7 days post-IR. Tubular injury was assessed via histological analysis of kidneys collected 7 days post-IR treated with vehicle or ML355. Representative images are shown for males (Panel **E**) and females (Panel **G**) with the mean pathological scoring data in Panels **F** & **H**. 20x magnification, scale bar 100 μM. Data are expressed as mean ± SEM, n = 5–6 rats in each group with individual animal data indicated by the symbols. Filled symbols indicate vehicle or ML355 treated sham animals, open symbols indicate vehicle or ML355-treated IR animals, males are represented by squares and upward triangle and females by circles and downward triangle. * Indicates *p* < 0.05 vs. respective sham; # indicates *p* < 0.05 vs. ML355 treated IR rat at respective time point. Data were compared via 2-way ANOVA with *P* < 0.05 considered significant

To assess the contribution of 12/15 LOX activation on impaired recovery of renal function post-IR in males, additional male and female SHR were treated with vehicle or the specific 12/15 LOX inhibitor ML355 (30 mg/kg body weight) as previously described (Adili et al. 2017). All rats were randomized to sham operation or bilateral ischemia, plasma was collected at baseline and 1 day post-IR and a terminal plasma sample and kidneys were isolated 7 days post-IR. Male rats treated with vehicle exhibited increases in renal injury 1 day post-IR, with sustained renal dysfunction indicated by elevated plasma creatinine (P_IR_=0.001) and BUN (P_IR_=0.001) 7 days post-IR. 12/15 LOX inhibition with ML355 did not alter IR-induced increases in plasma creatinine (P_TxT_=0.45; P_TxT* IR_ =0.5) or BUN (P_TxT_=0.27; P_TxT* IR_ =0.27) 1 day post-IR in males (Fig. 2A **& B**). However, ML355 treatment exhibited an improvement in renal function 7 days post-IR, but injury remained greater than sham controls (Pcr: P_IR_=0.0001; P_TxT_=0.04; P_TxT*IR_ =0.04, BUN: P_IR_=0.0004; P_TxT_=0.04; P_TxT* IR_ =0.05). As with males, vehicle treated females exhibited an increase in plasma creatinine (P_IR_=0.0001) and BUN (P_IR_=0.01) 1-day post-IR, but in contrast to males, indices of renal injury returned to sham levels by 7-days post-IR (Pcr: P_IR_= 0.59; BUN: P_IR_= 0.12; Fig. 2C **& D**). Treatment with ML355 did not alter IR induced-increases in plasma creatinine or BUN 1-day post-IR (Pcr: P_TxT_=0.09, P_TxT* IR_ =0.85; BUN: P_TxT_=0.98; P_TxT* IR_ =0.61). Moreover, ML355 did not change recovery of renal function 7-days post-IR in females (Pcr: P_IR_= 0.59; P_TxT_=0.28; P_TxT* IR_ =0.85; BUN: P_IR_= 0.12; P_TxT_=0.99; P_TxT* IR_ =0.99; Fig. 2C **& D**). Neither vehicle nor ML355 treatment altered plasma creatinine levels nor BUN in sham operated controls.

Consistent with an improvement in renal function in male SHR treated with 12/15 LOX inhibitor 7 days post-IR, histological analysis of ML355 treated kidneys showed reduced epithelial cell brush boarder loss, tubular dilation, and protein cast formation in the outer strip of the outer medulla compared to vehicle treated rats (Fig. 2E & F). Tubular injury scores were significantly lower in ML355 treated males compared to vehicle treated rats (P_IR_= 0.0001; P_TxT_=0.01; P_TxT*IR_=0.01). Tubular damage was minimal in vehicle treated females (Fig. 2G & H); however, injury was further attenuated with ML355-treatment (P_IR_= 0.001; P_TxT_=0.03; P_TxT*IR_=0.03).

**Fig. 3:**
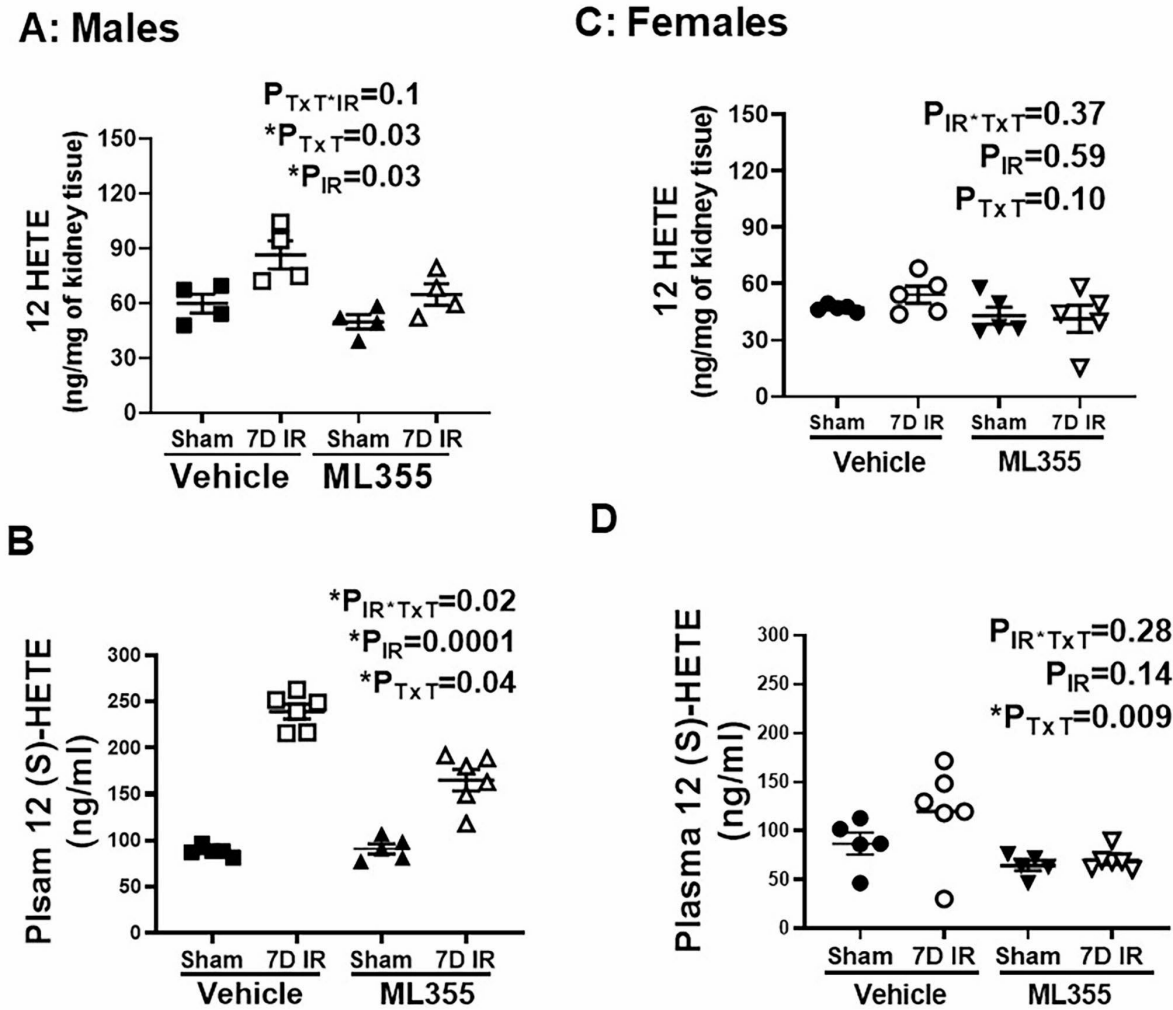
ML355 treatment reduced 12/15 HETE in males 7 days post-IR. Amount of the major metabolic product of 12/15 LOX, 12/15 HETE, was measured in 13-week-old male (Panels A&B) and female (Panels **C**&**D**) SHR treated with vehicle or 12/15 LOX inhibitor ML355 1 h prior to sham/IR and every other day up to 7 days post-IR in plasma by ELISA (Panels **A** & **C**) and in kidney by LC/MS (Panels **B** & **D**). Data are expressed as mean ± SEM, n = 5–6 rats in each group with individual animal data indicated by the symbols. Filled symbols indicate vehicle or ML355 treated sham animals, open symbols indicate vehicle or ML355-treated IR animals, males are represented by squares and upward triangle and females by circles and downward triangle. Data were compared via 2-way ANOVA with *P* < 0.05 considered significant

Experiments further confirmed inhibition of 12/15 LOX by ML355 at the termination of the study by measuring the 12/15 LOX bioactive metabolite 12 HETE in all rats. 12-HETE metabolite levels were significantly lower in the kidney (P_IR_= 0.03; P_TxT_=0.03; P_TxT* IR_ =0.1) and plasma (P_IR_= 0.0001; P_TxT_=0.04; P_TxT*IR_=0.02) of male ML355-treated rats compared to respective vehicle treated groups 7-days post-IR (Fig. 3A & B). ML355 did not alter renal 12-HETE metabolite levels in females (P_TxT_=0.10; P_TxT*IR_=0.37), although plasma levels were decreased compared to vehicle controls (P_TxT_=0.009; P_TxT* IR_ =0.28; Fig. 3C & D).

### ML355 reduces renal ER stress and oxidative stress in males 7 days post-IR

**Fig. 4:**
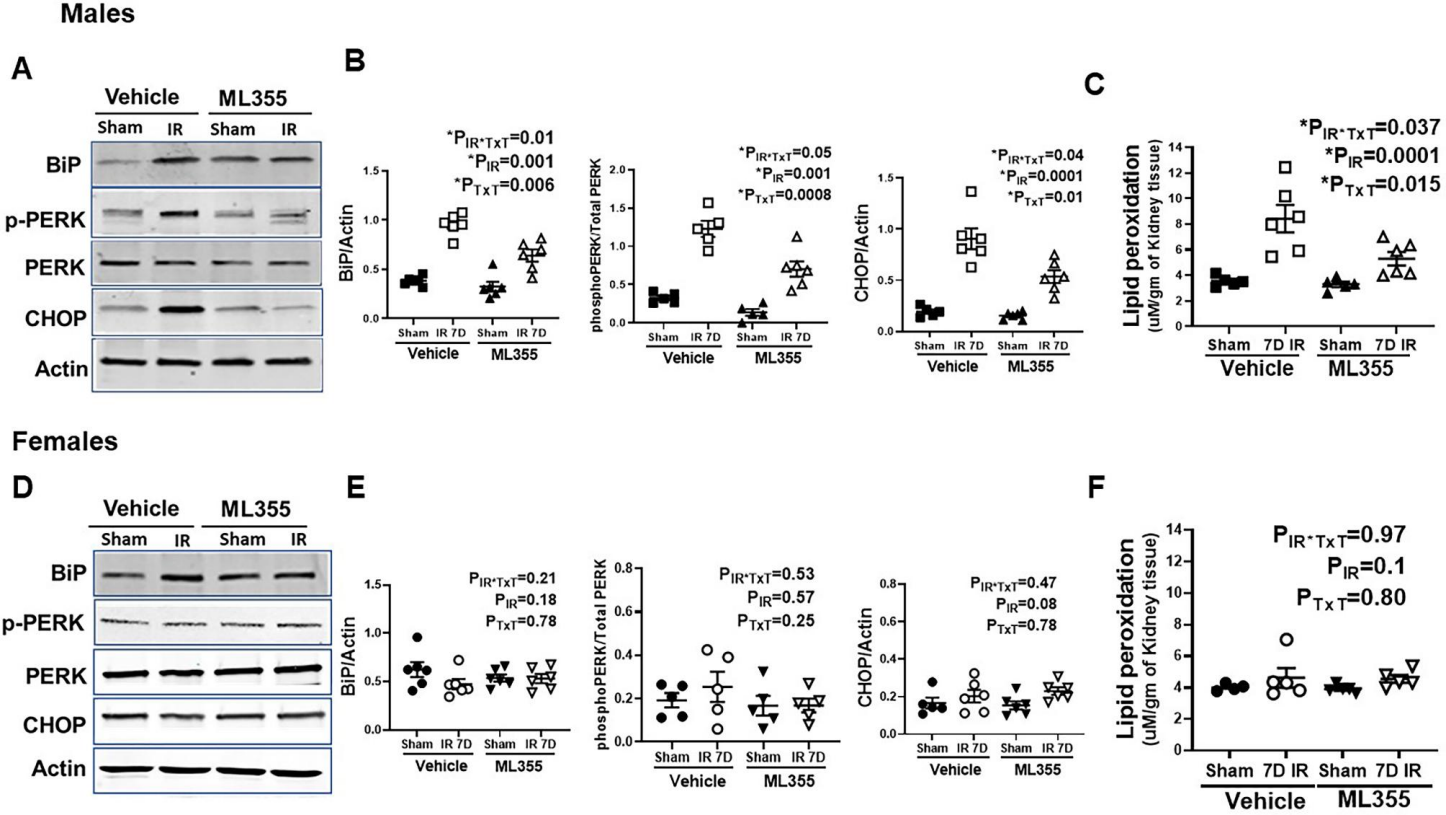
ER stress and oxidative stress were reduced with ML355 treatment in male SHR 7 days post-IR. Renal ER stress markers were assessed by Western blot analysis of in kidney homogenates 7 days post-IR in 13-week-old male and female SHR treated with vehicle or 12/15 LOX inhibitor ML355 1 h prior to sham or 30 min ischemia and every other day up to 7 days post-IR. Panels **A** & **D** are representative Western blots and panels **B** & **E** are the average densitometric analysis of ER stress markers in male and females respectively. Glyceraldehyde 3-phosphate dehydrogenase (GAPDH) expression was used for normalization of protein loading. Renal lipid peroxidation was measured in sham/IR male (Panel **C**) and female SHR (Panel **F**) treated with vehicle or ML355 using thiobarbituric acid reactive substances (TBARS) assay kit. Data are expressed as mean ± SEM, with individual animal data indicated by the symbols, n = 5–6 rats in each group. Filled symbols indicate vehicle or ML355 treated sham animals, open symbols indicate vehicle or ML355-treated IR animals, males are represented by squares and upward triangle and females by circles and downward triangle. Data were compared via 2-way ANOVA with *P* < 0.05 considered significant

Several studies have demonstrated that renal ischemia induces ER stress and oxidative stress in renal tubular cells, which contribute to renal injury (Montie et al. 2005; Yan et al. 2018; Inagi 2010). Importantly, LOX and its metabolite 12/15 HETE are known to induce ER stress and oxidative stress (Cole et al. 2012; Elmasry et al. 2018; Zheng et al. 2020), and both exhibit sustained increases in male SHR post-IR. To determine the contribution of 12/15 LOX on IR-induced increases in ER stress and oxidative stress, indices of both were measured in kidneys of vehicle and ML355-treated male and female rats. Males treated with ML355 had significantly lower expression of ER stress markers (BiP: P_IR_= 0.001; P_TxT_=0.006; P_TxT*IR_=0.01; PERK: P_IR_= 0.001; P_TxT_=0.0008; P_TxT*IR_=0.05; CHOP: P_IR_= 0.0001; P_TxT_=0.01; P_TxT*IR_=0.04) and lipid peroxidation (P_IR_= 0.0001; P_TxT_=0.015; P_TxT*IR_=0.037) 7 days post-IR than vehicle-treated males, although both ER stress markers and lipid peroxidation remain higher than sham controls (Fig. 4A-C). There were no significant differences in ER stress markers (BiP: P_IR_= 0.18; P_TxT_=0.78; P_TxT*IR_=0.21; PERK: P_IR_= 0.57; P_TxT_=0.25; P_TxT*IR_=0.53; CHOP: P_IR_= 0.08; P_TxT_=0.78; P_TxT*IR_=0.47) or lipid peroxidation (P_IR_= 0. 1; P_TxT_=0.80; P_TxT*IR_=0.97) between vehicle and ML355 treated female rats 7 days post-IR (Fig. 4D-F).

### ML355 treatment attenuates increases in cell death and inflammation in males 7 days post-IR

**Fig. 5:**
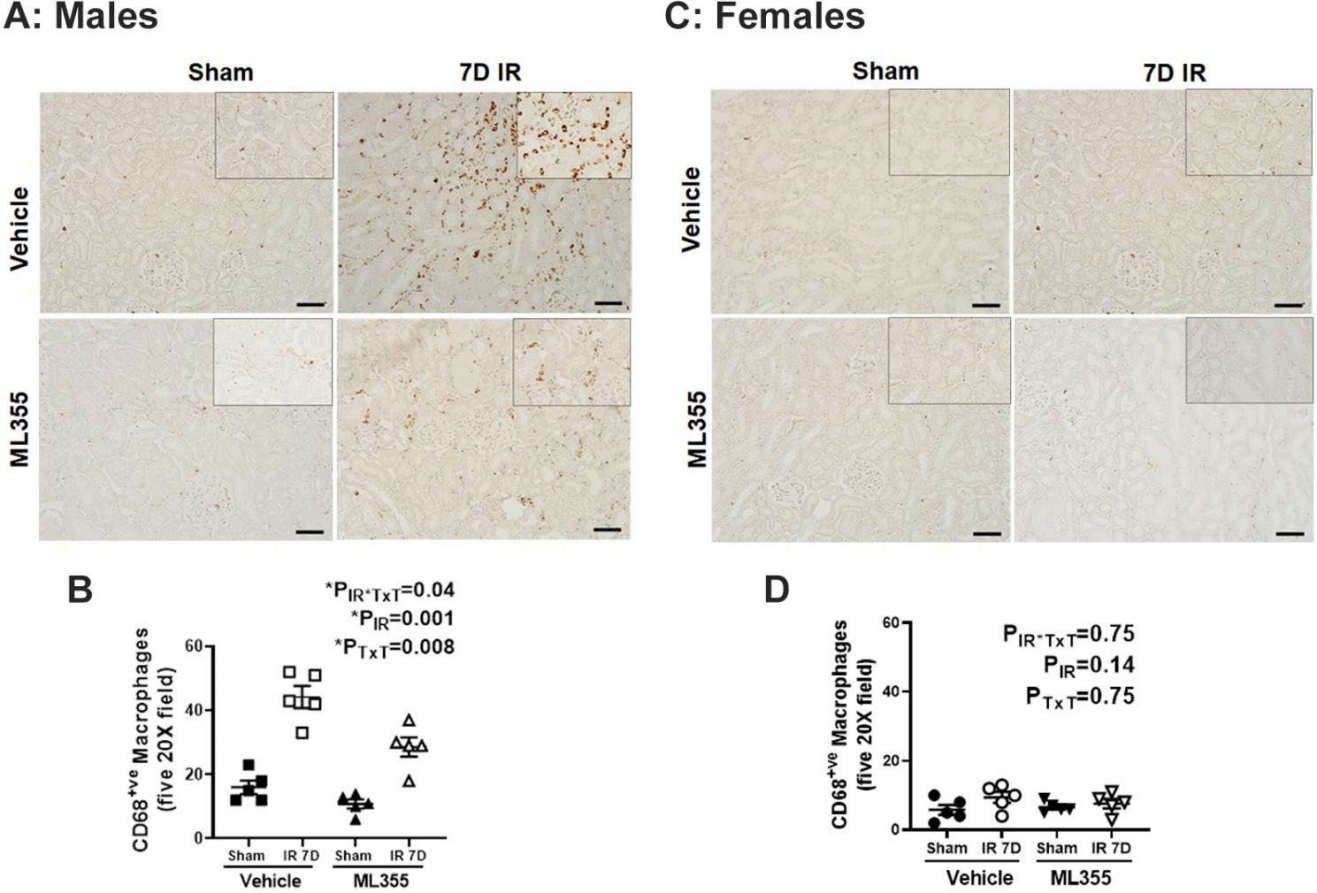
ML355 attenuates IR induced sustained renal cell death 7 days post-IR in males. Renal cell death was measured by TdT-mediated dUTP nick end labeling (TUNEL) staining in kidneys collected 7 days post-IR in 13-week-old male and female SHR treated with treated with vehicle or 12/15 LOX inhibitor ML355 1 h prior to sham/ischemia and every other day up to 7 days post-IR. Brown nuclei with black arrow indicating TUNEL-positive cells. Representative images are shown in Panel **A** for males and Panel C for females with mean quantification data in Panels **B** & **D**. Data are expressed as mean ± SEM, n = 5–6 rats in each group with individual animal data indicated by the symbols. Filled symbols indicate vehicle or ML355 treated sham animals, open symbols indicate vehicle or ML355-treated IR animals, males are represented by squares and upward triangle and females by circles and downward triangle. Data were compared via 2-way ANOVA with *P* < 0.05 considered significant

Renal cell death has been implicated in the pathophysiological and clinical characteristics of ischemic AKI (Linkermann et al. 2014; Inagi 2009). We previously reported that renal cell death remains elevated in male, but not female SHR 7 days post-IR (Mohamed et al. 2022). To determine the contribution of activated 12/15 LOX to cell death in male and female SHR, renal cell death was measured using TUNEL staining in kidneys of vehicle or ML355 treated SHR 7 days following sham or IR surgery. Negligible TUNEL positive nuclei were observed in sham operated kidney sections regardless of treatment in either sex (Fig. 5). Treatment with ML355 reduced TUNEL positive cells in males compared to vehicle treated IR males (P_IR_= 0.0001; P_TxT_=0.006; P_TxT*IR_=0.06), but cell death remained higher than in sham controls (Fig. 5A&B). Females had minimal TUNEL positive cells 7 days post-IR, although levels were reduced to sham levels with ML355 treatment (P_IR_= 0.014; P_TxT_=0.006; P_TxT*IR_=0.25; Fig. 5C&D).

**Fig. 6:**
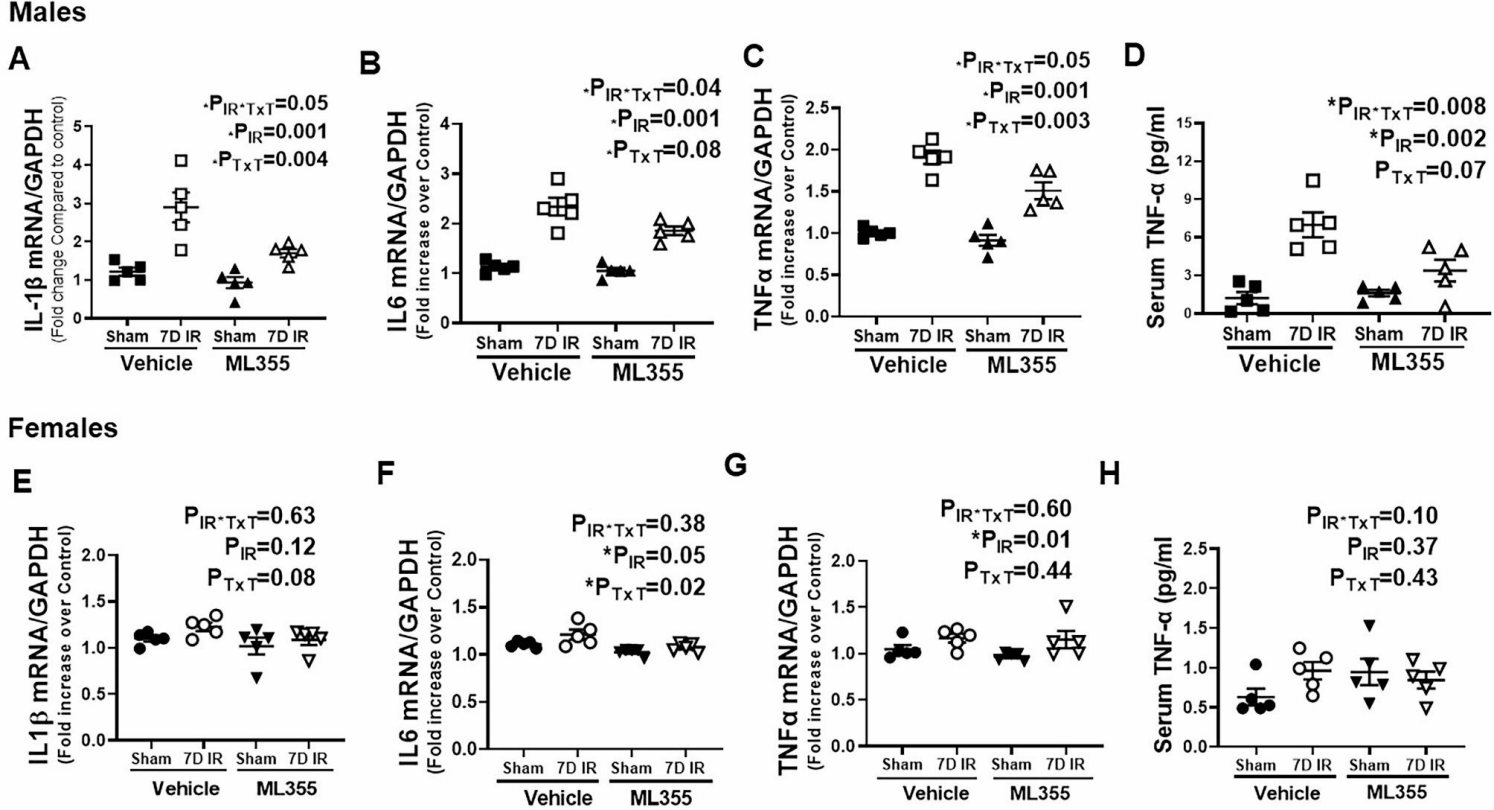
ML355 treatment attenuates IR-induced increases in inflammatory cytokines 7 days post-IR in male SHR. Serum and kidney samples were collected 7 days post-IR in 13-week-old male and female SHR treated with vehicle or 12/15 LOX inhibitor ML355 1 h prior to sham or 30 min ischemia and every other day up to 7 days post-IR. Renal IL-1β mRNA, TNF-α and IL-6 mRNA expression were measured by RT-PCR (Panels **A**-**C** for males and Panels **E**-**G** for females) and serum TNF-α was quantified by ELISA (Panels **D** & **H**). Data are expressed as mean ± SEM; n = 5 rats in each group with individual animal data indicated by the symbols. Filled symbols indicate vehicle or ML355 treated sham animals, open symbols indicate vehicle or ML355-treated IR animals, males are represented by squares and upward triangle and females by circles and downward triangle. Data were compared via 2-way ANOVA with *P* < 0.05 considered significant

**Fig. 7:**
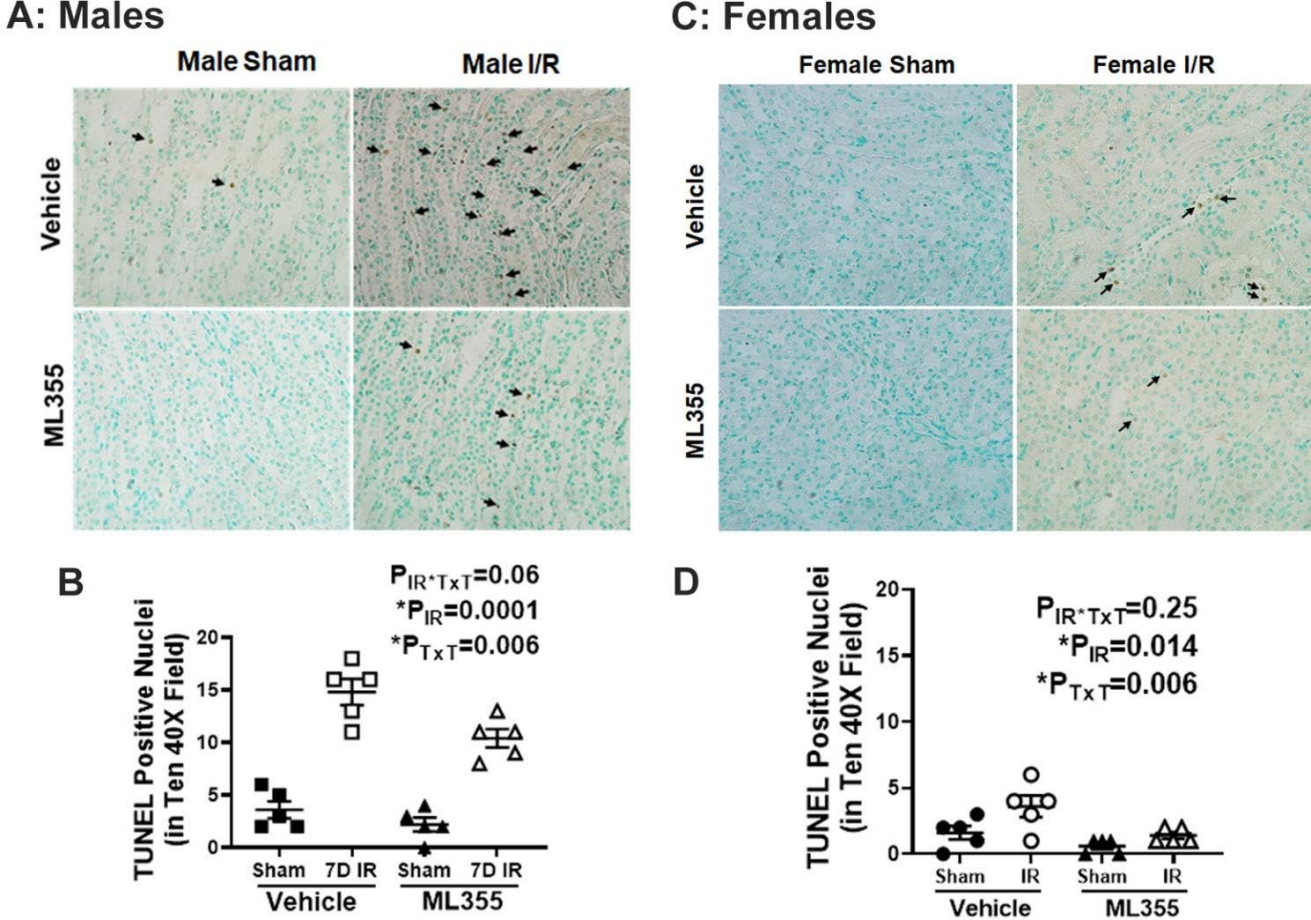
Blocking 12/15 LOX activation attenuates macrophage infiltration 7 days post-IR in male SHR. Renal macrophage infiltration was assessed by immunohistochemical analysis of CD68 staining in kidney samples collected 7 days post-IR in a blinded manner. Brown nuclei staining indicates macrophage CD68 positive cells. Representative images are provided in Panel **A** for males and Panel **C** for females with the respective quantification in Panels **B** & **D**. Data are expressed as mean ± SEM; n = 5–6 rats in each group with individual animal data indicated by the symbols. Filled symbols indicate vehicle or ML355 treated sham animals, open symbols indicate vehicle or ML355-treated IR animals, males are represented by squares and upward triangle and females by circles and downward triangle. Data were compared via 2-way ANOVA with *P* < 0.05 considered significant

Since 12/15 LOX is also known to regulate inflammation (Mohamed et al. 2020), we determined the contribution of 12/15 LOX on renal inflammation in ML355 and vehicle treated male and female SHR 7 days following sham or IR surgery. Renal mRNA expression of IL-1β (P_IR_=0.001), IL-6 (P_IR_=0.001), and TNF-α (P_IR_=0.001), as well as plasma TNF-α levels (P_IR_=0.002) were higher in male SHR 7 days post-IR compared to sham control. Treatment with ML355 resulted in a reduction in circulating and renal inflammatory cytokines 7 days post-IR in males (IL-1β: P_TxT_=0.004; P_TxT*IR_=0.05: IL-6: P_TxT_=0.08; P_TxT*IR_=0.04: TNF-α:P_TxT_=0.003; P_TxT*IR_=0.05 and circulating TNF-α: P_TxT_=0.07; P_TxT*IR_=0.008: Fig. 6A-D). In contrast, among females the levels of IL-1β (P_IR_=0.12: P_TxT_=0.08; P_TxT*IR_=0.63) and circulating TNF-α (P_IR_=0.37; P_TxT_=0.43; P_TxT*IR_=0.10) were largely comparable among all groups 7 days post sham or IR surgery regardless of treatment. However, renal TNF- α and IL-6 mRNA expression were still elevated 7 days post-IR in females (P_IR_=0.05 and P_IR_=0.01, respectively; Fig. 6E-H). Treatment with ML355 prevented sustained increases in renal IL-6 (P_TxT_=0.02; P_TxT*IR_=0.38) with no effect on TNF-α (P_TxT_=0.44; P_TxT*IR_=0.60). To determine if attenuation in sustained inflammatory cytokine expression with 12/15 LOX inhibition was associated with reductions in inflammatory cell infiltration, renal macrophage infiltration was measured using CD68 immunostaining. Consistent with changes in cytokine expression, CD68 positive staining was greater in the male kidney 7 days post-IR vs. sham control (P_IR_=0.001), and treatment with ML355 attenuated macrophage infiltration (P_TxT_=0.008; P_TxT*IR_=0.04: Fig. 7A & B). There were few CD68 positive macrophages in females 7 days post sham or IR surgery regardless of treatment (P_IR_=0.14; P_TxT_=0.75; P_TxT*IR_=0.75: Fig. 7C & D).

### 12/15 LOX inhibition did not alter blood pressure in male or female rats

12/15 LOX enzymes and metabolites are upregulated in both humans with essential hypertension and in various animal models of hypertension (Singh and Rao 2019; Kriska et al. 2012). In addition, inhibition of 12/15 LOX, ER stress or inflammation have also been shown to ameliorate hypertension in male rodents (Dobrian et al. 2011; Kriska et al. 2012; Naiel et al. 2019). To determine if 12/15 LOX inhibition and improved renal recovery was secondary to changes in BP, systolic BP was measured via tail-cuff in vehicle- and ML355-treated male and female SHR at baseline and 7 days post-IR. Males retained a higher systolic BP compared to female SHR throughout the experiment (*P* = 0.05). ML355 treatment did not altered BP in either sex (*P* = 0.32) (Table 1).

**Table 1:** Systolic blood pressure (BP) in male and female spontaneously hypertensive rats (SHR) following sham or renal IR and IR + ML355 treatment

Renal IR	Male SHR			Female SHR		
	Sham	IR	IR + ML355	Sham	IR	IR + ML355
Baseline	189.15 ± 12*	187.8 ± 13*	185.6 ± 13*	165.29 ± 9	164.5 ± 9	168 ± 10
3 day	196.43 ± 10*	190.1 ± 7*	190.8 ± 10*	165.2 ± 7	167.6 ± 8	166.5 ± 10
7 day	187.62 ± 11*	191.2 ± 12*	194.5 ± 08*	162.6 ± 12	165.7 ± 3	167.5 ± 5

Systolic BP was measured via tail-cuff at different time points. *Indicates *p* < 0.05 compared to female counterpart at each time point

## Discussion

The pathogenesis of AKI is complex and multifactorial in nature. Inflammation, tubular injury, tubular epithelial cell apoptosis, and impaired perfusion are prominent features that promote acute tubular necrosis and subsequent progression to renal failure (Hoste et al. 2015; Zhao et al. 9900). AKI is an independent risk factor for CKD progression and mortality (Kellum et al. 2017). Male sex is considered a risk factor for greater incidence and severity of AKI and AKI to CKD progression (Lima-Posada et al. 2017; Hosszu et al. 2020; Neugarten and Golestaneh 2018). We previously reported that male SHR have impaired recovery following renal IR injury compared to females (Mohamed et al. 2022). However, the mechanisms mediating sex differences in IR-recovery are not well known. 12/15 LOX plays a major role in the pathogenesis of a variety of human diseases, including cardiovascular, renal, neurological, and metabolic disorders (Singh and Rao 2019). Consistent with these reports, the main finding of the current study is that sustained activation of 12/15 LOX contributes to delayed post-IR renal recovery in males. We have demonstrated that renal 12/15 LOX activity increased in both male and female SHR 1-day post-IR, although with greater increase in males compared to females. However, 12/15 LOX activity was sustained only in males, along with increased renal ER stress, oxidative stress, and inflammation in parallel to delayed renal recovery compared to females 7 days post-IR. Inhibition of 12/15 LOX reduced renal ER stress, oxidative stress, cell death and inflammation and improved the recovery of renal function in male SHR. It should be noted that ML355 did not alter kidney function and renal 12-HETE level in the females. However, ML355 treatment attenuated IR-induced tubular injury and cell death in females 7 days post-IR. These findings underscore the importance of 12/15 LOX activation in long-term recovery from renal IR in males and females.

12/15 LOX enzymes are a family of iron-containing enzymes that oxidize polyunsaturated fatty acids producing active lipid metabolites that are involved in a plethora of human diseases (Kuhn et al. 2015; Singh and Rao 2019). 12/15 LOX and its metabolites are upregulated in many kidney diseases, including AKI, and are key regulators of renal function (Montford et al. 2022; Sharma et al. 2022; Faulkner et al. 2015; Elmarakby et al. 2019). We report for the first time, that 12/15 LOX activity is also upregulated in females, although to a lesser degree than in males 1 day following renal IR. Importantly, 12/15 LOX activity remained elevated only in males 7 days post-IR, corresponding to delayed recovery of renal function (Mohamed et al. 2022). 12/15 LOX is predominantly expressed in proximal and distal tubular cells (Gonzalez-Nunez et al. 2005), and proximal tubules are the primary target of ischemic injury (Kellum et al. 2017). Consistent with previous reports, immunostaining confirmed that 12/15 LOX protein expression is prominent in injured proximal tubules of male SHR post IR. Moreover, 12/15 LOX and its metabolites 12(S)-HETE and 15(S)-HETE are known to induce ER stress, cell death (Elmasry et al. 2018; Li et al. 2018) and inflammation (Kulkarni et al. 2021; Kar et al. 2020; Faulkner et al. 2015), all of which are commonly observed in the post-ischemic kidney and have been implicated in disease progression (Inagi 2010; Linkermann et al. 2014; Shu et al. 2018). These findings make 12/15 LOX signaling a potential central mechanism controlling ischemic injury and recovery. To directly study the contribution of 12/15 LOX activation on renal recovery, male and female SHR were treated with the specific 12/15 LOX inhibitor ML355 as previously reported (Adili et al. 2017). ML355 treatment reduced IR-induced sustained tubular damage and enhanced recovery of renal function in male SHR. Interestingly, although tubular injury and cell death were less in vehicle treated females compared to males, levels were attenuated byML355 treatment. These data suggest that 12/15 LOX may contribute to sustained tubular damage in both sexes, although males are more susceptible tothis mechanism than females. While not assessed in the current study, delayed recovery in males compared to females may also be related to reduced activity of glutathione peroxidase-4 (GpX4) in the kidney. Glutathione peroxidase-4 (GPx4) neutralizes lipid peroxides produced by 12/15 LOX protect the kidney from injury (Friedmann Angeli et al. 2014), suggesting imbalance of 12/15 LOX and glutathione peroxidase-4 (GpX4) activity may contribute to delayed recovery in males. Future studies will assess the balance of 12/15 LOX and GPx4 in the post-ischemic kidney.

An increasing number of studies support the view that ER stress and oxidative stress contribute to the pathogenesis of ischemic AKI (Shu et al. 2018; Tomsa et al. 2019). In the current study we found that male SHR have greater renal ER stress and oxidative stress compared to females 7 days post-IR. Under pathological conditions, an accumulation of unfolded and misfolded proteins in the ER triggers the unfolded protein response (UPR) or ER stress [45]. ER stress has been implicated in the pathogenesis of AKI induced by renal IR [45, 46]. Moreover, kidneys of male mice are more susceptible to AKI induced ER stress and oxidative stress than those of females (Lima-Posada et al. 2017; Hodeify et al. 2013). However, the mechanisms mediating sex differences in ER stress in the post-ischemic kidney are not well known. Importantly, 12/15 LOX activation is known to induce ER stress and oxidative stress (Cole et al. 2012), and we found that inhibition of 12/15 LOX reduced IR-induced renal ER stress markers (PERK, CHOP) and lipid peroxidation in male SHR. These data support 12/15 LOX as a novel contributing factor for sustained ER stress and oxidative stress in the post ischemic kidney of males.

Understanding the pathways activating cell death can provide new options to limit injury and enhance recovery. Tubular epithelial cell death is commonly observed in ischemic AKI and contributes to the pathophysiology (Linkermann et al. 2014). We previously reported that male SHR exhibit persistent renal cell death following ischemic injury, while cell death resolves more rapidly in the days following ischemic insult in females (Mohamed et al. 2022). However, what mediates persistent cell death in males is still not well understood. 12/15 LOX and its metabolites can induce multiple cell death pathways, including apoptosis, autophagy and ferroptosis (Li et al. 2018), and we observed that inhibition of 12/15 LOX reduced renal cell death (TUNEL positive nuclei), supporting 12/15 LOX activation as a novel contributor for renal cell death following post-ischemic injury in males and females. How 12/15 LOX induces cell death was not examined, however, excessive ER stress results in the persistent activation of unfolded protein response, which can promote cell death (Habshi et al. 2023). Indeed, activation of the pro-apoptotic effector PERK and CHOP induce apoptosis and exacerbate kidney dysfunction in mouse models of IR, toxin-induced AKI, and obstructive nephropathy (Yan et al. 2018; Inagi 2010; Shu et al. 2018). These studies all indicate that ER stress might contribute to tubular cell death. Future studies will determine the contribution of ER stress to 12/15 LOX-induced cell death.

Renal infiltration of monocytes/macrophages and the associated inflammation have also been implicated in the pathogenesis of kidney diseases (Meng et al. 2022; Wang and Harris 2011). Moreover, infiltrating macrophages from circulation and renal resident macrophages accumulate in the injured kidney and augment inflammatory responses and disease progression (Meng et al. 2022). Upregulation of 12/15 LOX and its metabolites exacerbate inflammation contributing to the progression of organ damage (Dobrian et al. 2011; Elmarakby et al. 2019). We found that inhibition of 12/15 LOX significantly reduced the inflammatory response following IR in males and females. suggesting that 12/15 LOX contributes to inflammatory response during post ischemic recovery in males. While the mechanism by which 12/15 LOX induces inflammation was not examined, we previously reported that the release of the damage associated molecular pattern (DAMP) HMGB1 exacerbates inflammatory responses in the male, but not female kidney following IR (Mohamed et al. 2020). Since DAMPs are released following cell death and the post-ischemic kidney of male SHR has sustained renal cell death (Mohamed et al. 2022), this could help explain the sex difference in recovery following an ischemic insult to the kidney. Moreover, previous studies have reported that long-lasting subclinical tubular injury persists following renal ischemia, even after serum creatinine levels return to baseline values (Kellum et al. 2017; Gillis et al. 2021). Therefore, additional analysis of persistent subclinical tubular injury and its impact on renal repair are needed. Future studies will examine the specific contribution of subclinical tubular injury and HMGB1 release to 12/15 LOX mediated impaired post-IR recovery in males and females.

There are limitations to the current study. We identified 12/15 LOX as a key therapeutic target to enhance post-IR recovery in males, the molecular mechanisms mediating these associations were not investigated in the current study. Indeed, 12/15-LOX activation and 12-HETE are all known to induce endoplasmic stress, lipid peroxidation, cell death and inflammation, but additional studies are needed to unravel the relative contribution of each of these on renal recovery. Our work in a rat model of human disease supports the investigation of 12/15 LOX pathways in humans. However, more work is needed as 12/15 LOX is also expressed in other cell types, therefore systematic administration of ML355 may have off target effect. Therefore, chronic safety and systematic influence of ML355 should be investigated before considering clinical application of LOX inhibitor ML355 for ischemic AKI in humans. Lastly, mice and rats are often chosen as pre-clinical animal models to study the basic mechanisms of ischemic AKI and to evaluate potential therapeutics, although, animal models of kidney disease have some disadvantages and limitations in mimicking complex human diseases. Despite this, animal models represent an invaluable tool to understand the mechanism of complex disease and often represent an indispensable approach for drug screening, to predict the effects of a drug, selecting appropriate dose regime, evaluating toxicity that balances efficacy and safety in the complex human system in the preparation for clinical trials.

## Conclusion

The current study identified a novel pathway that contributes to sex differences in IR recovery in male and females. We demonstrated that renal 12/15 LOX activation is sustained in males in parallel to delayed renal recovery from ischemic injury compared to females. Further, inhibition of 12/15 LOX enhanced renal structural and functional recovery in male SHR, indicating that 12/15 LOX activation is major contributing factor for sex differences in renal recovery following IR. These findings highlight the importance of sex specific activation of 12/15 LOX and its contribution to renal repair but also offer a novel therapeutic target to enhance kidney recovery after renal IR and halt AKI to CKD progression in male and females.

### Electronic supplementary material

Below is the link to the electronic supplementary material.

**Supplementary Material 1: Supplementary Figure 1:** Circulating levels of 12/15 LOX metabolite 12/15-HETE increased in males and females 7 days post-IR. 12/15 LOX bioactive metabolite 12/15 HETE was measured in plasma by ELISA in 13-week-old male and female SHR 1 day (Panel A) and 7 days (Panel B) following sham or 30 min bilateral ischemia reperfusion (IR). Data are expressed as mean ± SEM with individual animal data indicated by the symbols, n = 5–6 rats in each group. Filled symbols indicate sham animals, open symbols indicate ischemia, males are represented by squares and females by circles. Data were compared via 2-way ANOVA with *P* < 0.05 considered significant. 
**Supplementary Figure 2**. Renal 15 HETE level is not altered in male and females 7 days post-IR. Amount of the major metabolic product of 12/15 LOX, 15 HETE, was measured in kidney tissues of 13-week-old male and female at 1 day (Panel A) and 7 day (Panel B) following IR by LC/MS. Data are expressed as mean ± SEM with individual animal data indicated by the symbols, n = 5–6. Filled symbols indicate sham animals, open symbols indicate ischemia, males are represented by squares and females by circles. Data were compared via 2-way ANOVA with *P* < 0.05 considered significant

**Supplementary Material 2:** Original western blot gels (unprocessed) and microscopy images associated with manuscript

## Data Availability

All raw data and materials are available upon request from the corresponding author.

## Abbreviations

DMSO: Dimethylsulfoxide
HETE: Hydroxyeicosatetraenoic acids
LC/MS: Liquid chromatography–mass spectrometry
