# Supplementary material for: Sustained activation of 12/15 lipoxygenase (12/15 LOX) contributes to impaired renal recovery post ischemic injury in male SHR compared to females
Source: Mol Med. 2023 Dec 4;29:163. doi: 10.1186/s10020-023-00762-y (PMC10696802; doi:10.1186/s10020-023-00762-y)
Supplement: Supplementary file 1 — Supplementary Material 1: Supplementary Figure 1: Circulating levels of 12/15 LOX metabolite 12/15-HETE increased in males and females 7 days post-IR. 12/15 LOX bioactive metabolite 12/15 HETE was measured in plasma by ELISA in 13-week-old male and female SHR 1 day (Panel A) and 7 days (Panel B) following sham or 30 min bilateral ischemia reperfusion (IR). Data are expressed as mean ± SEM with individual animal data indicated by the symbols, n = 5–6 rats in each group. Filled symbols indicate sham animals, open symbols indicate ischemia, males are represented by squares and females by circles. Data were compared via 2-way ANOVA with P < 0.05 considered significant. Supplementary Figure 2. Renal 15 HETE level is not altered in male and females 7 days post-IR. Amount of the major metabolic product of 12/15 LOX, 15 HETE, was measured in kidney tissues of 13-week-old male and female at 1 day (Panel A) and 7 day (Panel B) following IR by LC/MS. Data are expressed as mean ± SEM with individual animal data indicated by the symbols, n = 5–6. Filled symbols indicate sham animals, open symbols indicate ischemia, males are represented by squares and females by circles. Data were compared via 2-way ANOVA with P < 0.05 considered significant [file 10020_2023_762_MOESM1_ESM.docx]

**Sustained activation of 12/15 Lipoxygenase (12/15 LOX) contributes to impaired renal recovery post ischemic injury in males compared to females.**

Riyaz Mohamed and Jennifer C. Sullivan

Department of Physiology, Medical College of Georgia at Augusta University

Running head: Greater 12/15 LOX activation impairs renal recovery in males

Corresponding Author:

Riyaz Mohamed, PhD

Medical College of Georgia at Augusta University

Department of Physiology

1459 Laney Walker Blvd CB-2204

Augusta, GA 30912

Telephone: 706-721-1741

Fax: 706-721-9799

Email: [rmohamed@augusta.edu](mailto:jensullivan@augusta.edu)


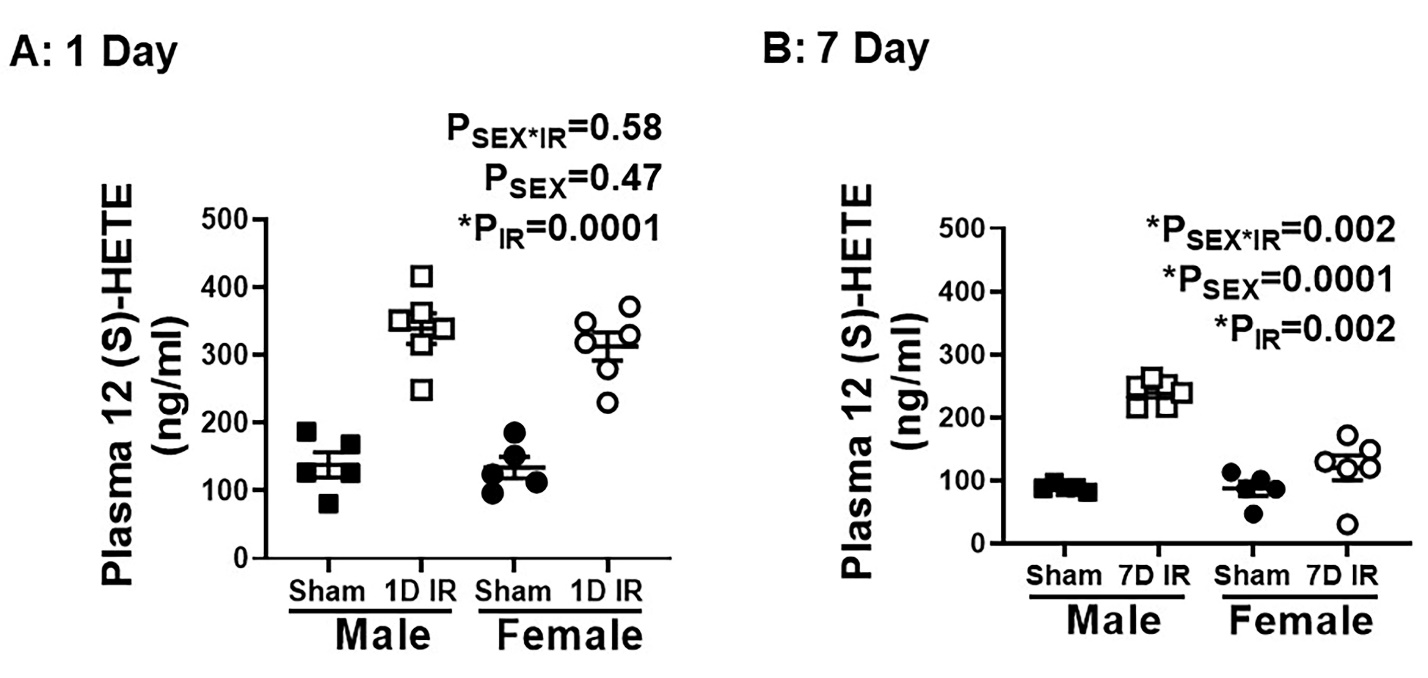


**Supplementary Figure 1: Circulating levels of 12/15 LOX metabolite 12/15-HETE increased in males and females 7 days post-IR.** 12/15 LOX bioactive metabolite 12/15 HETE was measured in plasma by ELISA in 13-week-old male and female SHR 1 day (Panel A) and 7 days (Panel B) following sham or 30 minutes bilateral ischemia reperfusion (IR). Data are expressed as mean ± SEM with individual animal data indicated by the symbols, n=5-6 rats in each group. Filled symbols indicate sham animals, open symbols indicate ischemia, males are represented by squares and females by circles. Data were compared via 2-way ANOVA with P<0.05 considered significant.


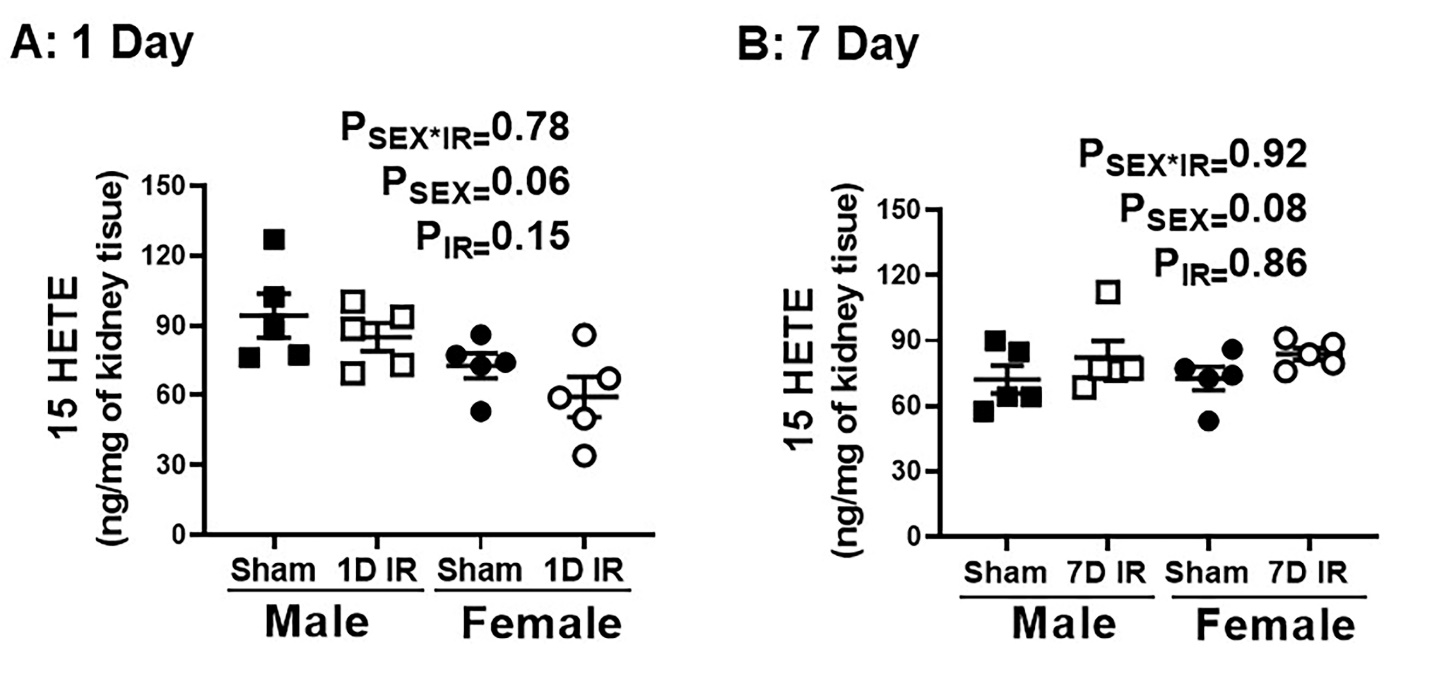


**Supplementary Figure 2. Renal 15 HETE level is not altered in male and females 7 days post-IR.** Amount of the major metabolic product of 12/15 LOX, 15 HETE, was measured in kidney tissues of 13-week-old male and female at 1 day (Panel A) and 7 day (Panel B) following IR by LC/MS. Data are expressed as mean ± SEM with individual animal data indicated by the symbols, n=5-6. Filled symbols indicate sham animals, open symbols indicate ischemia, males are represented by squares and females by circles. Data were compared via 2-way ANOVA with P<0.05 considered significant.
