# Supplementary material for: Sustained activation of 12/15 lipoxygenase (12/15 LOX) contributes to impaired renal recovery post ischemic injury in male SHR compared to females
Source: Mol Med. 2023 Dec 4;29:163. doi: 10.1186/s10020-023-00762-y (PMC10696802; doi:10.1186/s10020-023-00762-y)

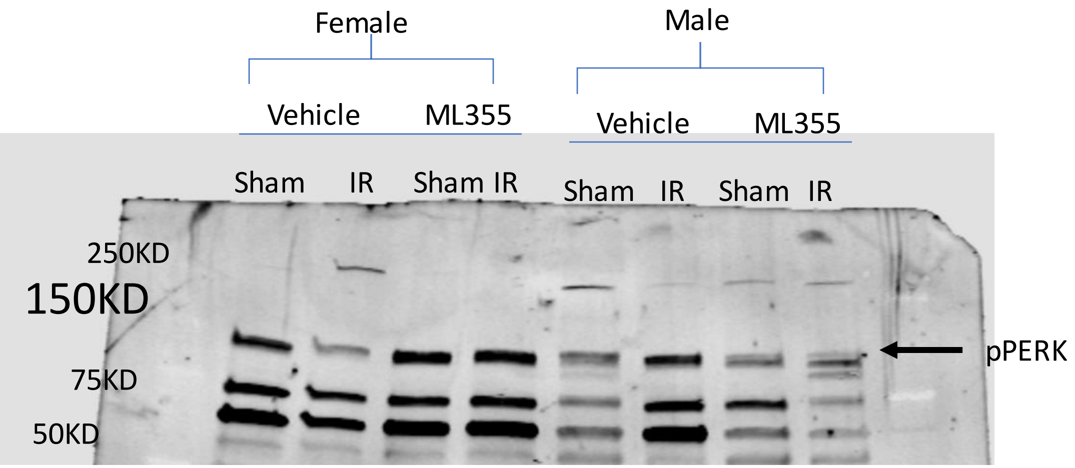
Female
Male
Vehicle
ML355
Vehicle
ML355
Sham
IR
Sham IR
Sham
IR
Sham
IR
250KD
150KD
75KD
50KD
pPERK

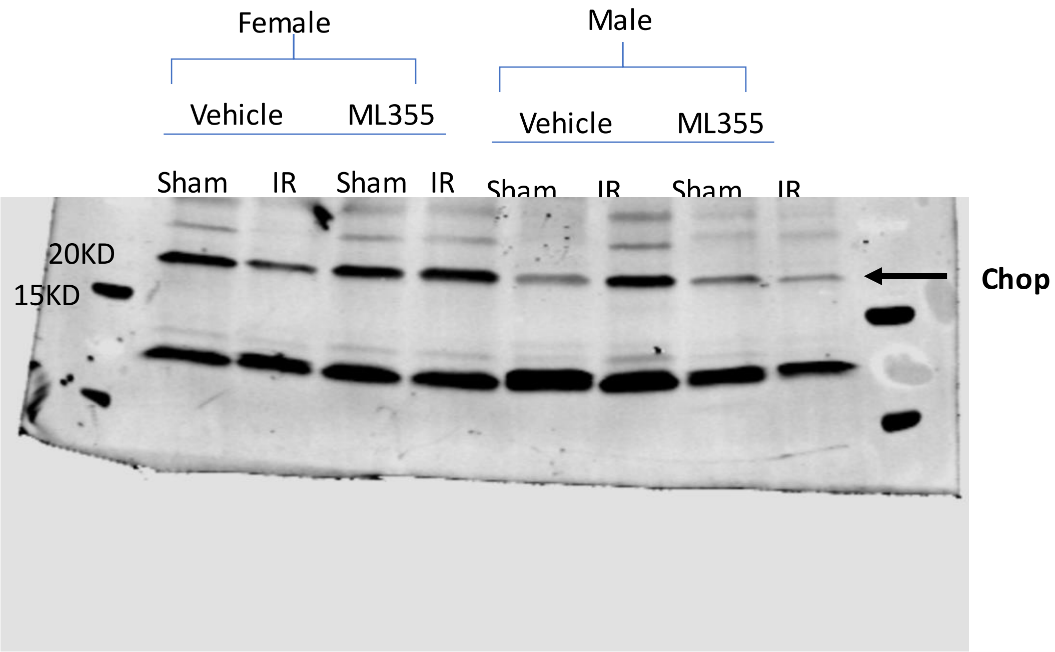
Female
Male
Vehicle
ML355
Vehicle
ML355
Sham
IR
Sham
IR
20KD
15KD
Chop

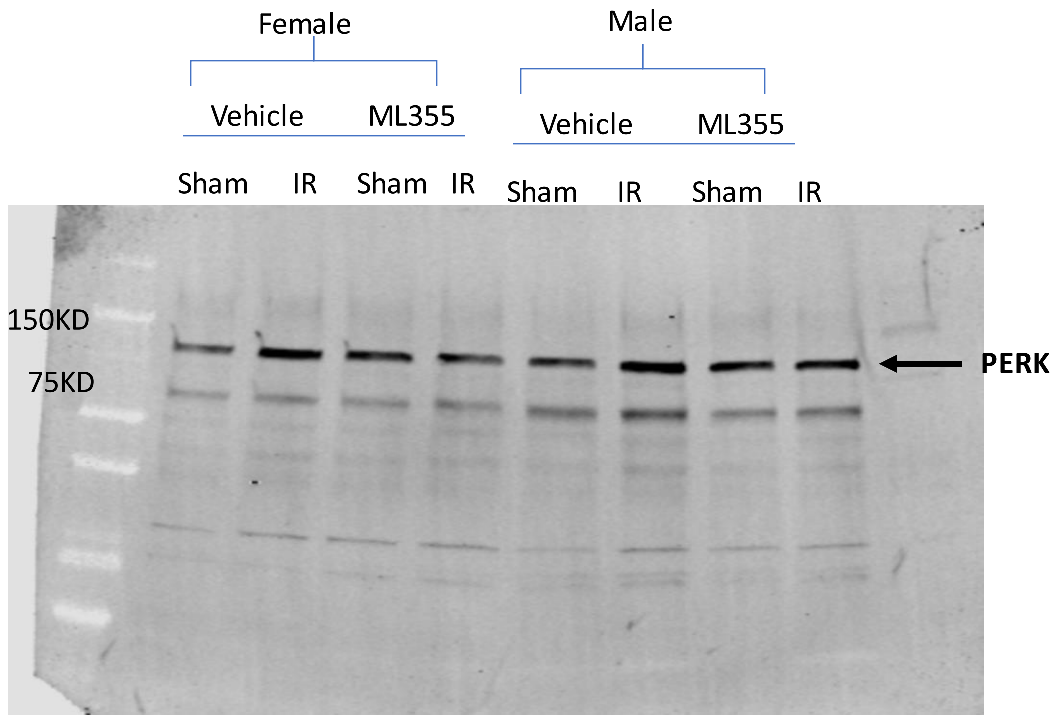
Female
Male
Vehicle
ML355
Vehicle
ML355
Sham
IR
Sham
IR
Sham
IR
Sham
IR
150KD
75KD
PERK

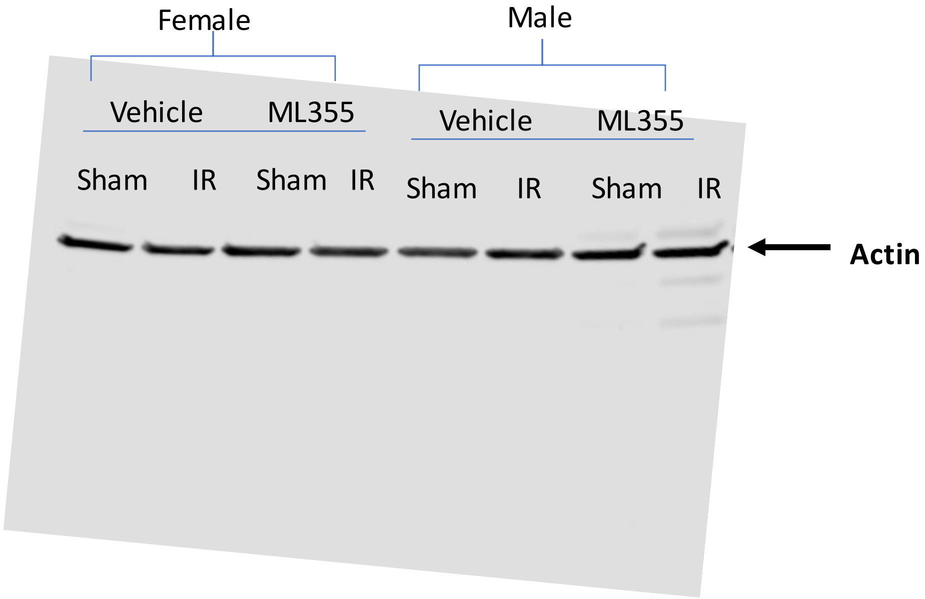
Female
Male
Vehicle
ML355
Vehicle
ML355
Sham
IR
Sham
IR
Sham
IR
Sham
IR
Actin

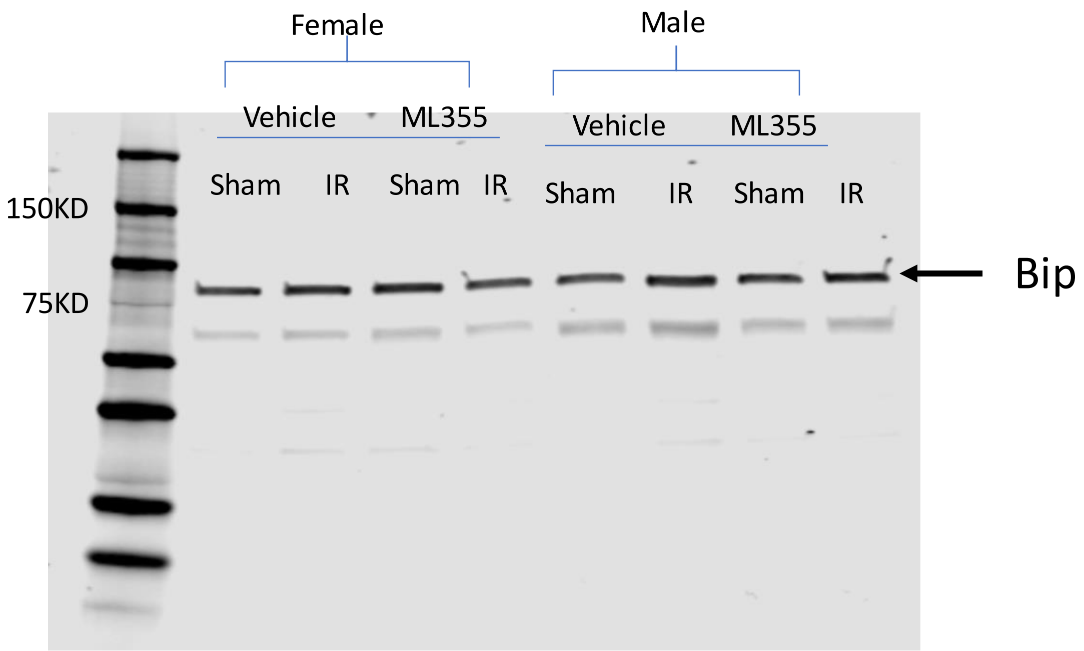
Female
Male
Vehicle
ML355
Vehicle
ML355
Sham
IR
Sham
IR
Sham
IR
Sham
IR
150KD
75KD
Bip

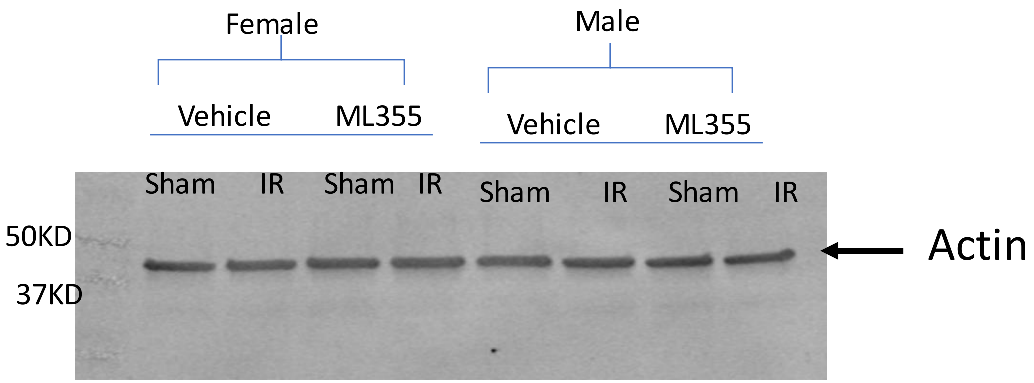
Female
Male
Vehicle
ML355
Vehicle
ML355
Sham
IR
Sham
IR
Sham
IR
Sham
IR
50KD
37KD
Actin

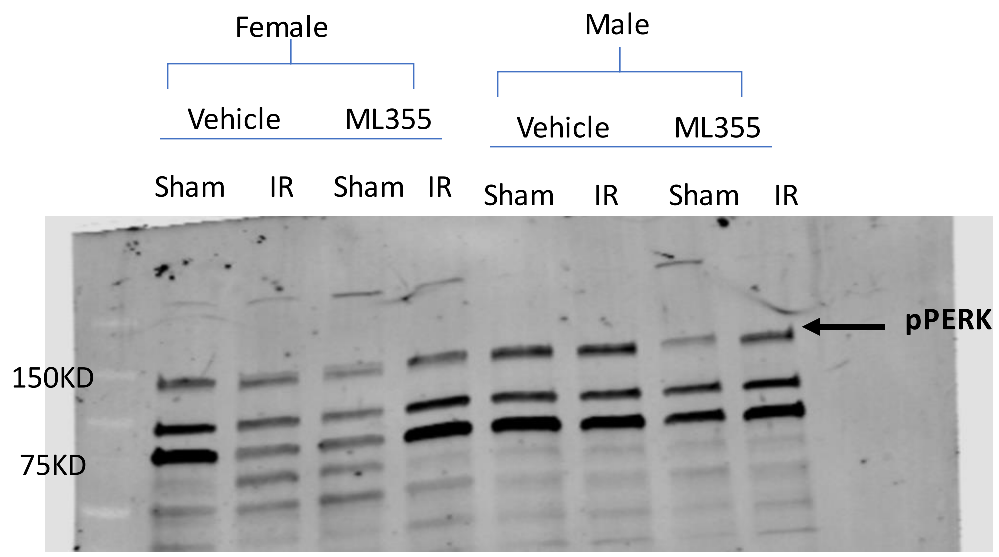
Female
Male
Vehicle
ML355
Vehicle
ML355
Sham
IR
Sham
IR
Sham
IR
Sham
IR
pPERK
150KD
75KD

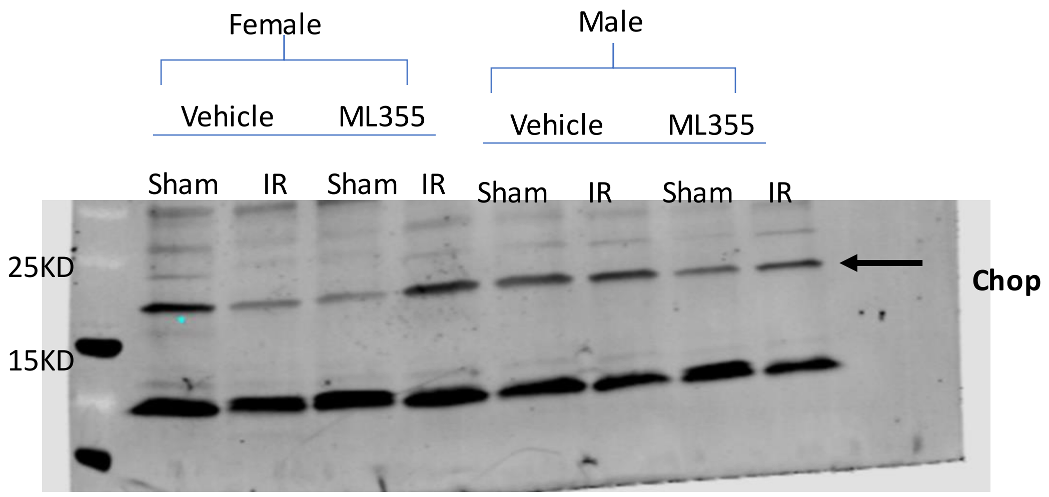
Female
Male
Vehicle
ML355
Vehicle
ML355
Sham
IR
Sham
IR
Sham
IR
Sham
IR
Chop
25KD
15KD

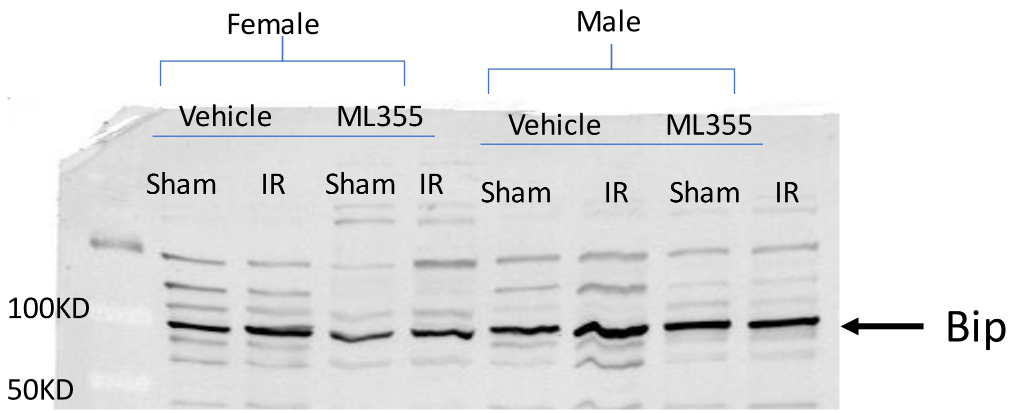
Female
Male
Vehicle
ML355
Vehicle
ML355
Sham
IR
Sham
IR
Sham
IR
Sham
IR
100KD
50KD
Bip

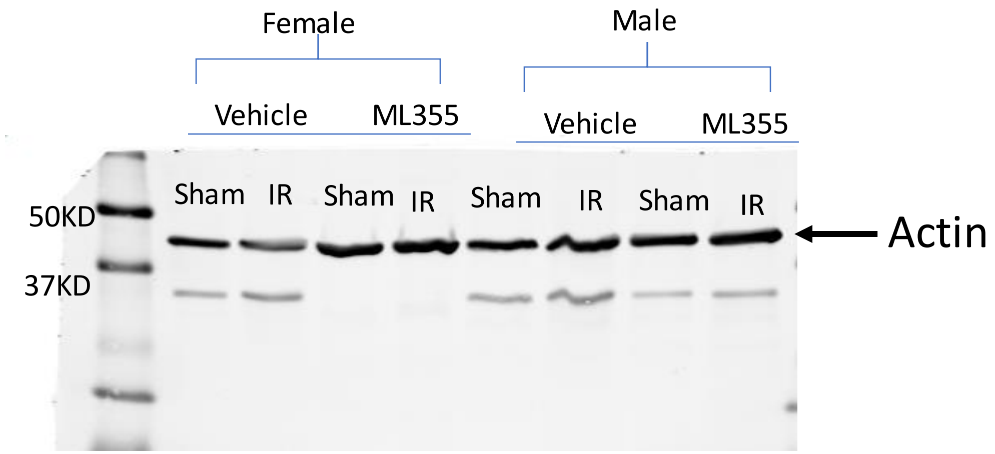
Female
Male
Vehicle
ML355
Vehicle
ML355
Sham
IR
Sham
IR
Sham
IR
Sham
IR
50KD
37KD
Actin

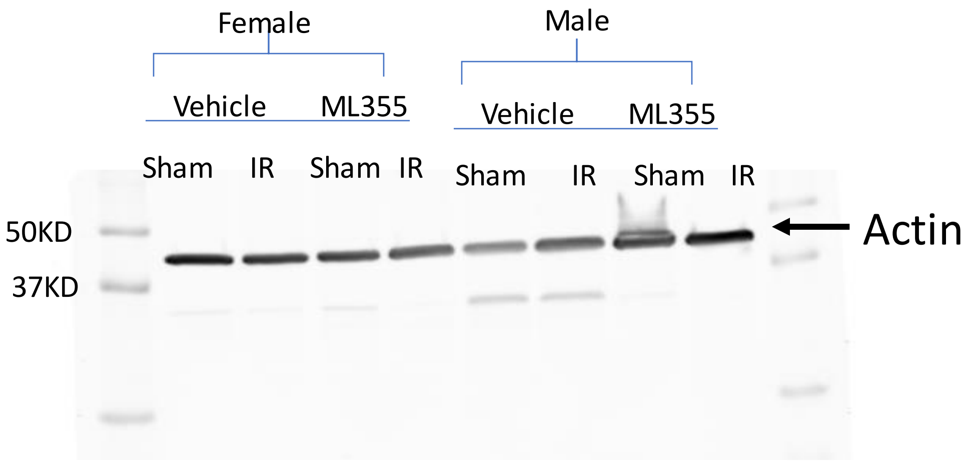
Female
Male
Vehicle
ML355
Vehicle
ML355
Sham
IR
Sham
IR
Sham
IR
Sham
IR
50KD
37KD
Actin

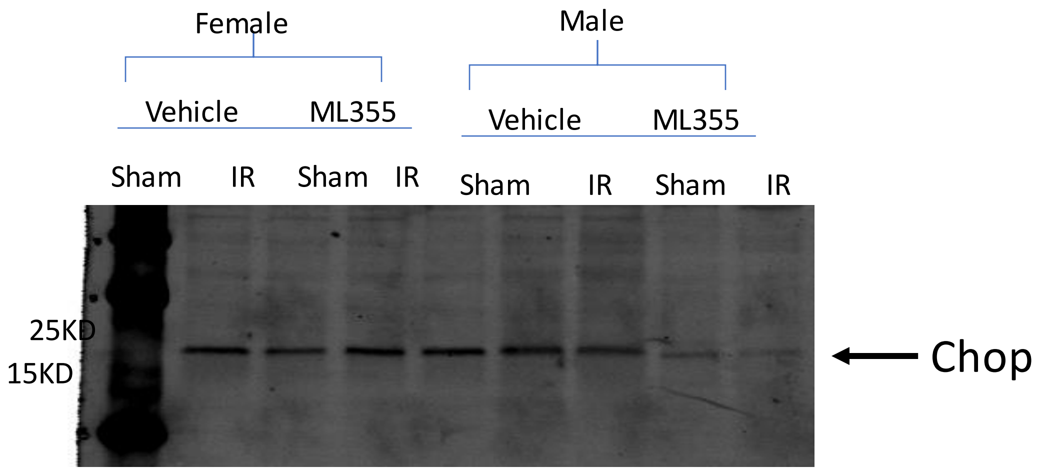
Female
Male
Vehicle
ML355
Vehicle
ML355
Sham
IR
Sham
IR
Sham
IR
Sham
IR
25KD
15KD
Chop

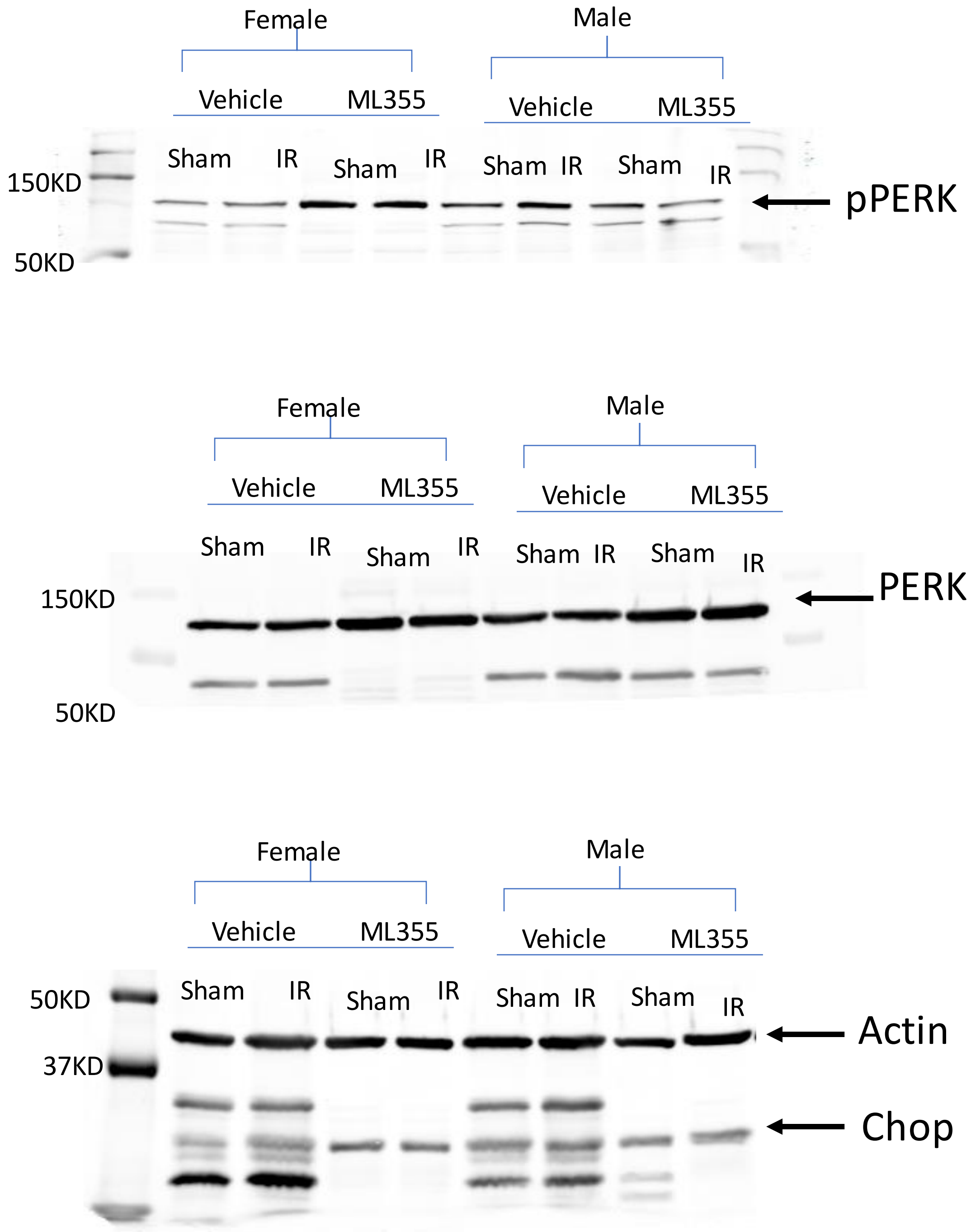
Female
Male
Vehicle
ML355
Sham
IR
150KD
50KD
pPERK
PERK
37KD
Actin
Chop

Male Sham

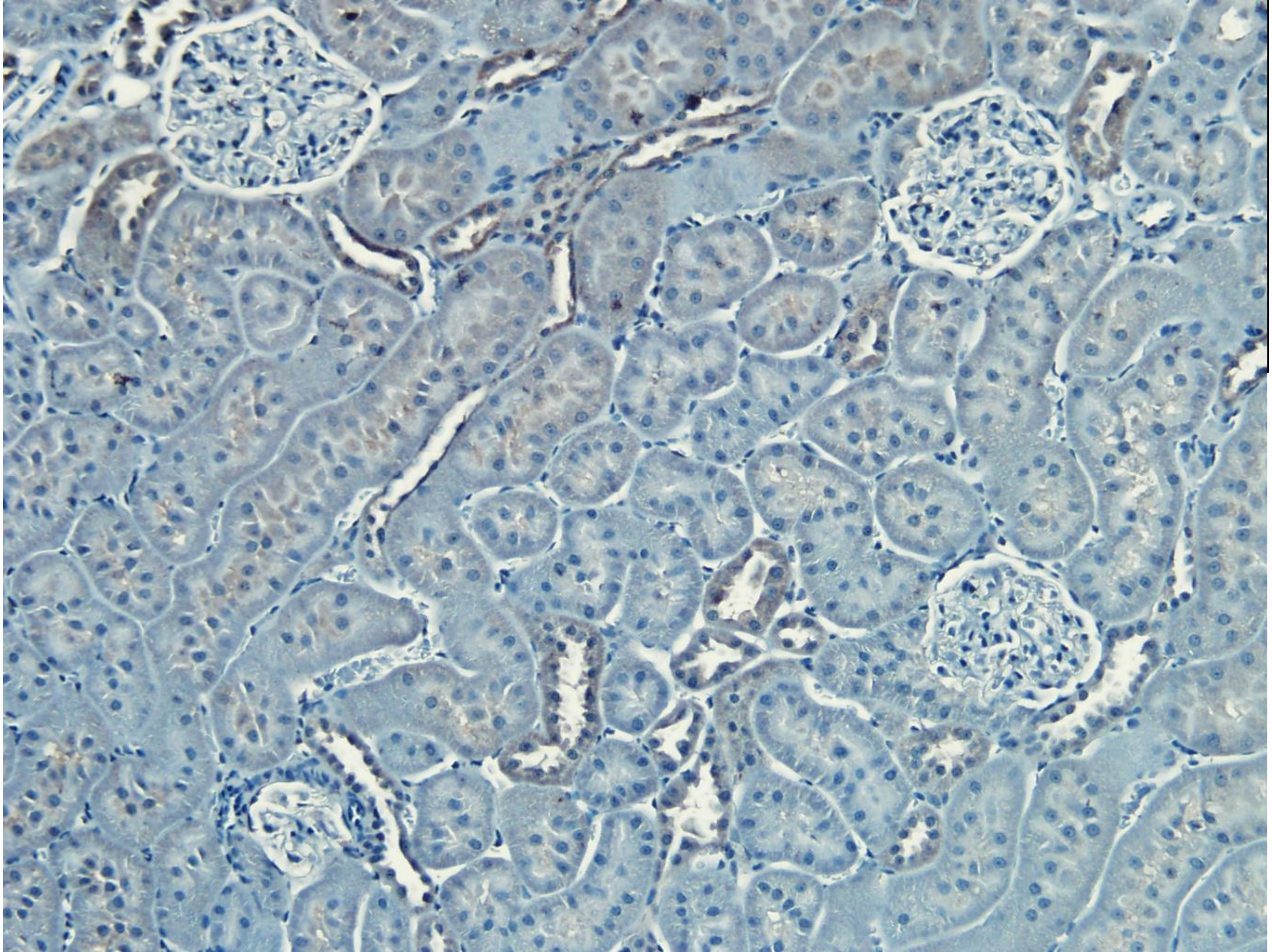

Female Sham

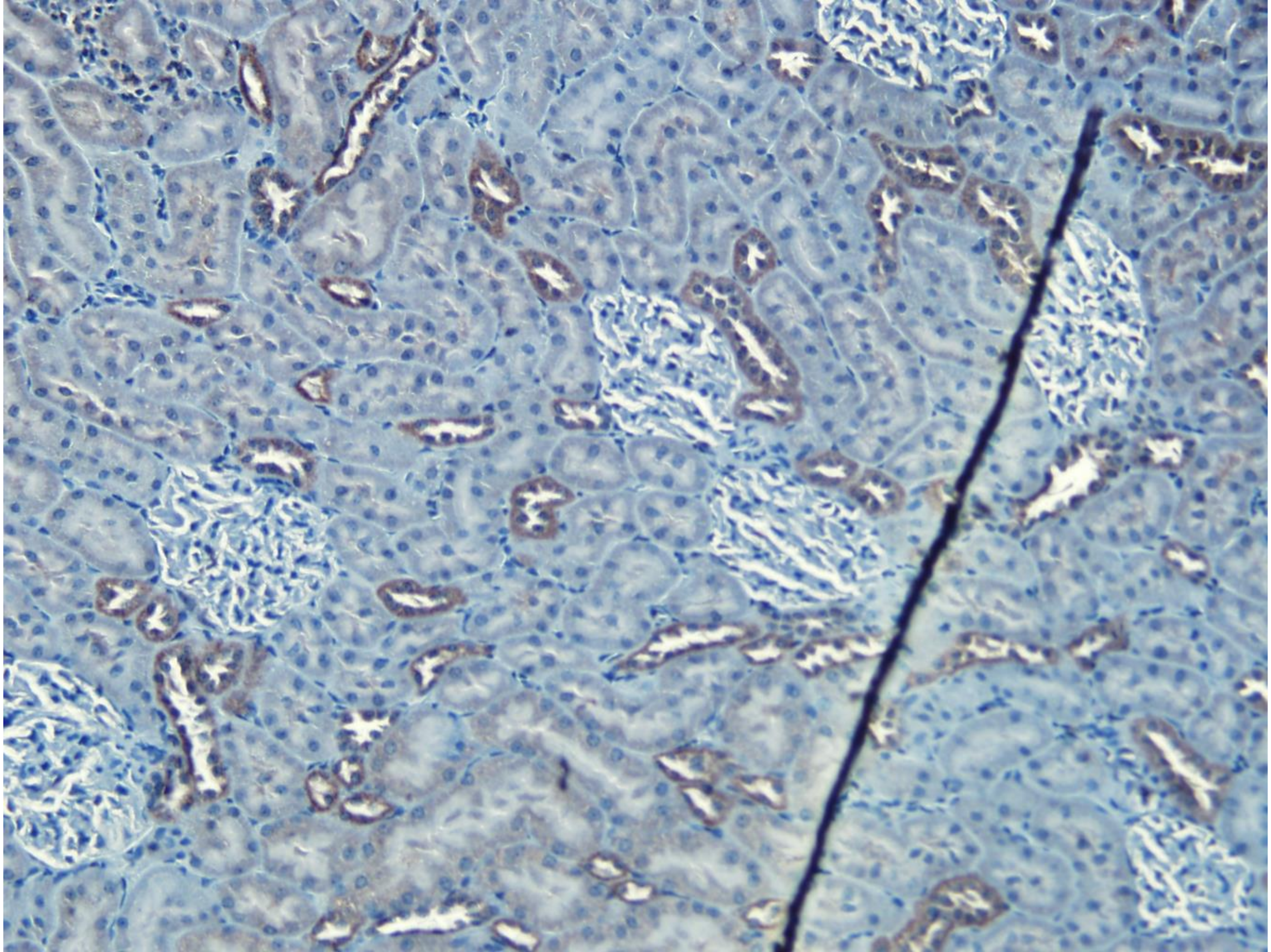

Male 1D I/R

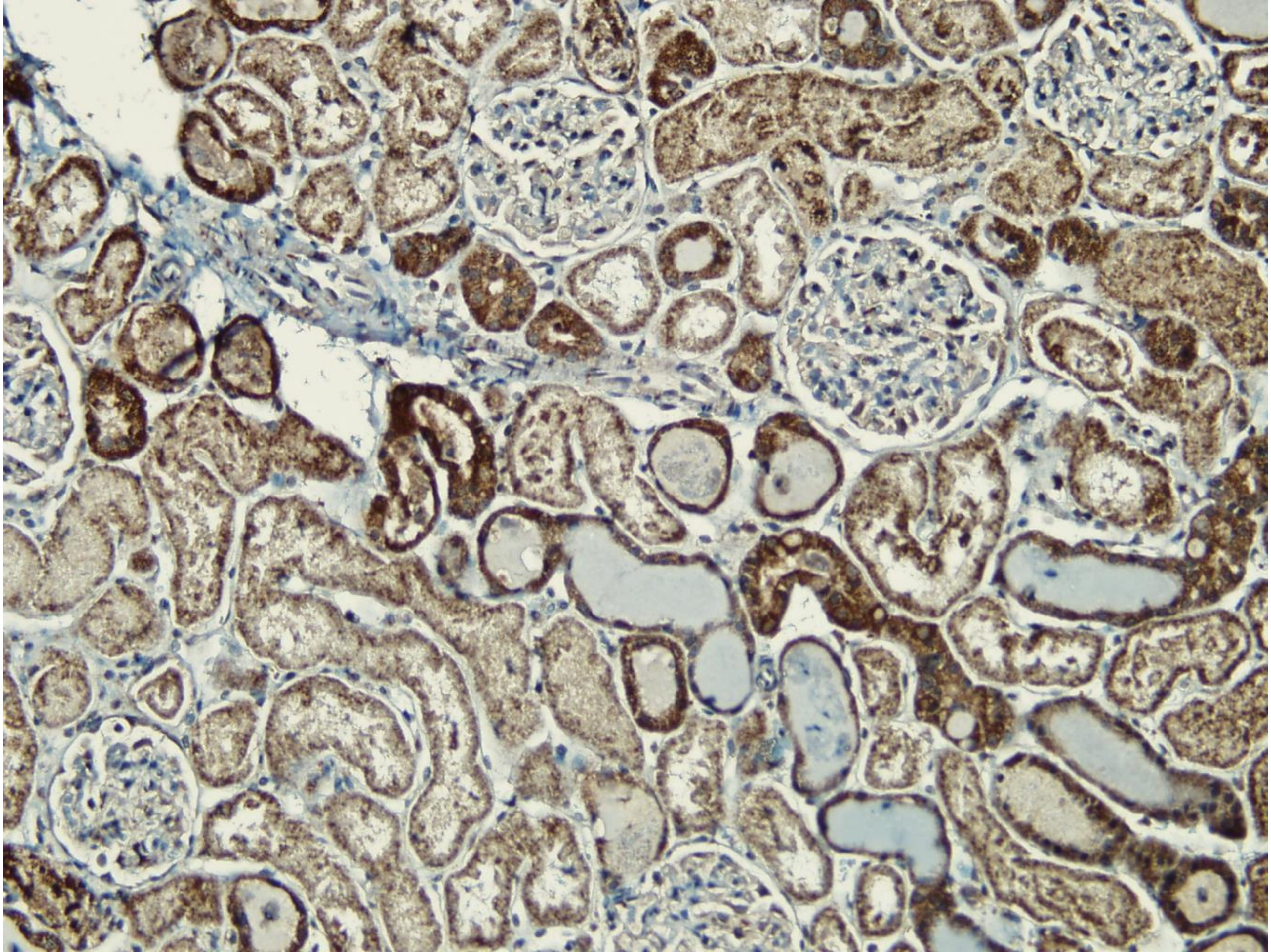

Female 1D I/R

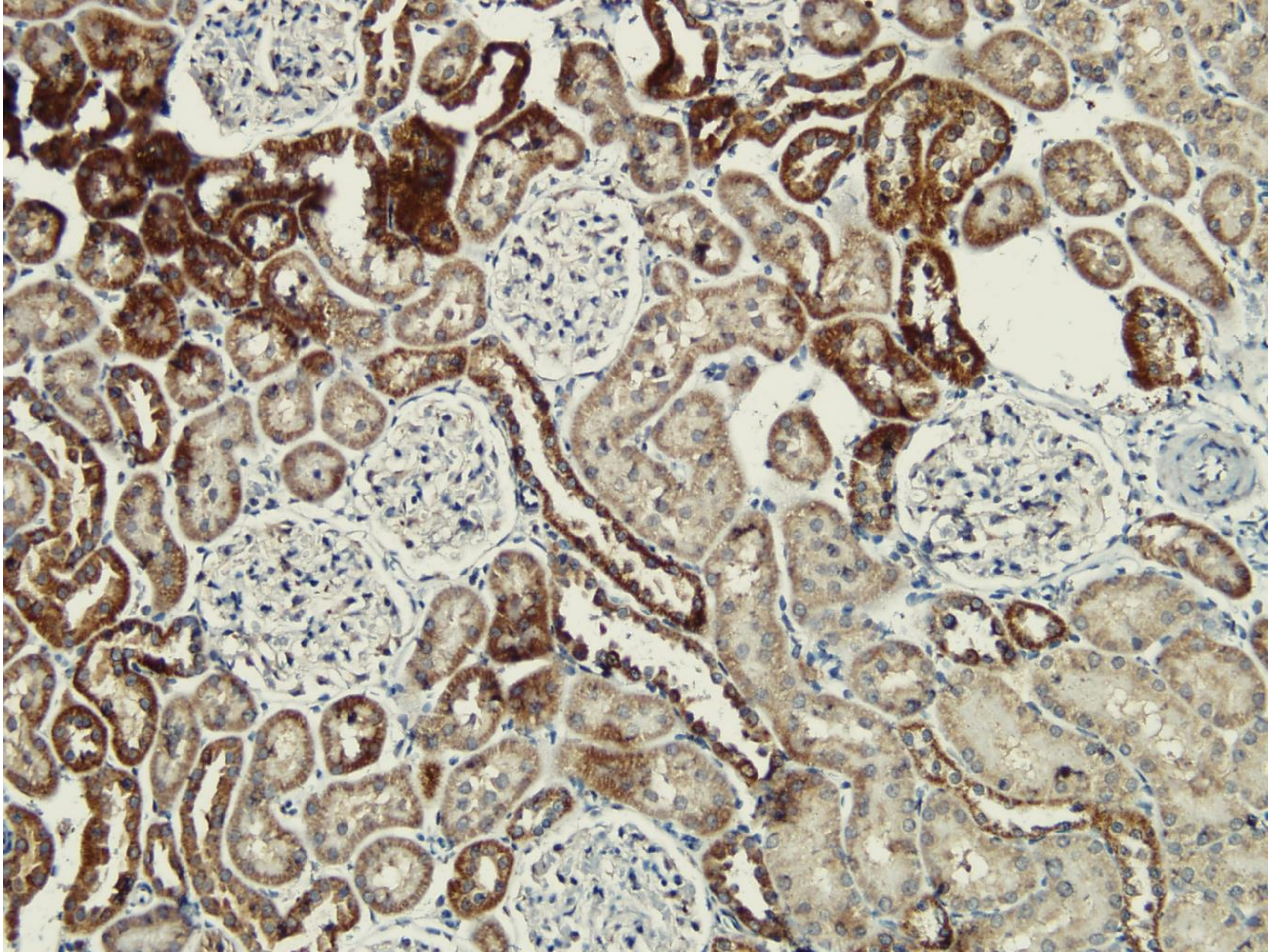

Male 7D I/R

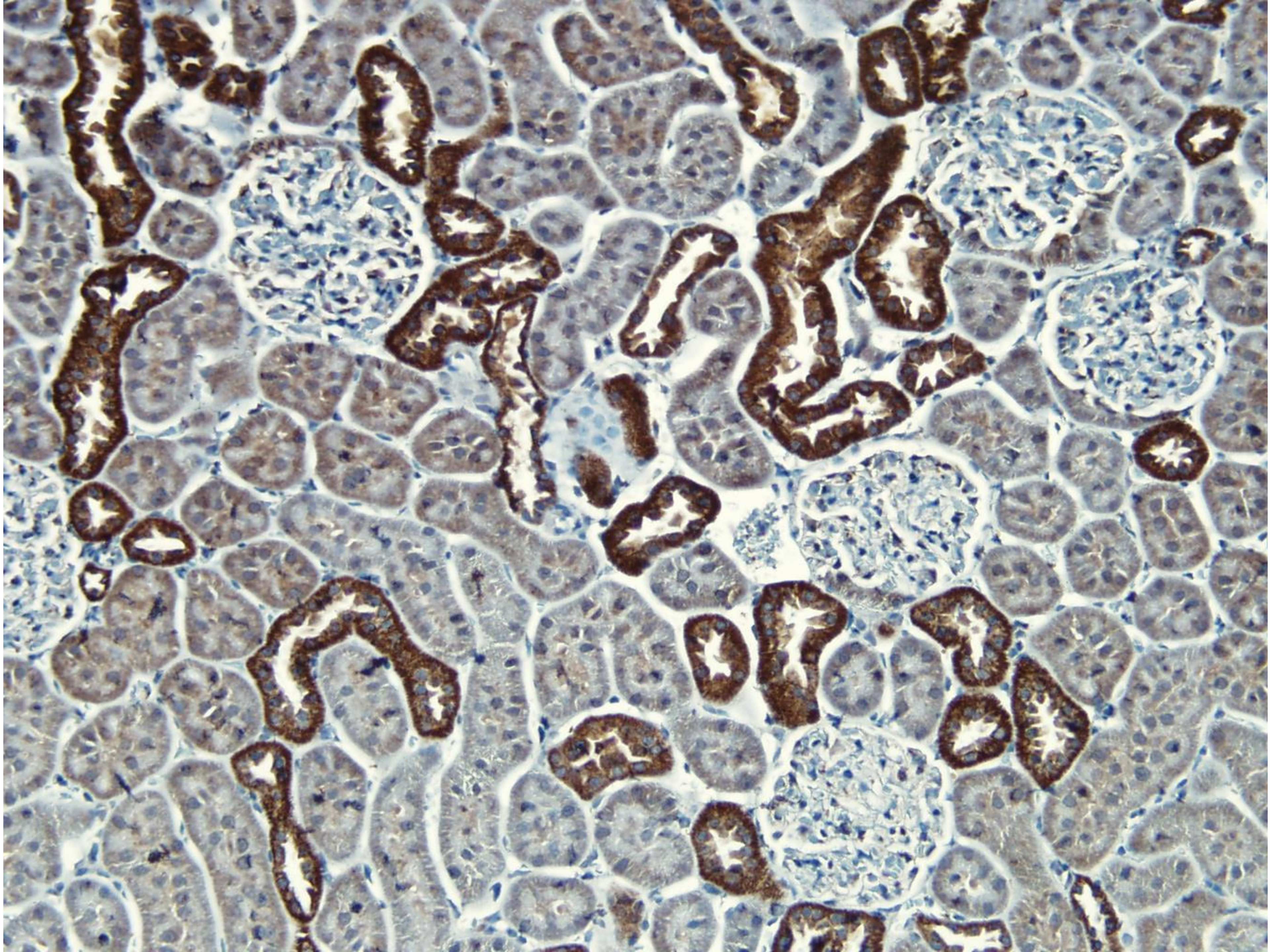

Female 7D I/R

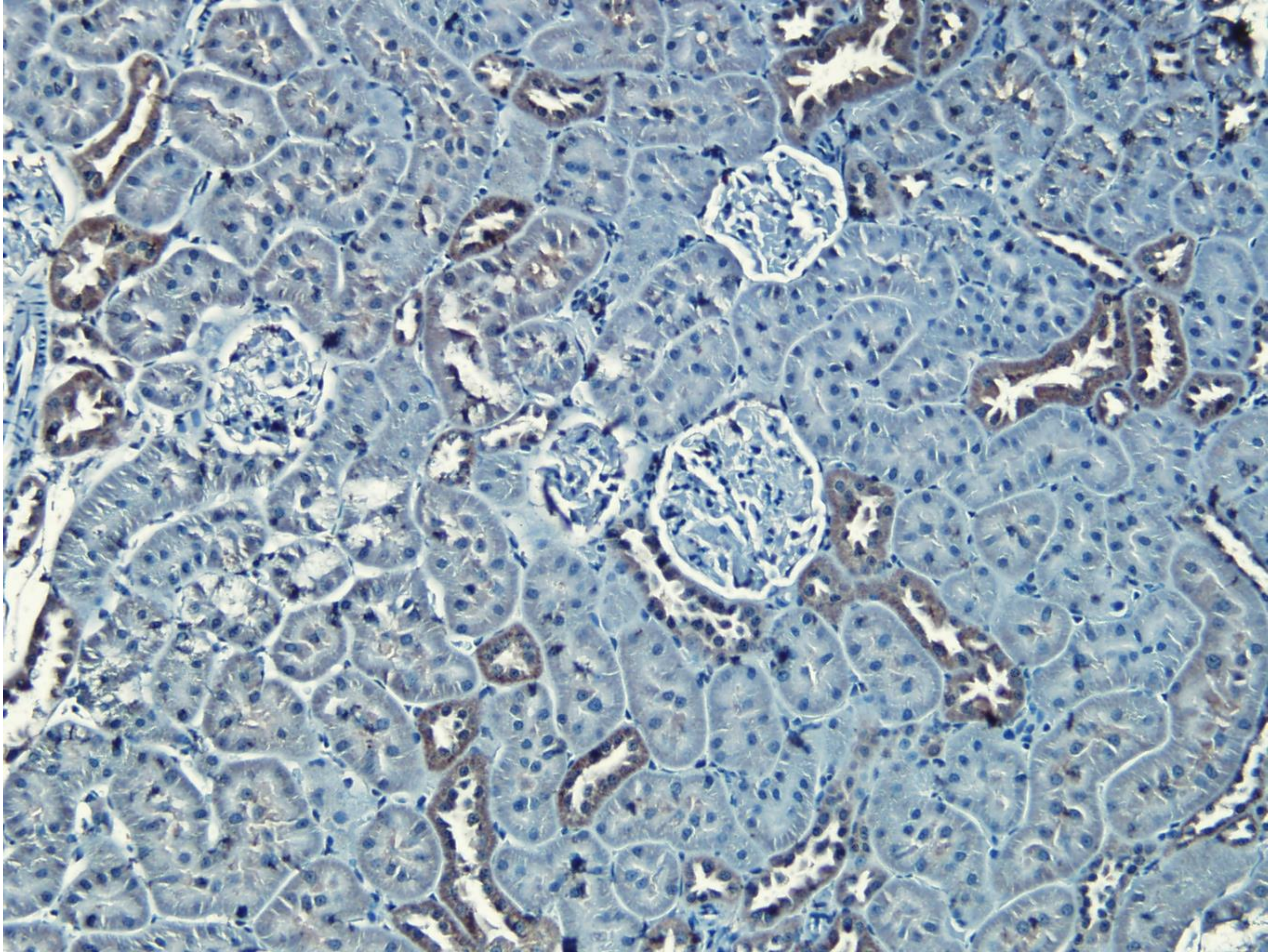

Male Sham

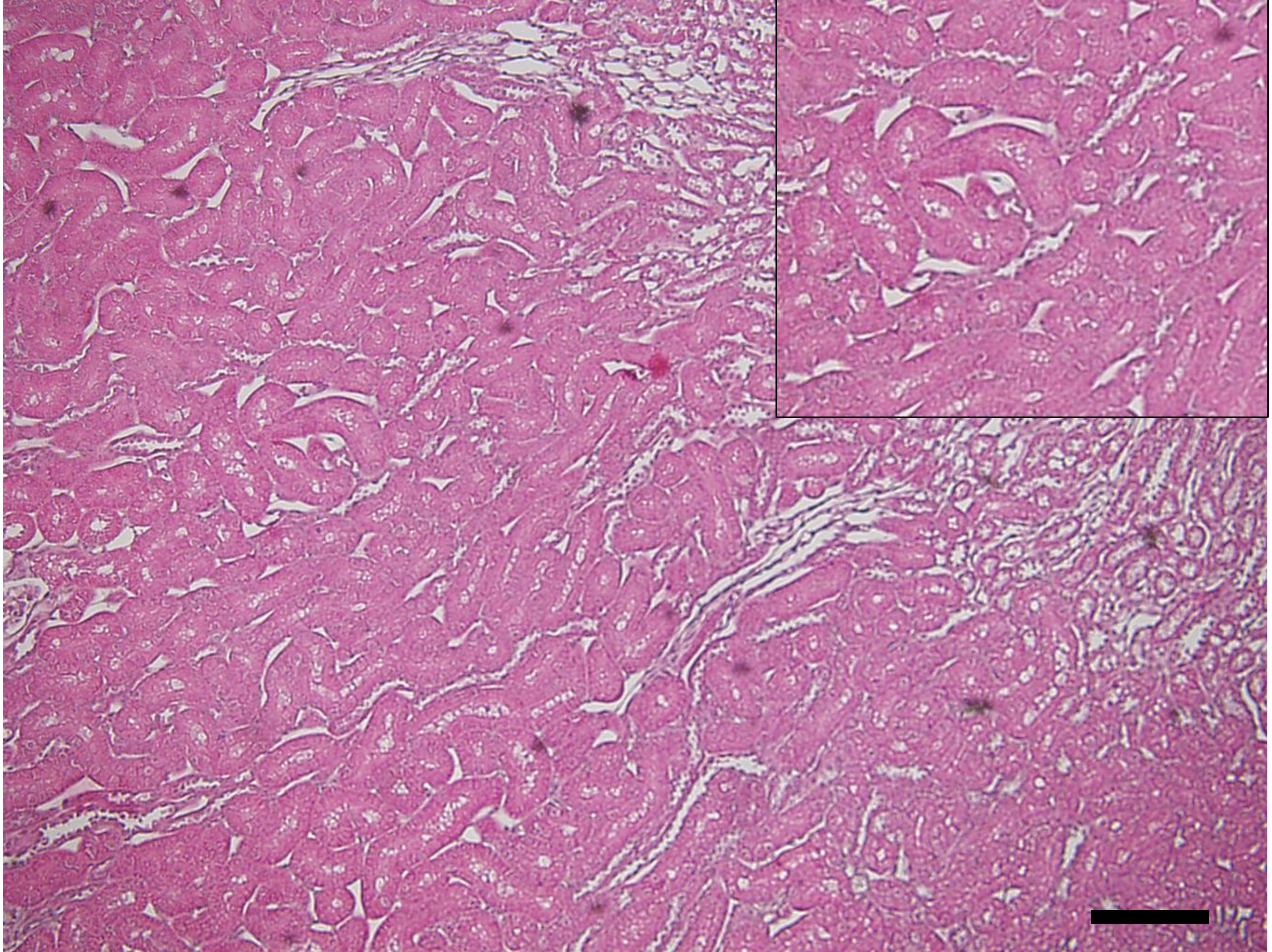

Male 7D I/R

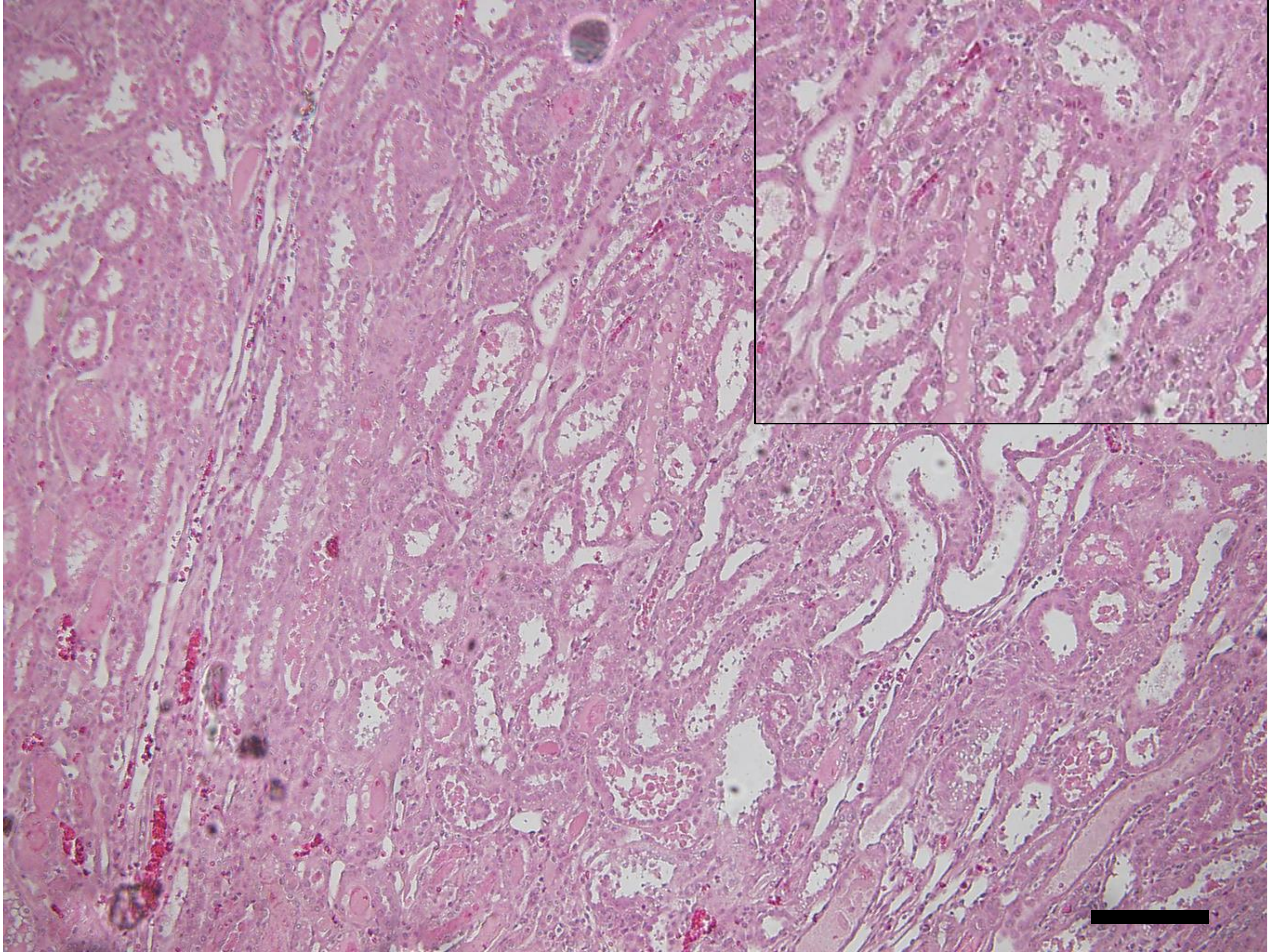

Male Sham + ML355 7D

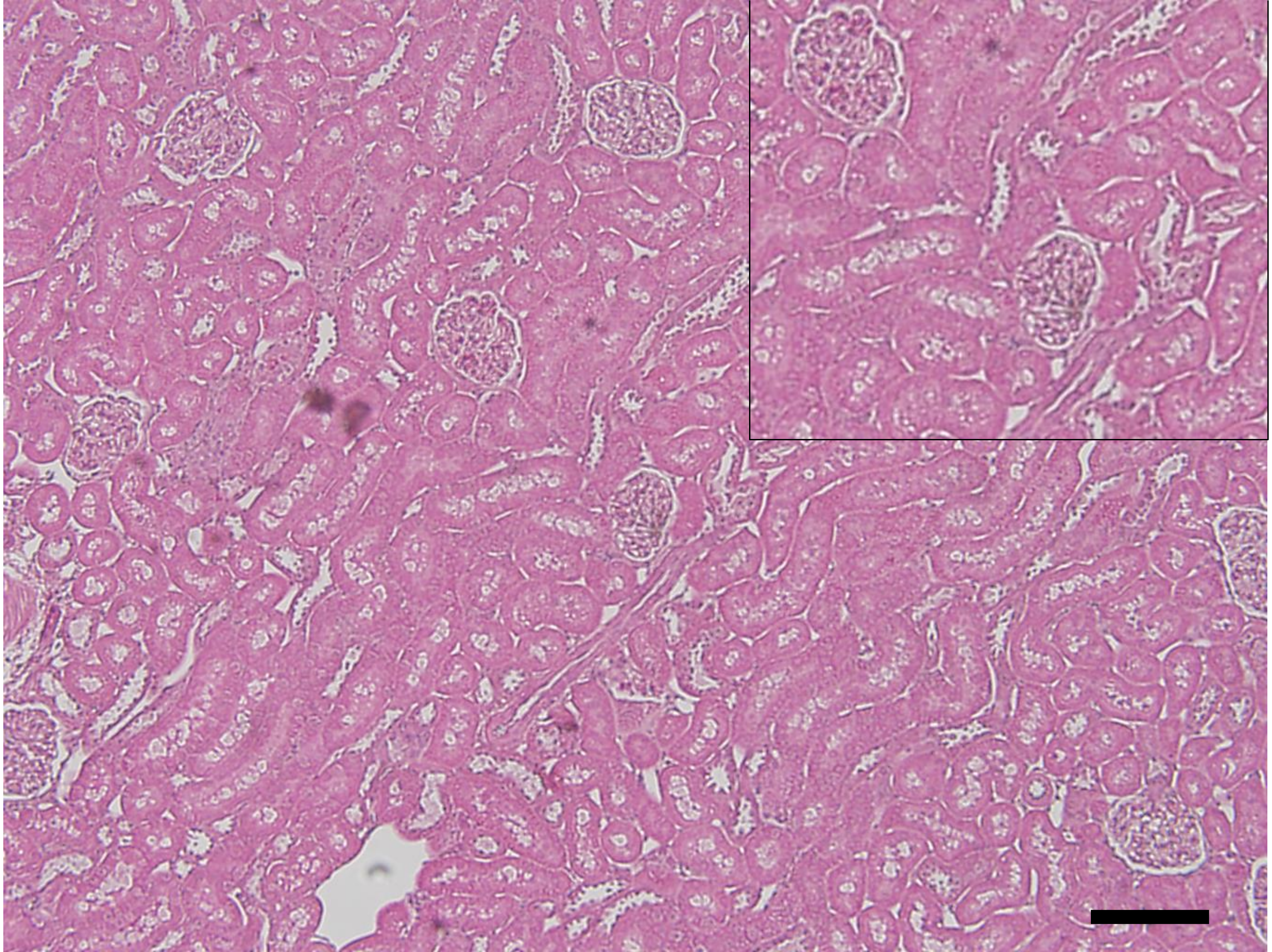

Male 7D I/R + ML355

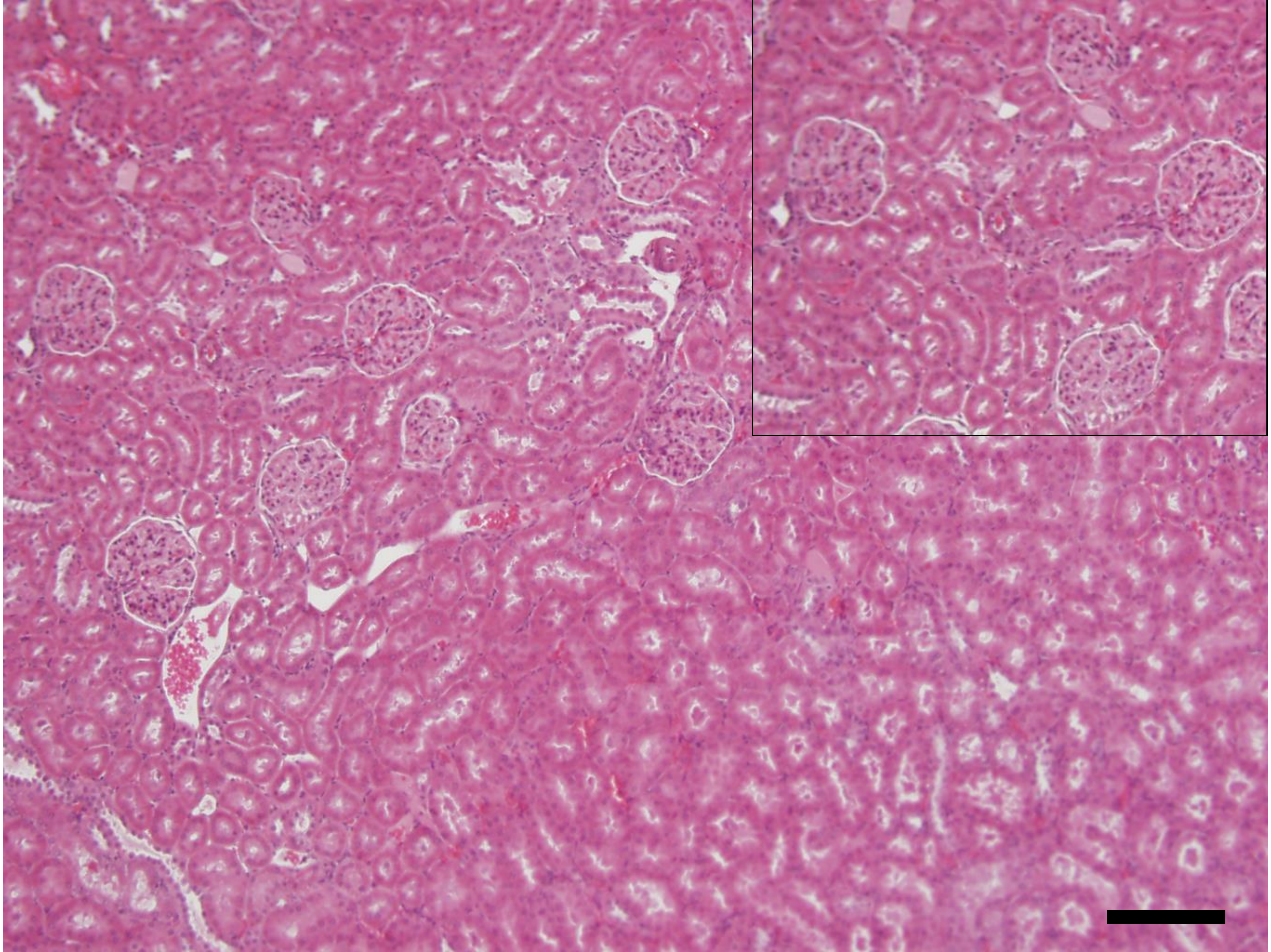

Female Sham

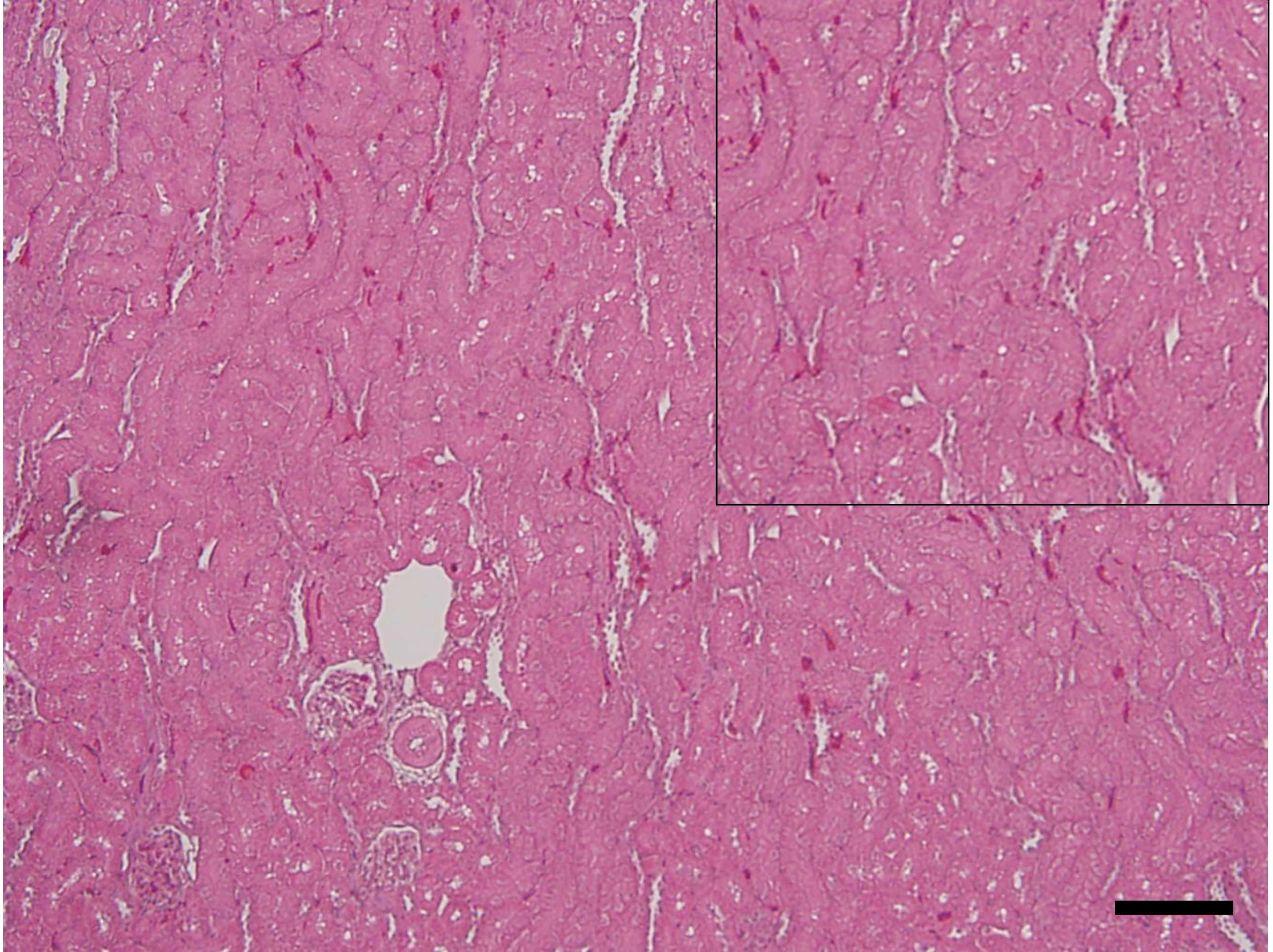

Female IR 7D

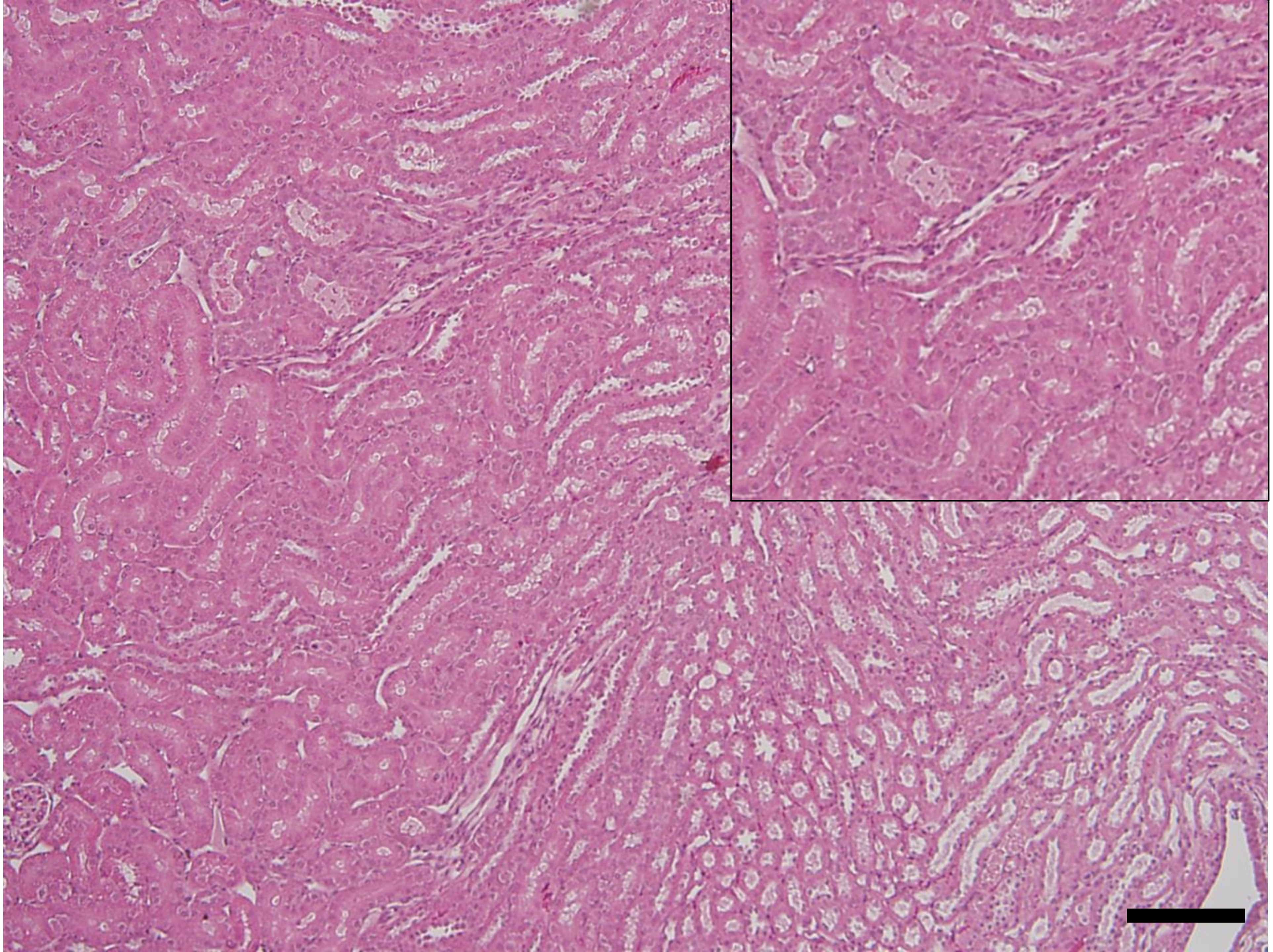

Female Sham +
ML355 7D

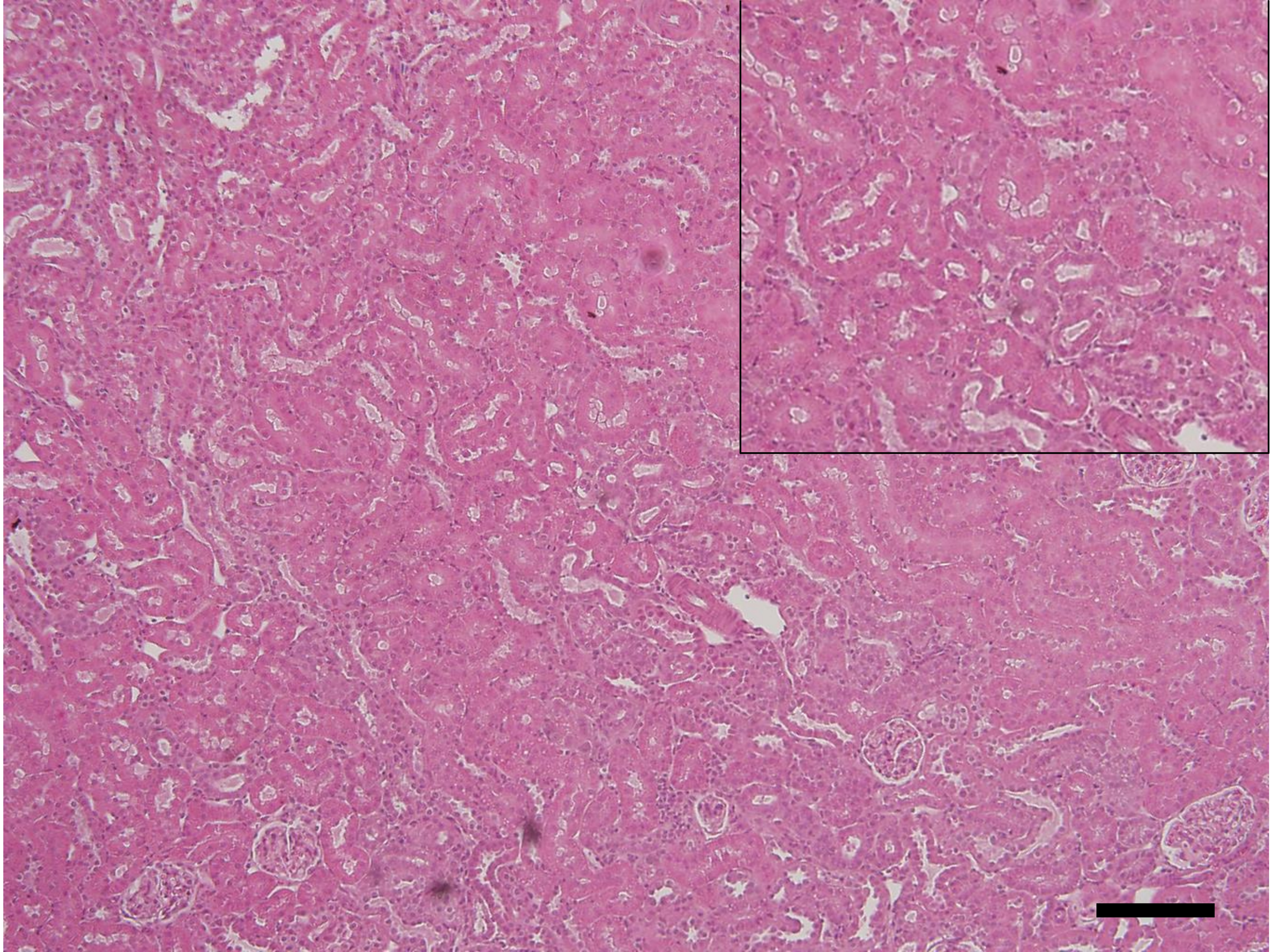

Female I/R 7D + ML355

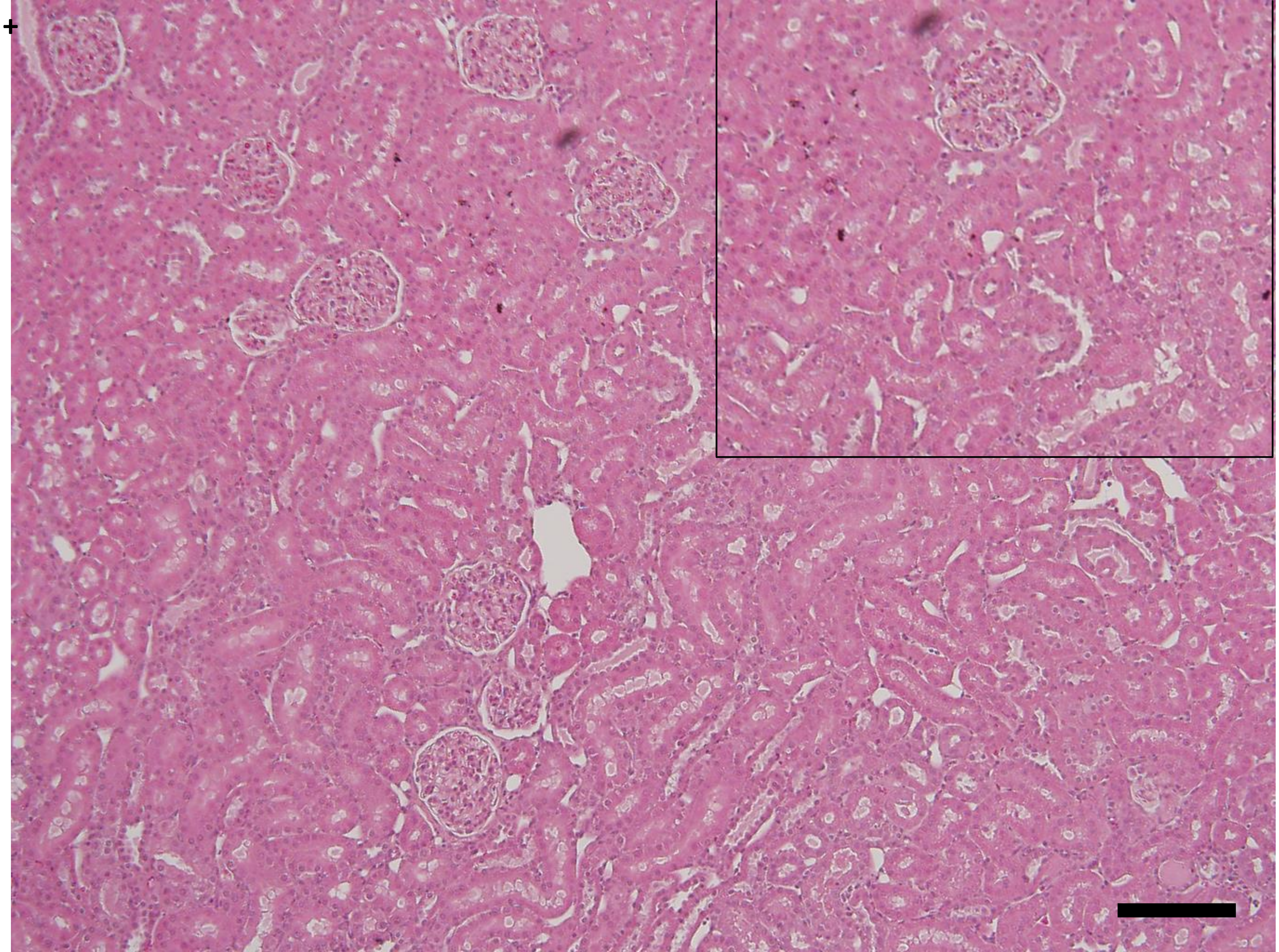

Male Sham

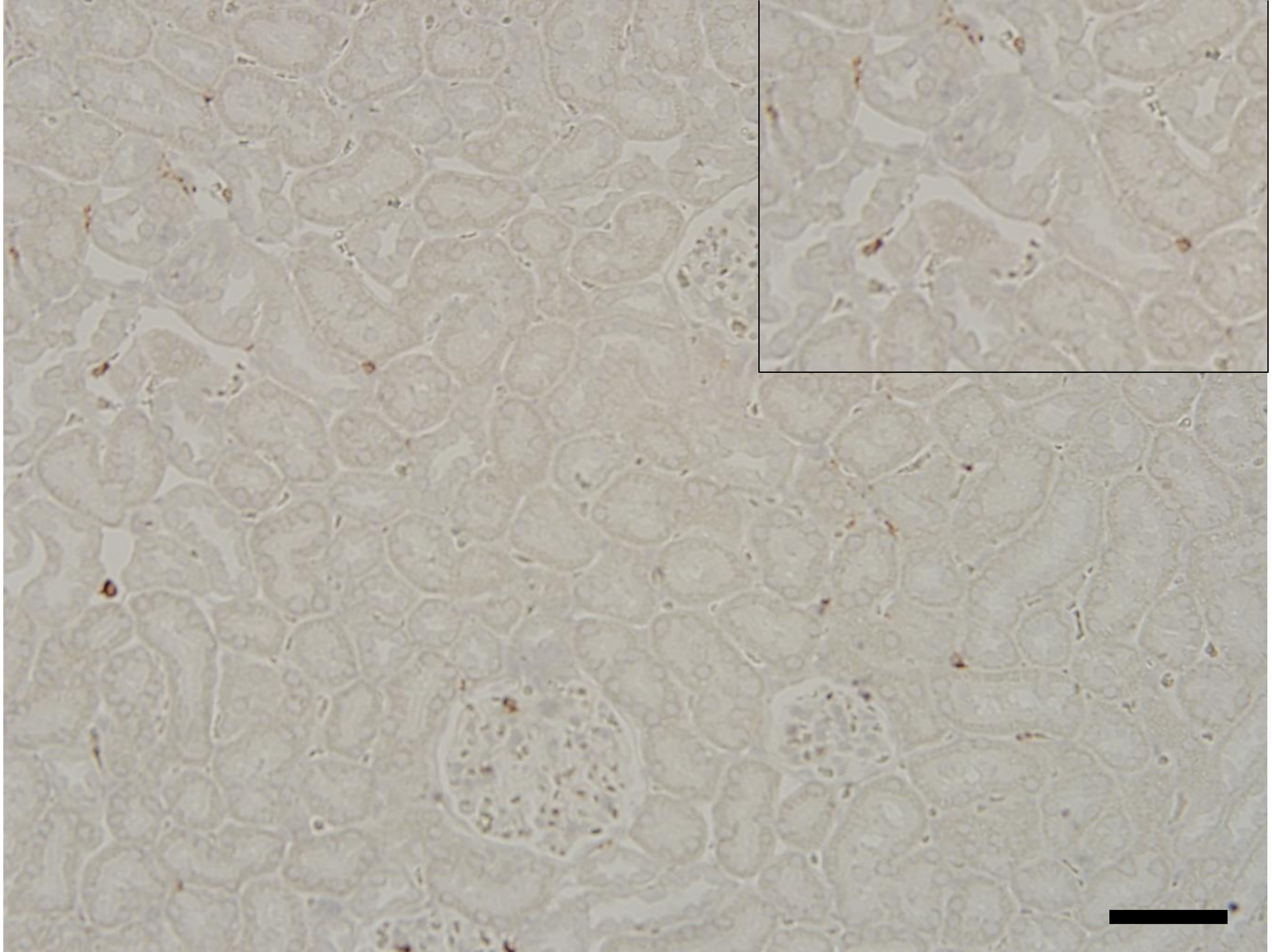

Male 7D I/R

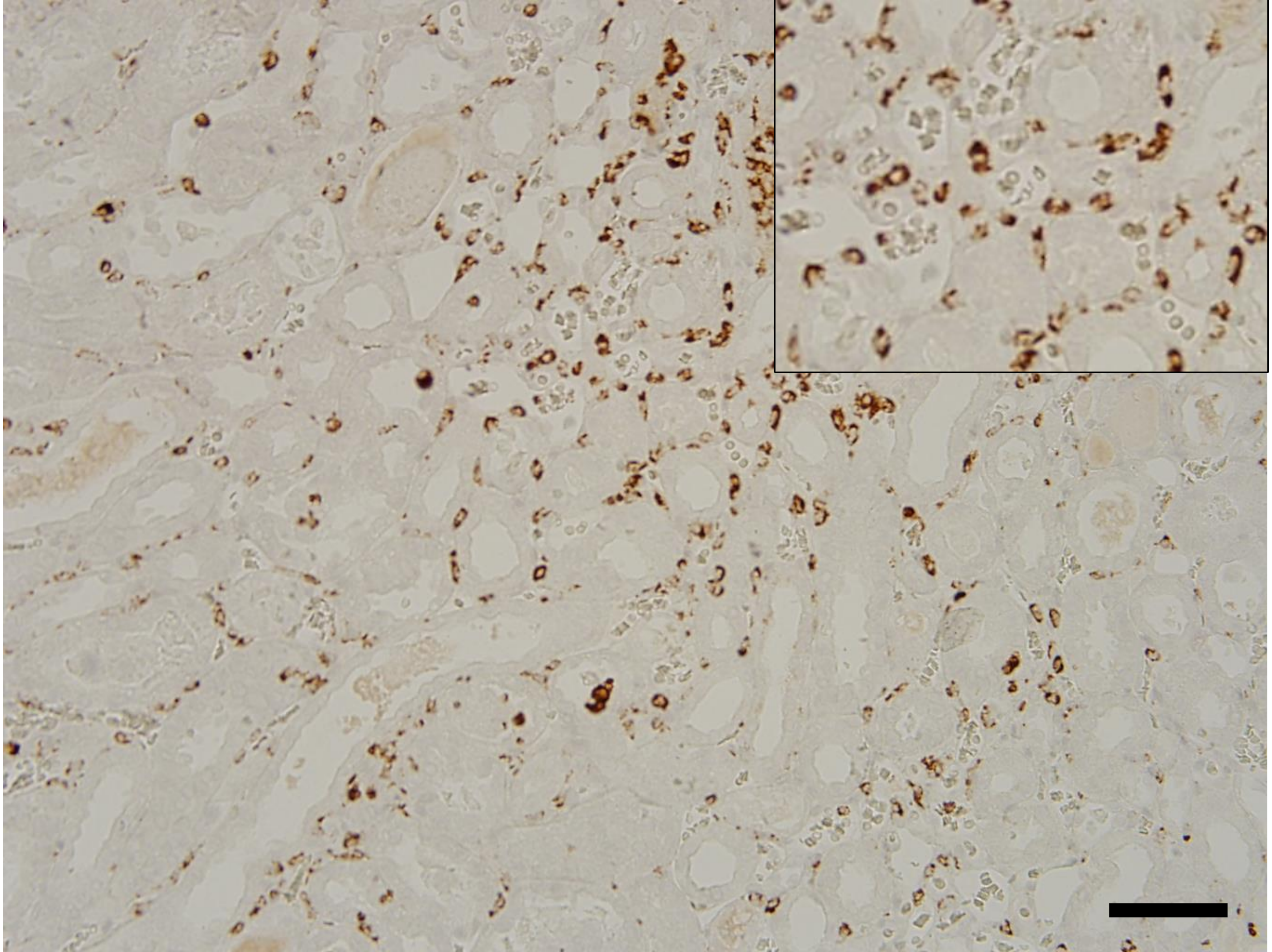

Male Sham + ML355 7D

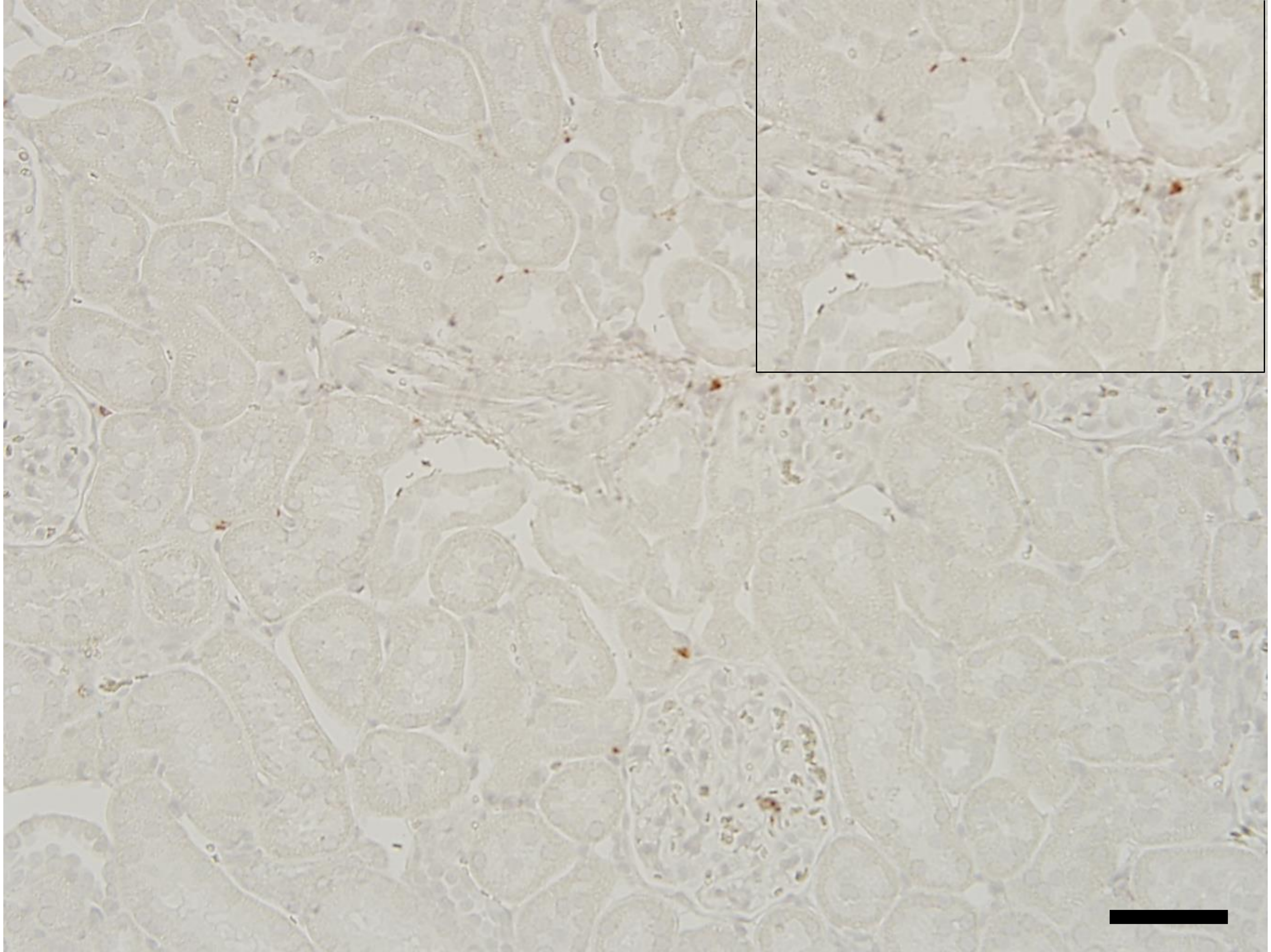

Male 7D I/R + ML355

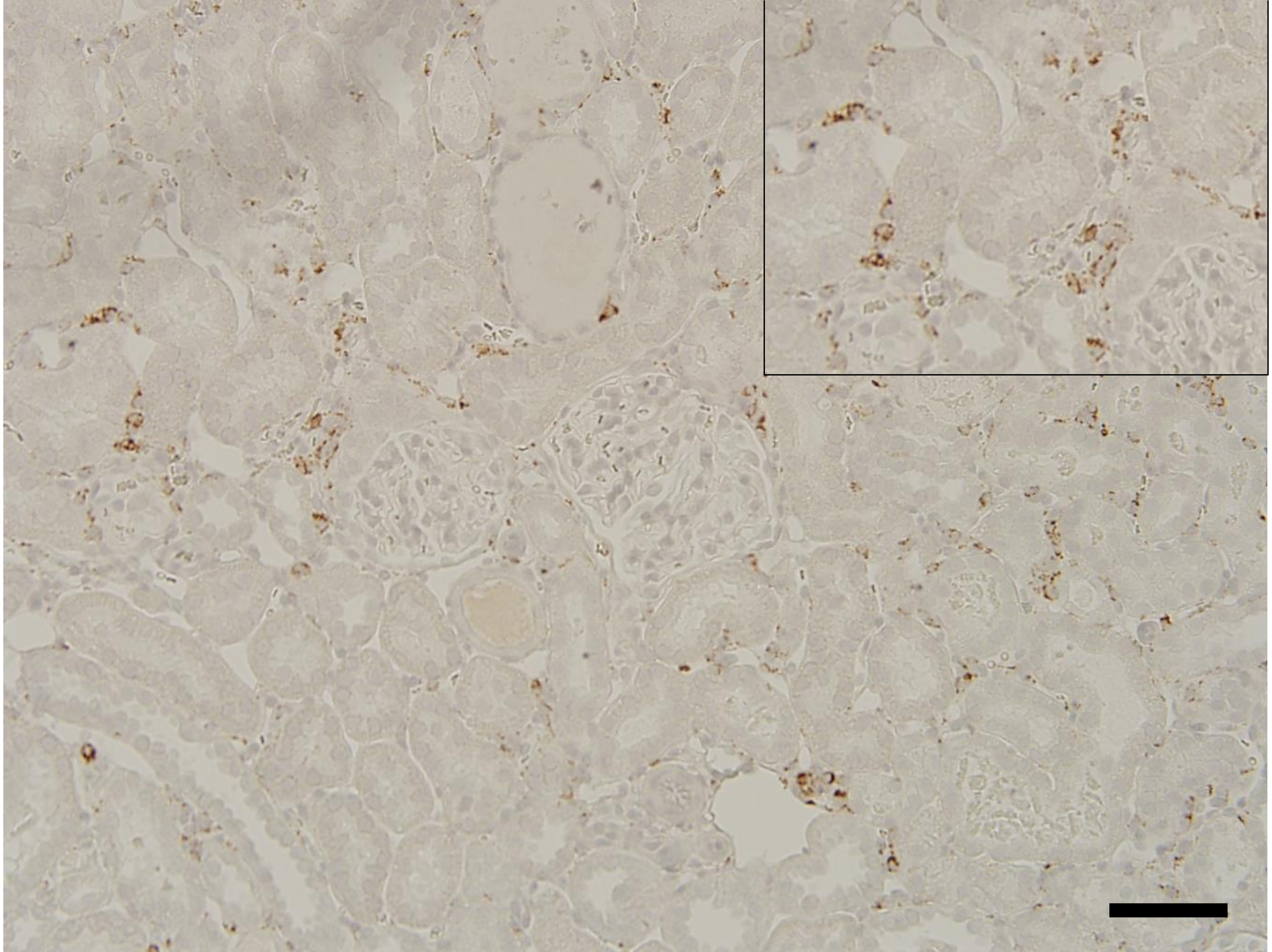

Female Sham

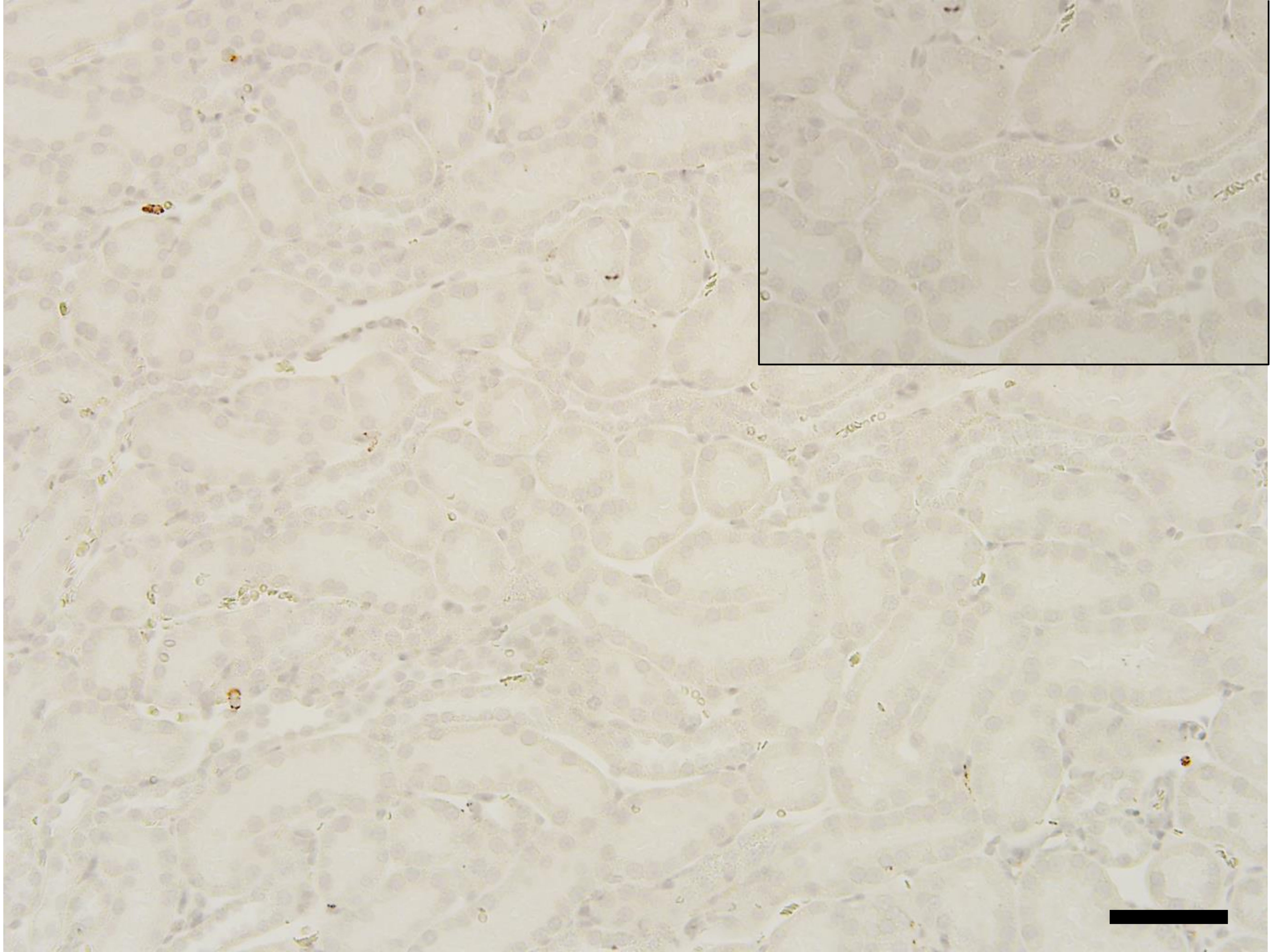

Female IR 7D

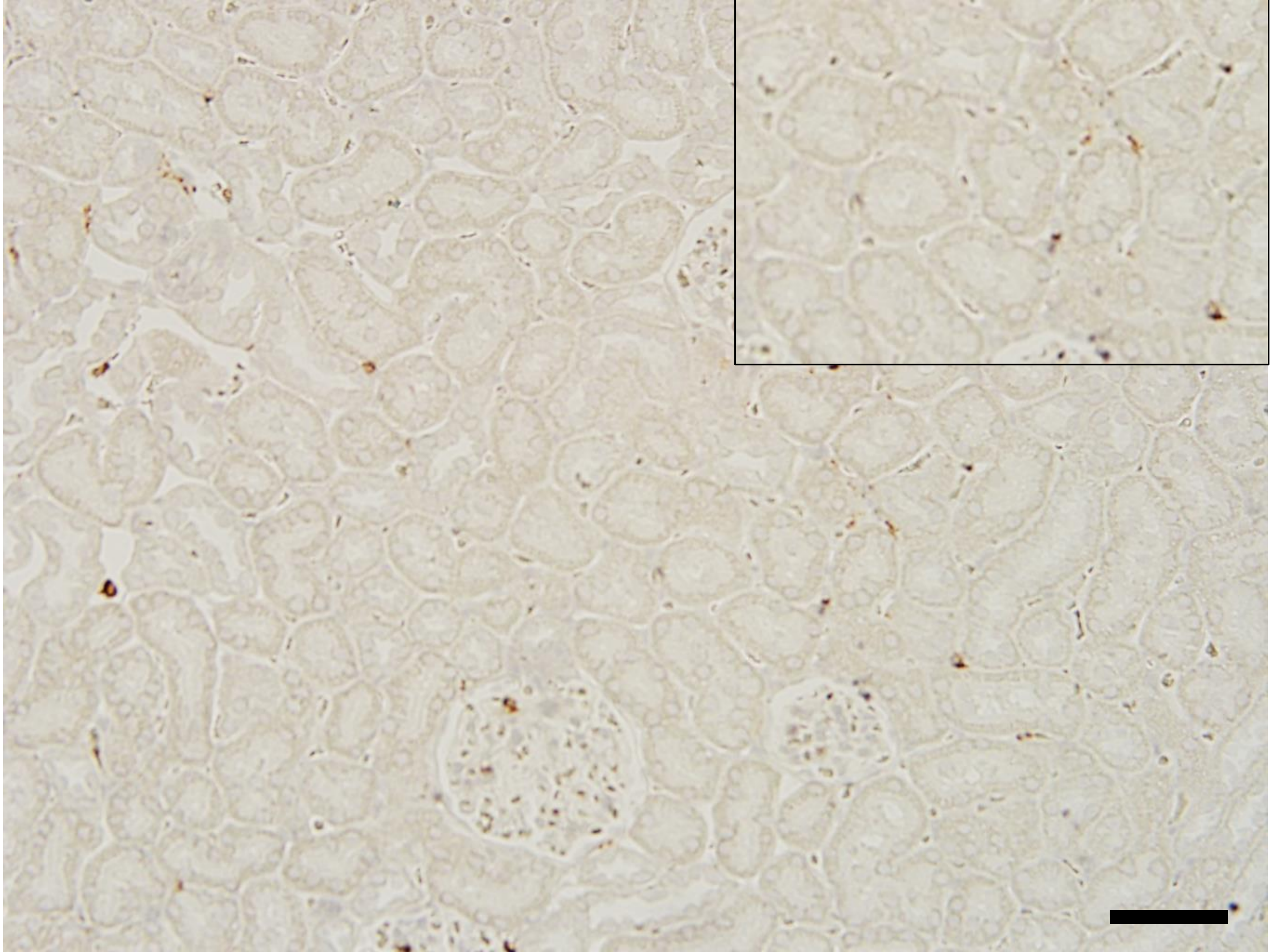

Female Sham +
ML355 7D

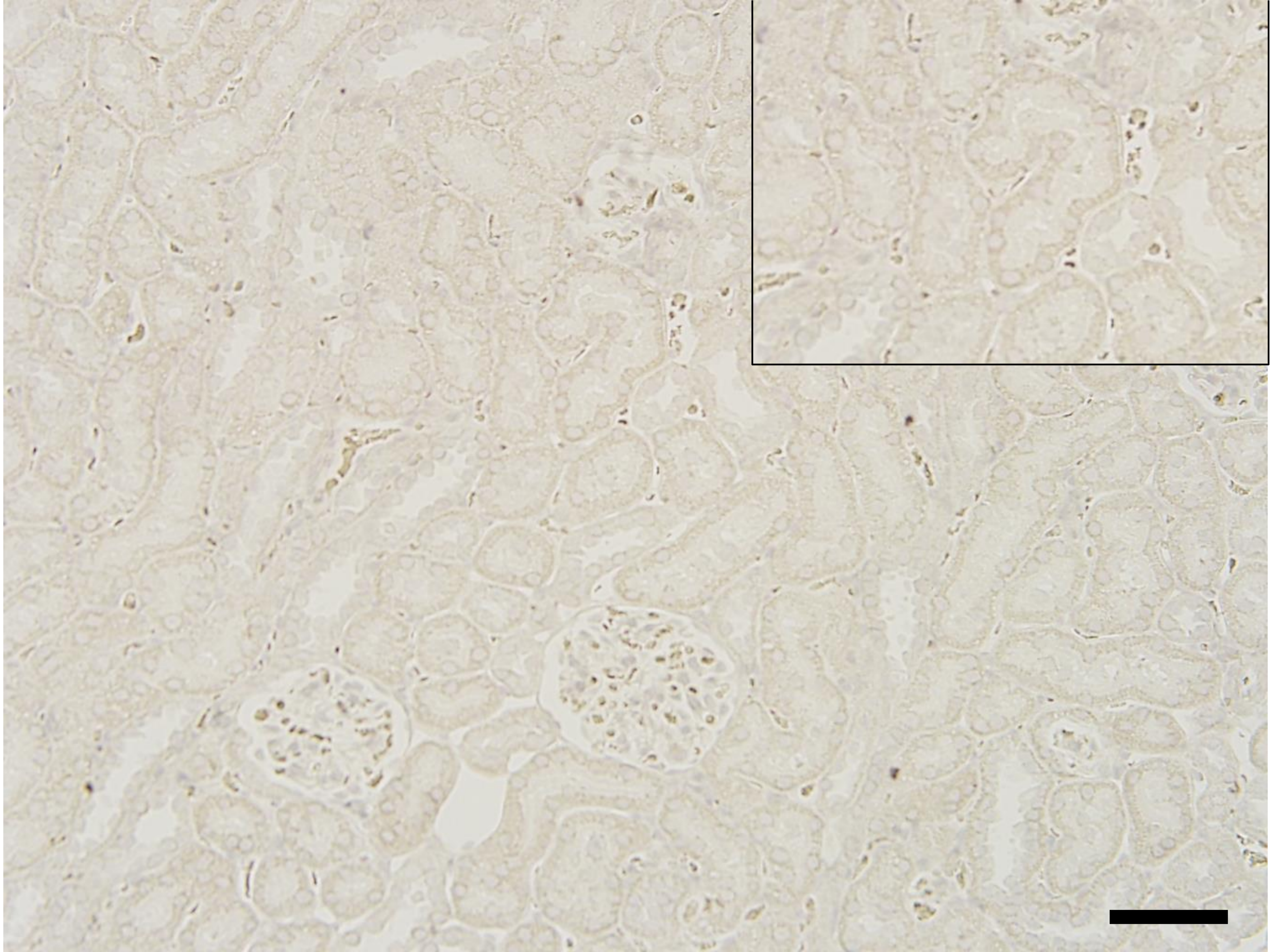

Female I/R 7D + ML355

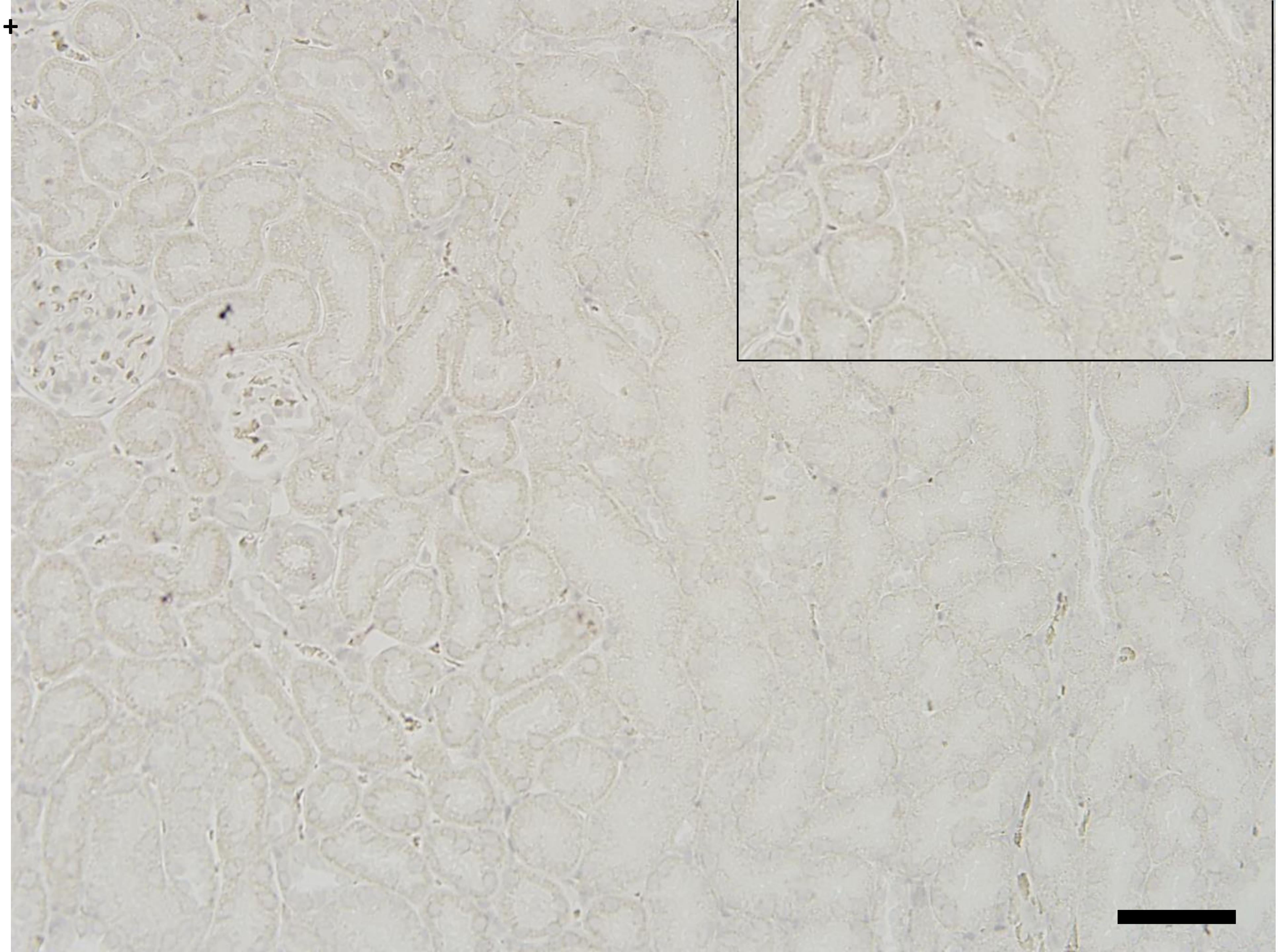

Male 7D I/R

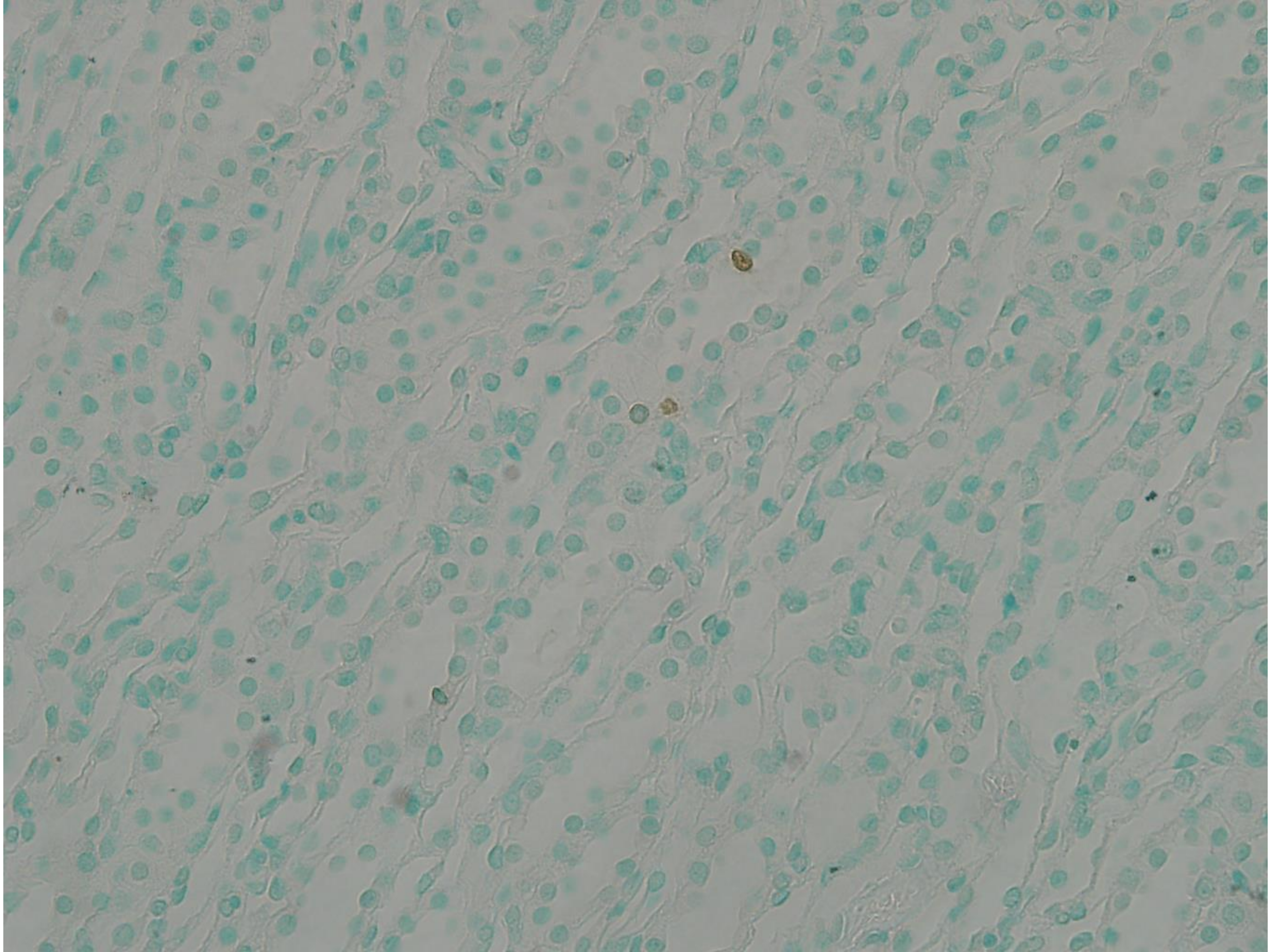

Male 7D I/R

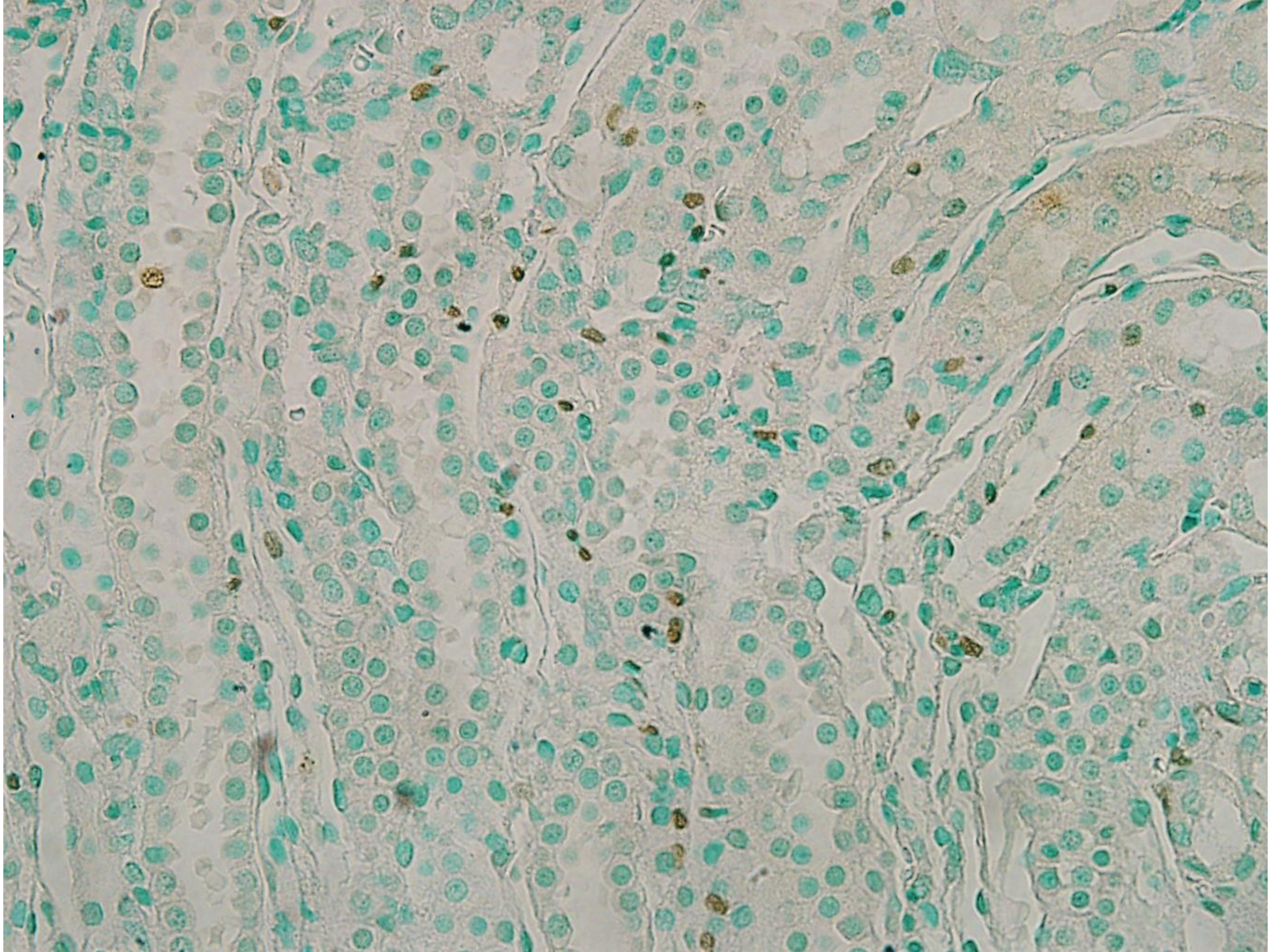

**Male Sham +
ML355 7D**

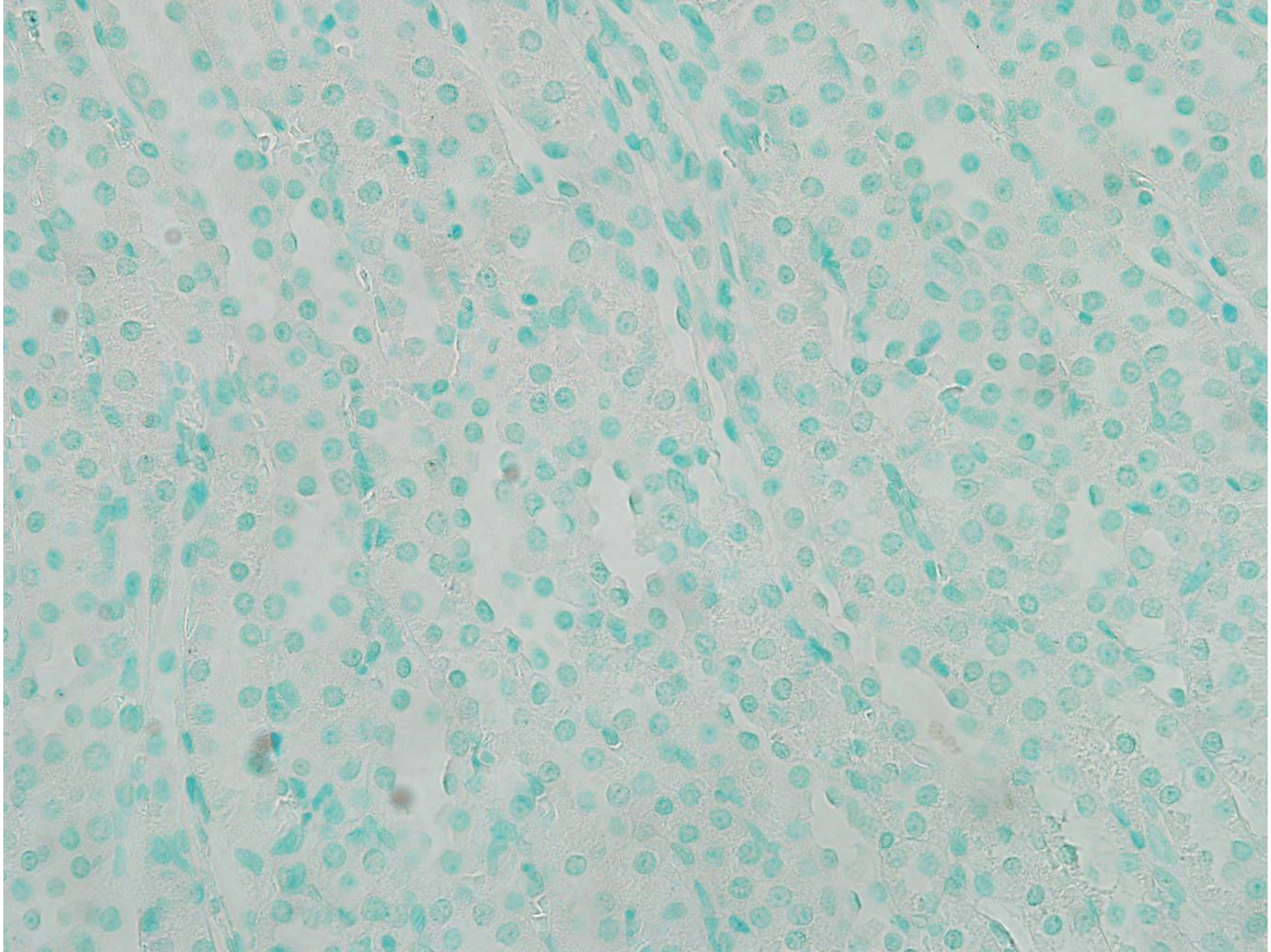

Male 7D I/R +
ML355

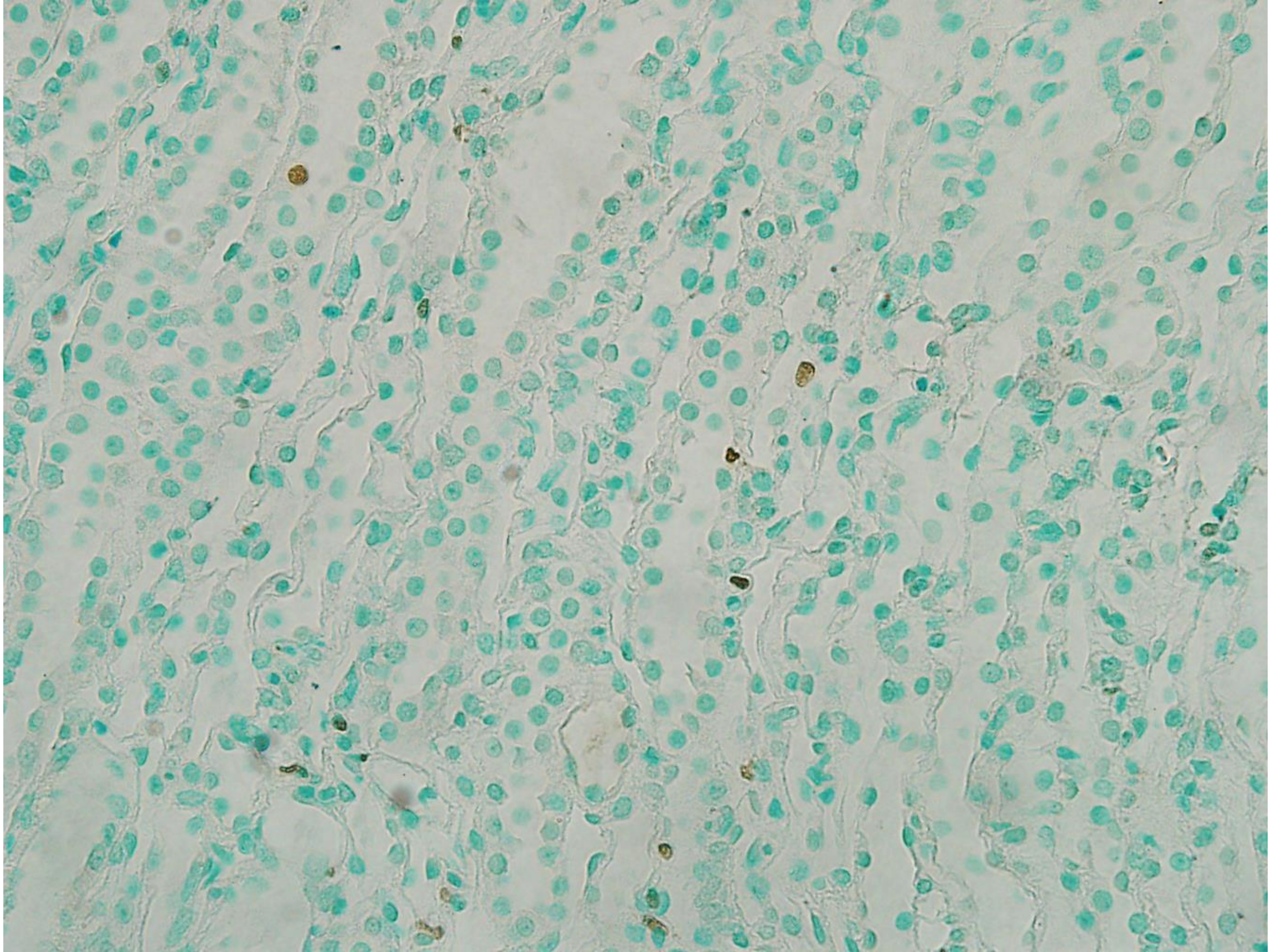

Female Sham

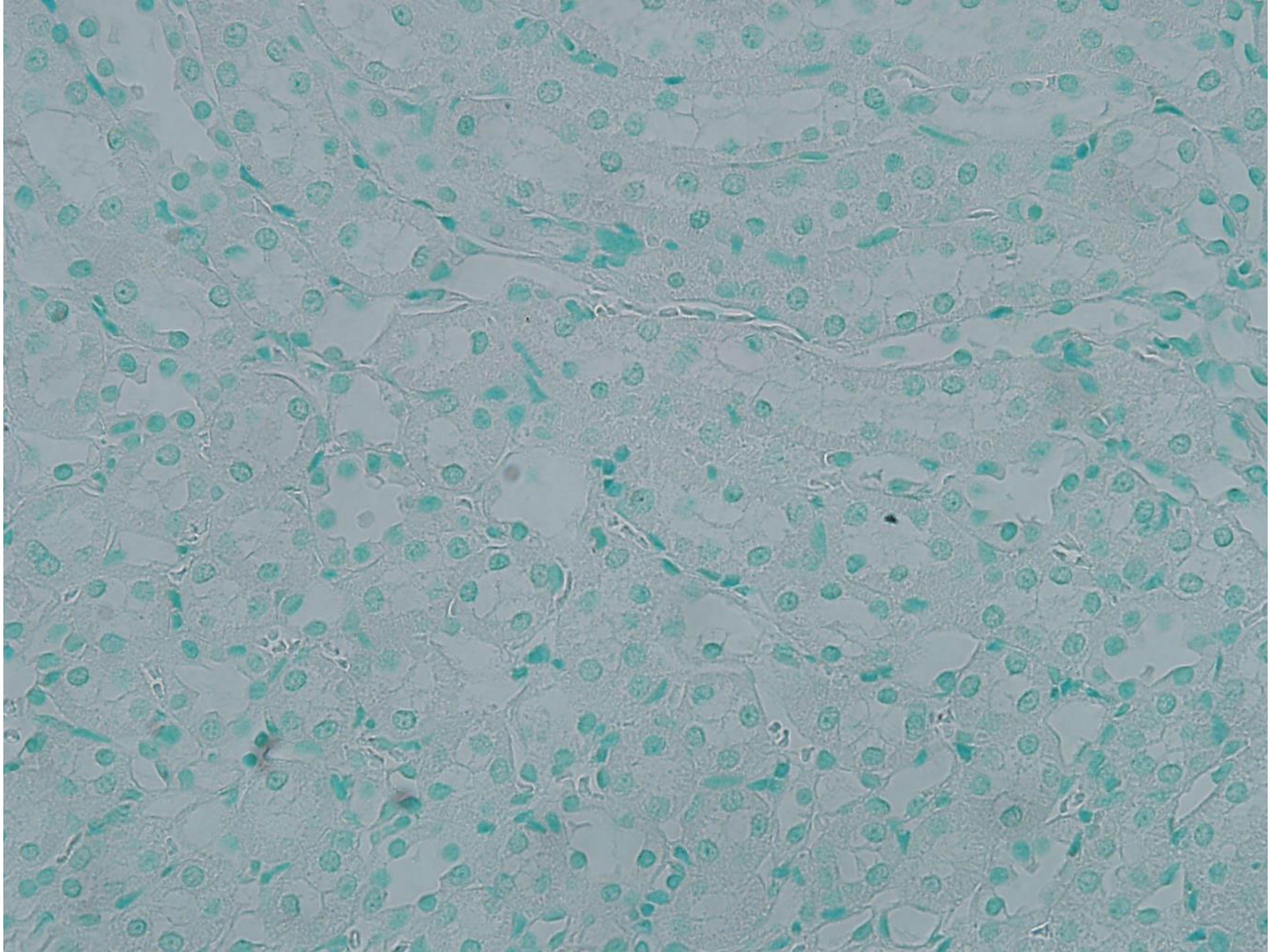

Female IR 7D

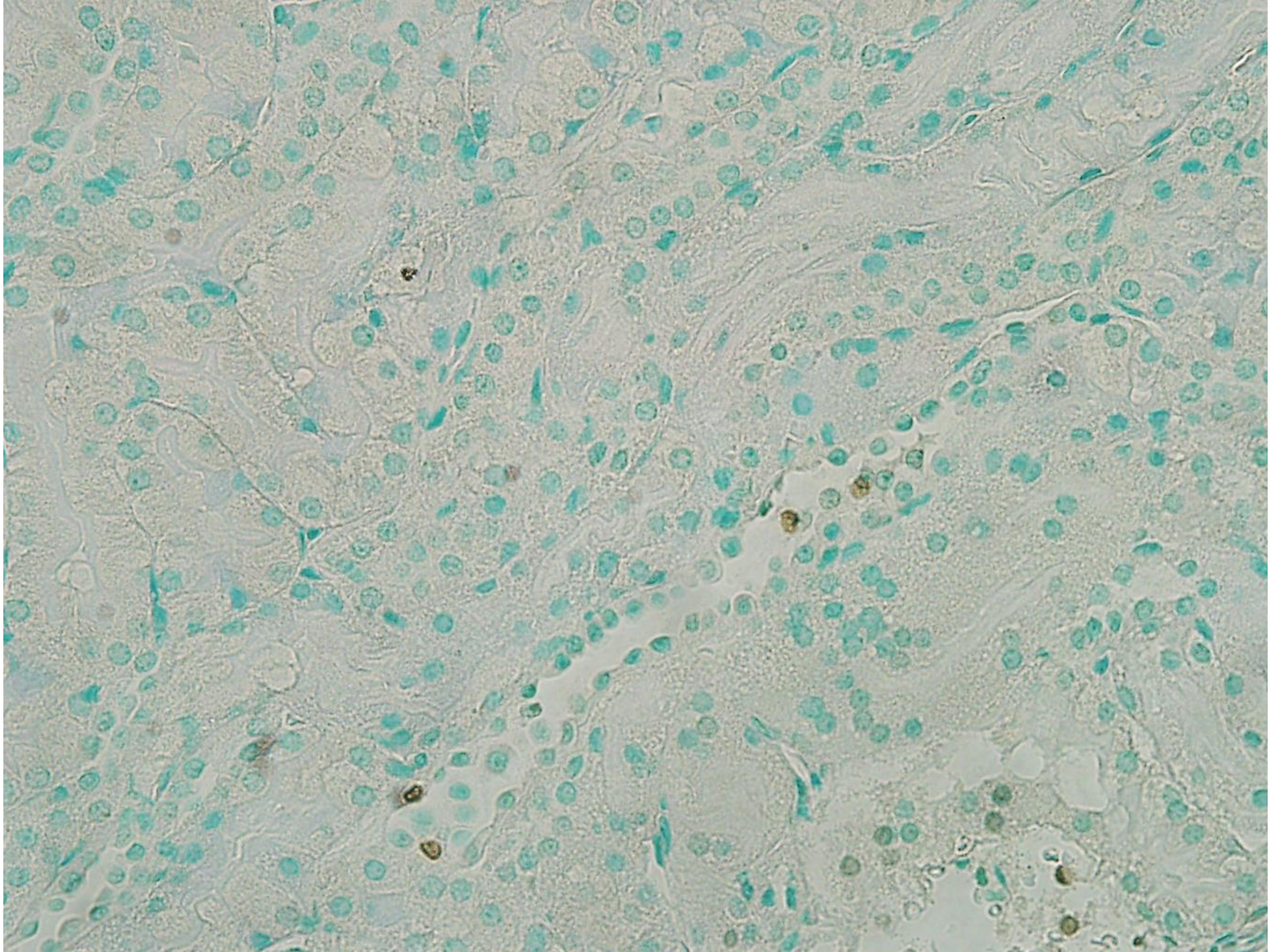

Female Sham + ML355 7D

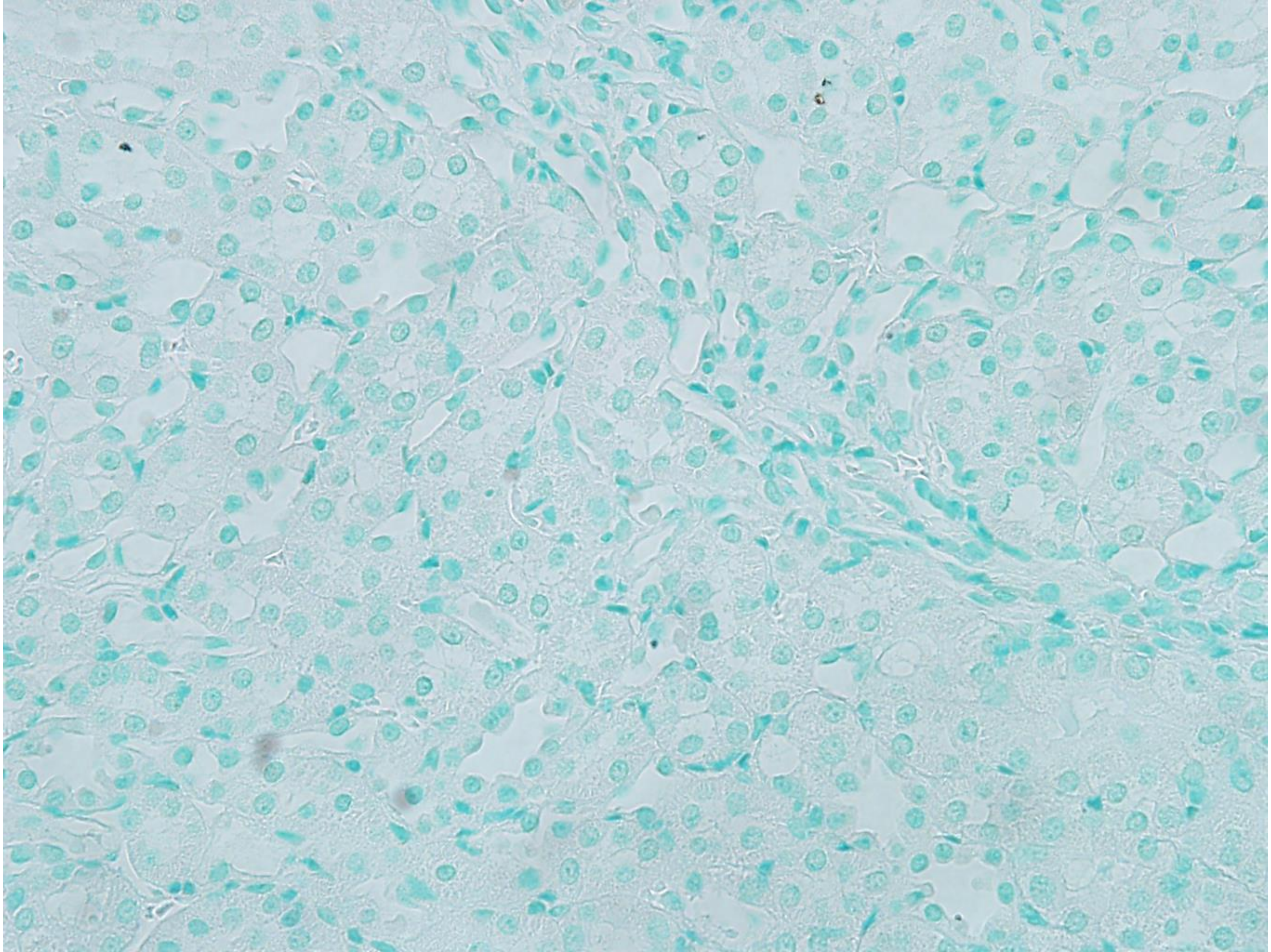

Female I/R 7D +
ML355

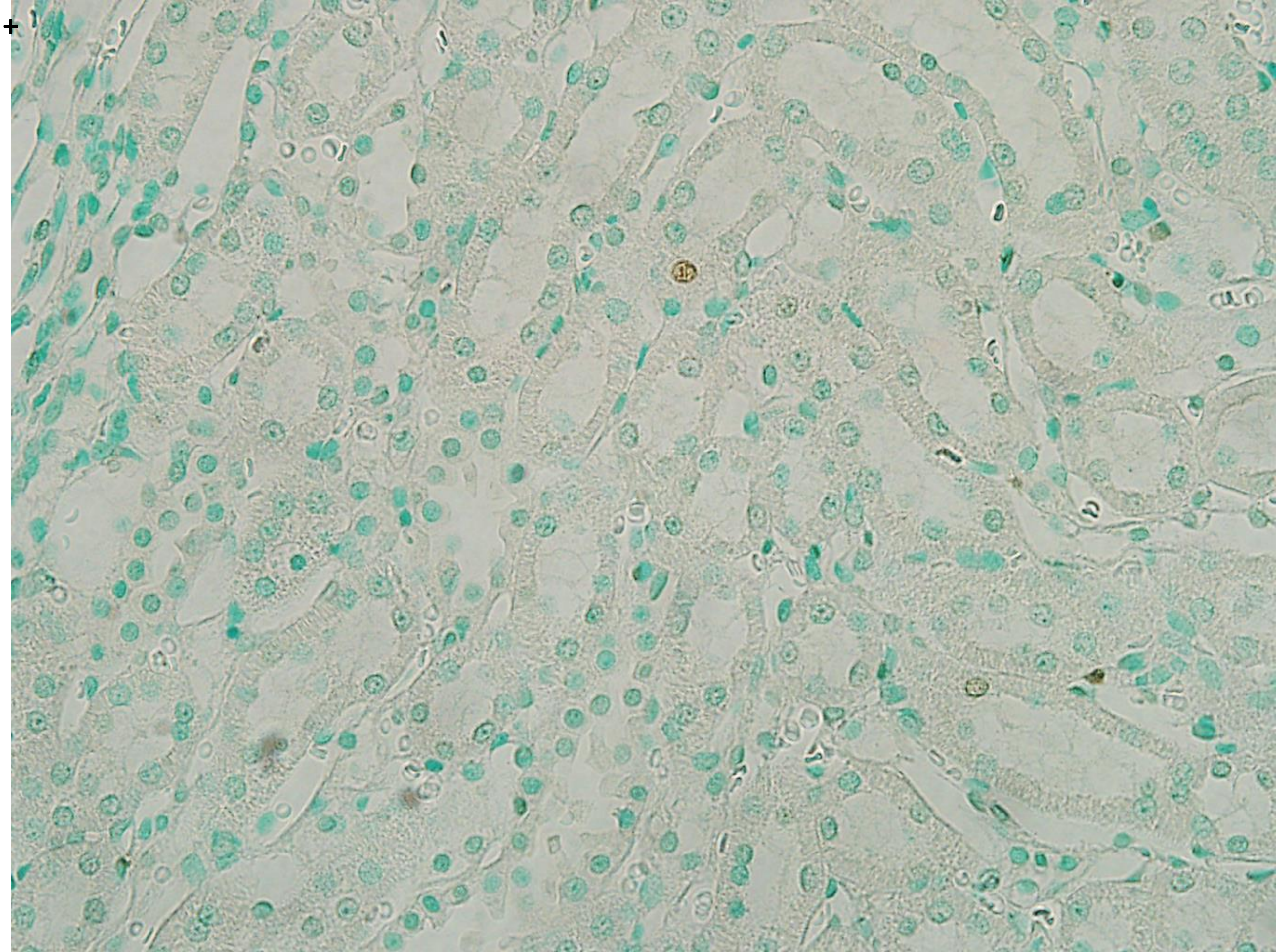

Supplement: Supplementary file 2 — Supplementary Material 2: Original western blot gels (unprocessed) and microscopy images associated with manuscript [file 10020_2023_762_MOESM2_ESM.pdf]
